# Effectiveness of chatbots on COVID vaccine confidence and acceptance in Thailand, Hong Kong, and Singapore

**DOI:** 10.1038/s41746-023-00843-6

**Published:** 2023-05-25

**Authors:** Kristi Yoonsup Lee, Saudamini Vishwanath Dabak, Vivian Hanxiao Kong, Minah Park, Shirley L. L. Kwok, Madison Silzle, Chayapat Rachatan, Alex Cook, Aly Passanante, Ed Pertwee, Zhengdong Wu, Javier A. Elkin, Heidi J. Larson, Eric H. Y. Lau, Kathy Leung, Joseph T. Wu, Leesa Lin

**Affiliations:** 1 Laboratory of Data Discovery for Health (D24H), Hong Kong Science Park, Hong Kong Special Administrative Region, Hong Kong, China; 2 WHO Collaborating Centre for Infectious Disease Epidemiology and Control, School of Public Health, LKS Faculty of Medicine, The University of Hong Kong, Hong Kong SAR, China; 3 Health Intervention and Technology Assessment Program, Ministry of Public Health, Nonthaburi, Thailand; 4 Department of Health Convergence, Ewha Womans University, Seoul, Korea; 5 Saw Swee Hock School of Public Health, National University of Singapore and National University Health System, Singapore, Singapore; 6 Department of Infectious Disease Epidemiology, London School of Hygiene and Tropical Medicine, London, UK; 7 Department of Digital Health and Innovation, World Health Organization, Genève, Switzerland; 8 Institute for Health Metrics and Evaluation, University of Washington, Seattle, WA USA; 9 The University of Hong Kong—Shenzhen Hospital, Shenzhen, China

**Keywords:** Public health, Communication

## Abstract

Chatbots have become an increasingly popular tool in the field of health services and communications. Despite chatbots’ significance amid the COVID-19 pandemic, few studies have performed a rigorous evaluation of the effectiveness of chatbots in improving vaccine confidence and acceptance. In Thailand, Hong Kong, and Singapore, from February 11th to June 30th, 2022, we conducted multisite randomised controlled trials (RCT) on 2,045 adult guardians of children and seniors who were unvaccinated or had delayed vaccinations. After a week of using COVID-19 vaccine chatbots, the differences in vaccine confidence and acceptance were compared between the intervention and control groups. Compared to non-users, fewer chatbot users reported decreased confidence in vaccine effectiveness in the Thailand child group [Intervention: 4.3 % vs. Control: 17%, *P* = 0.023]. However, more chatbot users reported decreased vaccine acceptance [26% vs. 12%, *P* = 0.028] in Hong Kong child group and decreased vaccine confidence in safety [29% vs. 10%, *P* = 0.041] in Singapore child group. There was no statistically significant change in vaccine confidence or acceptance in the Hong Kong senior group. Employing the RE-AIM framework, process evaluation indicated strong acceptance and implementation support for vaccine chatbots from stakeholders, with high levels of sustainability and scalability. This multisite, parallel RCT study on vaccine chatbots found mixed success in improving vaccine confidence and acceptance among unvaccinated Asian subpopulations. Further studies that link chatbot usage and real-world vaccine uptake are needed to augment evidence for employing vaccine chatbots to advance vaccine confidence and acceptance.

## Introduction

As of January 2022, >500 million confirmed cases of COVID-19 have been reported worldwide^1^. COVID-19 vaccines have been proven to be effective in lowering hospitalisation and mortality rates^2^, and are considered the most efficient tools for reducing severe disease and economic burden^3,4^. However, by January 28th, 2022, only 17 countries worldwide had attained a vaccination coverage of 70 percent^4^.

Many factors contribute to low COVID-19 vaccination coverage, including vaccine supply and distribution, access to healthcare facilities, and vaccine hesitancy. In particular, vaccine hesitancy, named one of the ten biggest threats to global health by the World Health Organization (WHO)^5^, refers to the “motivational state of being conflicted about, or opposed to, getting vaccinated.”^6^ Vaccine hesitancy is a major obstacle to increasing vaccine uptake and returning to normalcy, especially among seniors and parents of children^7–10^. As of January 28th, 2022, immediately preceding the study period, the percentage of elderly people aged 60 and above who had received at least one dose of vaccine was 75.1% in Thailand and 45% in Hong Kong; vaccination coverage of at least one dose among children was 34.5% in Hong Kong and 44% in Singapore, while Thailand had not yet begun vaccinating children^11–14^.

Factors affecting COVID-19 vaccine hesitancy included perceptions of vaccine importance, efficacy and safety, concerns about side effects, vaccine accessibility, and personal or religious beliefs^15–18^. Major concerns from seniors regarded the risk of serious adverse events following immunisation, such as deaths and complications due to old age and medical history^19^. For example, in Thailand, vaccines were provided by both the government and the private sector; however, results from cross-sectional surveys indicated that vaccination uptake among Thai people, especially among seniors, was low compared to other Southeast Asian countries^20^. Further, as children became newly eligible for vaccinations, there were concerns over the necessity or safety of child vaccinations. A large-scale study showed that Thai parents’ willingness to get their children vaccinated was the lowest among lower- and middle-income countries, due to uncertainty about COVID-19 vaccines’ effectiveness and safety^21^. Likewise, Hong Kong parents had doubts about vaccine safety for their children, and Hong Kong caregivers’ willingness to have their children vaccinated was lower compared to other countries^22^. In Singapore, some parents voiced concerns about the risk of contracting COVID-19 despite taking their children to get vaccinated^23^. Studies showed that low vaccine uptake in the region could be linked to high complacency from relatively low numbers of daily COVID-19 cases and low confidence in vaccine safety, coupled with a lack of knowledge about differences between each vaccine type^10,20–24^.

Compared to previous infectious disease outbreaks, a significant challenge during the COVID-19 pandemic was the overabundance of online and offline information, which included deliberate attempts to undermine and jeopardize global pandemic response measures by disseminating misinformation^25^. In response, the need for timely vaccination communication called for more effective use of social media and digital technologies such as machine learning, artificial intelligence, and conversation technology^26^. Among different digital interventions, chatbots have become an increasingly popular tool in health communication and services due to their ubiquitous access points and potential for massive information dissemination. There are many possible benefits to chatbots in an outbreak setting, including the relatively limited resources required to employ them after development, offering accessibility to clear, accurate, and timely information as outbreaks evolve, and freeing up the time of healthcare workers’ to address more complex issues^27^. However, as the use of chatbots in the context of health communication, especially in vaccine communication, is a novel approach, rigorous evaluations of their impact and potential use cases are very limited^28^.

In this study, we test the effectiveness of COVID-19 chatbots on people who were unvaccinated or had delayed vaccinations until the government vaccine mandates in Thailand, Hong Kong, and Singapore. Among many factors affecting vaccine attitudes and behaviours, we focus on participants’ changes in levels of vaccine confidence and acceptance, before and after using the COVID-19 chatbots. Given differentiating findings per target populations, we encourage future studies to further substantiate the evidence of vaccine chatbots’ effectiveness in advancing vaccine confidence and acceptance.

## Results

### Recruitment and retention

**Fig. 1:**
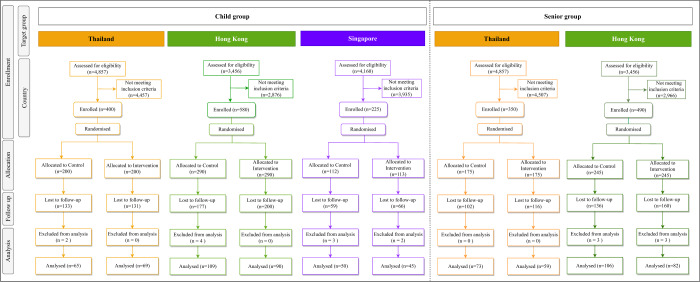
Flow diagram of the randomised controlled trial in Thailand, Hong Kong, and Singapore. Flowchart presenting the number of participants assessed, enrolled, randomized, lost to follow-up, and analysed in each child group and senior group.

From February 11th, 2022 to June 30th, 2022, 2045 participants were enrolled and randomly assigned to the control and intervention groups. After excluding 1,280 participants who were lost to follow-up, responses from 748 participants were included in the final analysis. (Fig. 1 and Supplementary Fig. 8).

### Participant characteristics

Across all five study groups in the three locations, the baseline characteristics between the control and intervention groups were mostly comparable (Supplementary Tables 1 and 2). We noted an overrepresentation of minority subpopulation (i.e., Filipinos and Indonesians) in the Hong Kong and Singapore groups.

### Findings from the randomised controlled trial

In the Thailand child group, results from a one-way comparison using Fisher’s exact test showed that participants in the intervention group were less likely to decrease in confidence regarding vaccine effectiveness: “vaccines are effective” [Intervention: 4.3% vs. Control: 17%, *P* = 0.023], “vaccines are effective regardless of manufacturers” [14% vs. 55%, *P* < 0.001], and “vaccines are effective against all variants” [8.7% vs. 48%, *P* < 0.001] compared to the control group (Table 1). In the Hong Kong child group, we did not detect any statistically significant findings regarding vaccine confidence. However, participants in the intervention group were more likely to report decrease in vaccine acceptance (“has your child received/do you intend for your child to receive a COVID-19 vaccine?”) [26% vs. 12%, *P* = 0.028] (Table 1). In the Singapore child group, we found more participants in the intervention group with decreased vaccine confidence regarding safety “vaccines are safe” [29% vs. 10%, *P* = 0.041]. Regarding secondary outcomes, the Fisher’s exact test showed that fewer participants in the Thailand intervention group reported decreased perceived need of COVID-19 vaccines (“my child does not need to be vaccinated” [13% vs. 32%, *P* = 0.006]) and perceived risk of COVID-19 disease (“COVID-19 is a serious disease”) [7.2% vs. 25%, *P* = 0.008] compared to the control group, after the intervention (Supplementary Table 3). Similarly, fewer participants in the Singapore child group reported decreased perceived risk of infection (“My child might get COVID-19”) [8.9% vs. 30%, *P* = 0.011], compared to the control group participants (Supplementary Table 3).

**Table 1:** Vaccine confidence and acceptance for child groups in Thailand, Hong Kong, and Singapore.

	Thailand			Hong Kong			Singapore		
	Control (*n* = 65)	Intervention (*n* = 69)	*P-*value	Control (*n* = 109)	Intervention (*n* = 90)	*P-*value	Control (*n* = 50)	Intervention (*n* = 45)	*P*-value
**Vaccines are important**			0.82			0.41			0.17
Decreased	9 (14%)	7 (10%)		16 (15%)	9 (10%)		3 (6.0%)	8 (18%)	
No change	41 (63%)	45 (65%)		64 (59%)	61 (68%)		36 (72%)	31 (69%)	
Improved	15 (23%)	17 (25%)		29 (27%)	20 (22%)		11 (22%)	6 (13%)	
**Vaccines are safe**			0.27			0.72			**0.041**
Decreased	9 (14%)	7 (10%)		13 (12%)	9 (10%)		5 (10%)	13 (29%)	
No change	34 (52%)	46 (67%)		66 (61%)	60 (67%)		34 (68%)	21 (47%)	
Improved	22 (34%)	16 (23%)		30 (28%)	21 (23%)		11 (22%)	11 (24%)	
**Vaccines are effective**			**0.023**			0.28			NA
Decreased	11 (17%)	3 (4.3%)		17 (16%)	10 (11%)		NA	NA	
No change	32 (49%)	47 (68%)		58 (53%)	58 (64%)		NA	NA	
Improved	22 (34%)	19 (28%)		34 (31%)	22 (24%)		NA	NA	
**Vaccines are effective in reducing severe conditions**			0.065			0.20			0.74
Decreased	12 (18%)	4 (5.8%)		17 (16%)	12 (13%)		6 (12%)	8 (18%)	
No change	36 (55%)	40 (58%)		65 (60%)	64 (71%)		35 (70%)	29 (64%)	
Improved	17 (26%)	25 (36%)		27 (25%)	14 (16%)		9 (18%)	8 (18%)	
**Vaccines are effective in preventing infection**			NA			NA			0.63
Decreased	NA	NA		NA	NA		8 (16%)	11 (24%)	
No change	NA	NA		NA	NA		28 (56%)	22 (49%)	
Improved	NA	NA		NA	NA		14 (28%)	12 (27%)	
**Vaccines are effective regardless of manufacturers**			**<0.001**			NA			NA
Decreased	36 (55%)	10 (14%)		NA	NA		NA	NA	
No change	13 (20%)	40 (58%)		NA	NA		NA	NA	
Improved	16 (25%)	19 (28%)		NA	NA		NA	NA	
**Vaccines are effective against all variants**			**<0.001**			NA			NA
Decreased	31 (48%)	6 (8.7%)		NA	NA		NA	NA	
No change	19 (29%)	44 (64%)		NA	NA		NA	NA	
Improved	15 (23%)	19 (28%)		NA	NA		NA	NA	
**Has your child received a COVID-19 vaccine?**			0.79			0.66			0.43
Decreased	2 (3.1%)	3 (4.3%)		7 (6.4%)	6 (6.7%)		1 (2.0%)	2 (4.4%)	
No change	48 (74%)	53 (77%)		82 (75%)	72 (80%)		42 (84%)	40 (89%)	
Improved	15 (23%)	13 (19%)		20 (18%)	12 (13%)		7 (14%)	3 (6.7%)	
**Has your child received/do you intend for your child to receive a COVID-19 vaccine?**			0.60			**0.028**			0.20
Decreased	3 (4.6%)	6 (8.7%)		13 (12%)	23 (26%)		2 (4.0%)	5 (11%)	
No change	39 (60%)	42 (61%)		57 (52%)	45 (50%)		31 (62%)	31 (69%)	
Improved	23 (35%)	21 (30%)		39 (36%)	22 (24%)		17 (34%)	9 (20%)	

Distributions of changes in the levels of COVID-19 vaccine confidence and acceptance from the pre- and post-intervention questionnaires. Statistical significance tested using Fisher’s exact test, *p* < 0.05 bolded. Values are numbers (percentages) of participants unless stated otherwise.

**Fig. 2:**
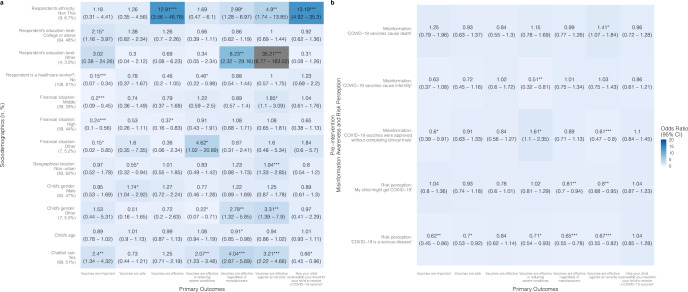
Associations between vaccine confidence and acceptance and sociodemographic factors, misinformation, risk perception, and chatbot use in Thailand child group. A proportional odds logistic regression model adjusted for respondent’s sex, age, and employment status. Reference groups for **a**: respondent’s ethnicity: Thai (number of participants:125, percentage of the total participants: 93%); respondent’s education level: below college level (66, 49%); respondent is a healthcare worker?: yes (17, 13%); financial situation: low (29, 22%); geographical location: urban (51, 38%); child’s gender: female (64, 48%); chatbot use: no (65, 49%). *p-value < 0.05; **p-value < 0.01; ***p-value < 0.001. **b** Refers to the association between pre-intervention misinformation awareness and risk perception with primary outcomes, for example, higher misinformation awareness (“COVID-19 vaccines cause death”) in the pre-intervention questionnaire is positively associated with increase in vaccine confidence (“Vaccines are effective against all variants”) (OR = 1.41 (95% CI: 1.07–1.84)).

In the regression model, chatbot users from the Thailand child group were more likely after the intervention to have increased confidence in vaccines’ importance [OR = 2.40 (95% CI: 1.34–4.32)], effectiveness in reducing severe conditions [OR = 2.07 (1.23–3.48)], effectiveness regardless of manufacturers [OR = 4.04 (2.87–5.69)], and effectiveness against all variants [OR = 3.21 (2.22–4.66)] compared to the control group; however, the intervention group was less likely to have improved vaccine acceptance after the intervention (“has your child received/do you intend for your child to receive a COVID-19 vaccine?”) [OR = 0.66 (0.45–0.96)] compared to the control group (Fig. 2 and Supplementary Table 9).

In the Thailand senior group, a comparison using Fisher’s exact test showed that more participants in the intervention group reported a decrease in vaccine confidence related to effectiveness in reducing severe conditions [12% vs. 21%, *P* = 0.024] and misinformation awareness related to COVID-19 vaccines’ completion of clinical trials [3.4% vs.18%, *P* = 0.026] than the control group (Table 2 and Supplementary Table 4). In the Hong Kong senior group, Fisher’s exact test showed no significant findings on either the primary or secondary outcomes (Supplementary Table 4). Similar results were obtained from sensitivity analyses excluding Filipino and Indonesian subgroups (Supplementary Tables 5–8 and Supplementary Figs. 2–3).

**Table 2:** Vaccine confidence and acceptance for senior groups in Thailand and Hong Kong.

	Thailand			Hong Kong		
	Control (*n* = 73)	Intervention (*n* = 59)	*P*-value	Control (*n* = 106)	Intervention (*n* = 82)	*P-*value
**Vaccines are important**			0.42			0.42
Decreased	12 (16%)	12 (20%)		22 (21%)	11 (13%)	
No change	32 (44%)	30 (51%)		65 (61%)	56 (68%)	
Improved	29 (40%)	17 (29%)		19 (18%)	15 (18%)	
**Vaccines are safe**			0.43			0.37
Decreased	8 (11%)	10 (17%)		16 (15%)	19 (23%)	
No change	39 (53%)	33 (56%)		65 (61%)	46 (56%)	
Improved	26 (36%)	16 (27%)		25 (24%)	17 (21%)	
**Vaccines are effective**			0.46			0.72
Decreased	8 (11%)	11 (19%)		17 (16%)	17 (21%)	
No change	42 (58%)	32 (54%)		60 (57%)	43 (52%)	
Improved	23 (32%)	16 (27%)		29 (27%)	22 (27%)	
**Vaccines are effective in reducing severe conditions**			**0.024**			0.48
Decreased	15 (21%)	7 (12%)		20 (19%)	10 (12%)	
No change	27 (37%)	36 (61%)		63 (59%)	53 (65%)	
Improved	31 (42%)	16 (27%)		23 (22%)	19 (23%)	
**Vaccines are effective regardless of manufacturers**			0.41			NA
Decreased	8 (11%)	7 (12%)		NA	NA	
No change	32 (44%)	32 (54%)		NA	NA	
Improved	33 (45%)	20 (34%)		NA	NA	
**Vaccines are effective against all variants**			0.44			NA
Decreased	12 (16%)	6 (10%)		NA	NA	
No change	36 (49%)	35 (59%)		NA	NA	
Improved	25 (34%)	18 (31%)		NA	NA	
**Has your family member received a COVID-19 vaccine?**			0.19			0.23
Decreased	3 (4.1%)	0 (0%)		5 (4.7%)	1 (1.2%)	
No change	58 (79%)	53 (90%)		92 (87%)	69 (84%)	
Improved	12 (16%)	6 (10%)		9 (8.5%)	12 (15%)	
**Has your family member received/do you intend for your family member to receive a COVID-19 vaccine?**			0.33			0.75
Decreased	6 (8.2%)	2 (3.4%)		6 (5.7%)	4 (4.9%)	
No change	38 (52%)	37 (63%)		79 (75%)	58 (71%)	
Improved	29 (40%)	20 (34%)		21 (20%)	20 (24%)	

Distributions of changes in the levels of COVID-19 vaccine confidence and acceptance from the pre- and post-intervention questionnaires. Statistical significance tested using Fisher’s exact test, *p* < 0.05 bolded. Values are numbers (percentages) of participants unless stated otherwise.

**Fig. 3:**
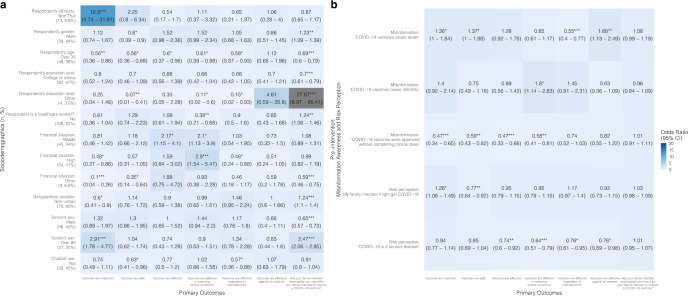
Associations between vaccine confidence and acceptance and sociodemographic factors, misinformation, risk perception, and chatbot use in Thailand senior group. A proportional odds logistic regression model adjusted for respondent’s employment status. Reference groups for **a**: respondent’s ethnicity: Thai (number of participants: 119, percentage of the total participants: 90%); respondent’s sex: female (74, 56%); respondent’s age: 35 and under (84, 64%); respondent’s education level: below college level (66, 50%); respondent is a healthcare worker?: yes (24, 18%); financial situation: low (24, 18%); geographical location: urban (53, 40%); senior’s gender: female (76, 58%); senior’s age: 60–80 (105, 80%); chatbot use: no (73, 55%). *p-value < 0.05; **p-value < 0.01; ***p-value < 0.001. **b** Refers to the association between pre-intervention misinformation awareness and risk perception with primary outcomes, for example, higher misinformation awareness (“COVID-19 vaccines cause death”) in the pre-intervention questionnaire is positively associated with increase in vaccine confidence (“Vaccines are effective against all variants”) (OR = 1.68 (95% CI: 1.13–2.49)).

Chatbot users from the Thailand senior group were less likely to have increased confidence in vaccine safety [OR = 0.63 (0.41–0.96)] and effectiveness regardless of manufacturers [OR = 0.57 (0.36–0.88)] after chatbot use compared to the control group (Fig. 3 and Supplementary Table 9). In the Hong Kong senior group, the intervention group was more likely to show improvements after the intervention in vaccine acceptance [OR = 3.26 (2.53–4.21)], and confidence in vaccine importance [OR = 1.87 (1.12–3.12)] and effectiveness [OR = 1.72 (1.11–2.64)] compared to the control group (Fig. 5 and Supplementary Table 10).

### Factors of vaccine confidence and acceptance

**Fig. 4:**
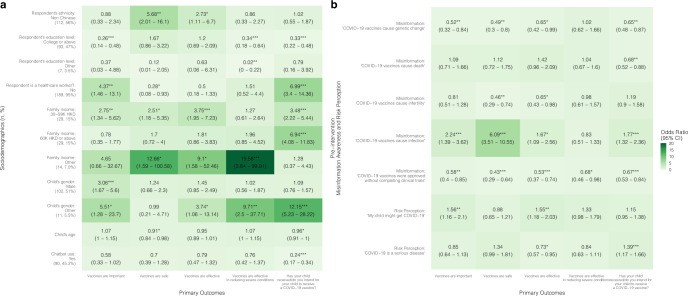
Associations between vaccine confidence and acceptance and sociodemographic factors, misinformation, risk perception, and chatbot use in Hong Kong child group. A proportional odds logistic regression model adjusted for respondent’s sex, age, and employment status. Reference groups for **a**: respondent’s ethnicity: Chinese (number of participants: 87, percentage of the total participants: 44%; respondent’s education level: below college level (99, 50%); respondent is a healthcare worker?: yes (10, 5.0%); family income: under 30 K HKD (127, 64%); child’s gender: female (86, 43%); chatbot use: no (109, 54.8%)). *p-value < 0.05; **p-value < 0.01; ***p-value < 0.001. **b** Refers to the association between pre-intervention misinformation awareness and risk perception with primary outcomes, for example, higher misinformation awareness (“COVID-19 vaccines cause infection”) in the pre-intervention questionnaire is positively associated with increase in vaccine confidence (“Vaccines are important”) (OR = 2.24 (95% CI: 1.39–3.62)).

Chatbots were found to be significantly more effective at improving vaccine confidence and acceptance among people who are minorities (i.e., non-Thai in Thailand and non-Chinese in Hong Kong and Singapore) and those who had lower education levels (i.e., below college level). Specifically, in the child groups, being a minority was associated with higher odds of an improved belief in vaccine effectiveness [OR: Thailand = 12.91 (3.56–46.78); Hong Kong = 2.73 (1.11–6.7)], effectiveness regardless of manufacturers [OR: Thailand = 2.99 (1.28–6.97)], and safety [OR: Hong Kong = 5.68 (2.01–16.1); Singapore = 10.46 (1.14–95.79)] (Figs. 2, 4, 6 and Supplementary Tables 9–11).

**Fig. 5:**
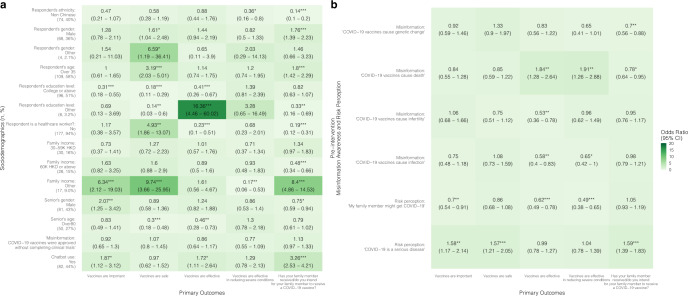
Associations between vaccine confidence and acceptance and sociodemographic factors, misinformation, risk perception, and chatbot use in Hong Kong senior group. A proportional odds logistic regression model adjusted for respondent’s employment status. Reference groups for **a**: respondent’s ethnicity: Chinese (number of participants: 113, percentage of the total participants: 60%); respondent’s sex: female (116, 62%); respondent’s age: 35 and under (78, 42%); respondent’s education level: below college level (86, 46%); respondent is a healthcare worker?: yes (11, 5.9%); family income: under 30 K HKD (113, 60%); senior’s gender: female (107, 57%); senior’s age: 60 to 80 (138, 73%); chatbot use: no (106, 56%). *p-value < 0.05; **p-value < 0.01; ***p-value < 0.001. **b** Refers to the association between pre-intervention misinformation awareness and risk perception with primary outcomes, for example, higher misinformation awareness (“COVID-19 vaccines cause death”) in the pre-intervention questionnaire is positively associated with increase in vaccine confidence (“Vaccines are effective”) (OR = 1.84 (95% CI: 1.28–2.64)).

In the Hong Kong child group, parents with a college education or above were less likely to improve perceptions of vaccine importance [OR = 0.26 (0.14–0.48)], perceptions of vaccine effectiveness in reducing severe conditions [OR = 0.34 (0.18–0.64)], as well as vaccine acceptance [OR = 0.33 (0.22–0.48)], compared to parents with lower education levels in the intervention group (Fig. 4 and Supplementary Table 10). Likewise, in the Hong Kong senior group, respondents with a college or above education level showed lower odds of experiencing improved perceptions of vaccine importance [OR = 0.31 (0.18–0.55)], safety [OR = 0.18 (0.11–0.29)], and effectiveness [OR = 0.41 (0.26–0.67)] (Fig. 5 and Supplementary Table 10).

**Fig. 6:**
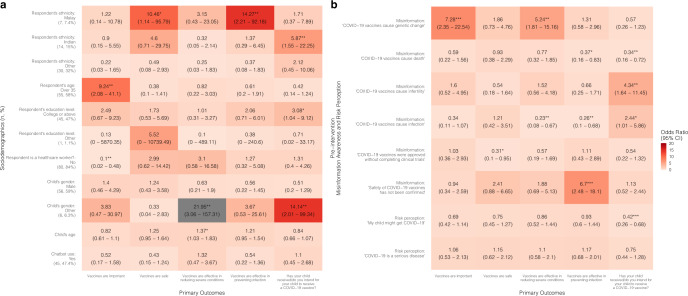
Associations between vaccine confidence and acceptance and sociodemographic factors, misinformation, risk perception, and chatbot use in Singapore child group. A proportional odds logistic regression model adjusted for respondent’s sex and employment status, and housing type. Reference groups for **a**: respondent’s ethnicity: Chinese (number of participants: 44, percentage of the total participants: 46%); respondent’s age: 35 and under (40, 42%); respondent’s education level: below college level (49, 52%); is respondent a healthcare worker?: yes (15, 16%); child’s gender: female (33, 35%); chatbot use: no (50, 52.6%). *p-value < 0.05; **p-value < 0.01; ***p-value < 0.001. **b** Refers to the association between pre-intervention misinformation awareness and risk perception with primary outcomes, for example, higher misinformation awareness (“COVID-19 vaccines cause genetic change”) in the pre-intervention questionnaire is positively associated with increase in vaccine confidence (“Vaccines are important”) (OR = 7.28 (95% CI: 2.35–22.54)).

Respondents who were better at identifying COVID-19 vaccine misinformation in the baseline survey were more likely to have improved vaccine confidence and acceptance. In the Hong Kong child group, parents who correctly identified the misinformation: “COVID-19 vaccines cause infection” in the pre-intervention questionnaire were more likely to have improved beliefs in vaccine importance [OR = 2.24 (1.39–3.62)], safety [OR = 6.09 (3.51–10.55)], effectiveness [OR = 1.67 (1.09–2.56)], and acceptance [OR = 1.77 (1.32–2.36)] (Fig. 4, Supplementary Table 10). In the Singapore child group, parents who correctly identified the misinformation: “COVID-19 vaccines cause genetic change” were more likely to have improved beliefs in vaccine importance [OR = 7.28 (2.35–22.54)] and effectiveness [OR = 5.24 (1.81–15.16)] (Fig. 6 and Supplementary Table 11).

Respondents with higher risk perceptions were less likely to improve in their vaccine confidence and acceptance compared to those with lower risk perceptions (Figs. 2–6 and Supplementary Tables 9–11), meaning that perceived risks might have been the reason for their hesitation but chatbot use was not enough to sway their opinions or reduce their concerns about the vaccine. In the Thailand child group, high risk perception of the severity of COVID-19 disease was negatively associated with increases in beliefs of importance [OR = 0.62 (0.45–0.86)], vaccine safety [OR = 0.7 (0.53–0.92)], effectiveness in reducing severe conditions [OR = 0.71 (0.54–0.93)], effectiveness regardless of manufacturers [OR = 0.65 (0.55–0.78)], and effectiveness regardless of all variants [OR = 0.67 (0.55–0.82)] (Fig. 2 and Supplementary Table 9); in the Singapore child group, higher risk perception of COVID-19 infection was negatively associated with vaccine acceptance [OR = 0.42 (0.26–0.68)] (Fig. 6 and Supplementary Table 11).

### Re-aim framework: evaluation of the intervention

Results from RE-AIM evaluations on the chatbot intervention are presented in the Supplementary Table 13. In brief, Hong Kong and Singapore’s D^2^4H chatbot reached a total of 185 targeted users during the study. Thai ChatSure, which was further along in development compared with the D^2^4H chatbot and had already been publicly accessible, reached 40,613 users since its launch in June 2021 and 1495 users during the study period. Both chatbots were supported by existing studies that demonstrated chatbots’ potential for combating infodemic via prompt information dissemination^29^ and improving vaccination intention through enhanced knowledge and self-efficacy^30^. Our formative data on chatbot users’ adaptions to the intervention suggested considerable interest in the public toward vaccine-related chatbots, as corroborated by the proportion of chatbot users who agreed or strongly agreed with the question: “I intend to use the chatbot again” [Thailand = 87%; Hong Kong = 73%; Singapore = 82%]. The Thai Ministry of Health brought ChatSure to the public specifically to combat COVID-19 vaccine-related misinformation; the D^2^4H chatbot is currently being expanded to cover other vaccines, such as the HPV vaccine, so it may serve as a scalable intervention for existing vaccination campaigns to enhance online engagement with the goal of increasing vaccine confidence.

## Discussion

This study reports the impact of COVID-19 chatbots on vaccine confidence and acceptance of individuals who are unvaccinated or have delayed vaccinations in Thailand, Hong Kong, and Singapore. Most notably, in the Thai child group, we saw greater improvements in the chatbot users’ beliefs regarding vaccine effectiveness and debunking misinformation about COVID-19 vaccines and infertility. However, we also observed differentiating results based on the target population and region including backfire effects of chatbot use on both vaccine acceptance and confidence. The impact of chatbots was more effective in improving vaccine confidence and acceptance among unsure minorities and people with lower level of education, but less so for the highly educated population.

This study is a multisite, parallel RCT on multilingual vaccine chatbots. It was conducted in three Asian regions, one being upper-middle-income and two being high-income. As this study was conducted during the aggressive implementation of containment interventions such as social distancing rules and mandatory vaccine pass schemes by the governments in our study sites, we employed the RCT design to evaluate the impact of the chatbot intervention. Chatbot development and evaluation were constantly updated and tailored to changing local epidemic situations and vaccine policies and programmes (e.g., approval of the 5–11 age group vaccinations)^22^ to disseminate accurate information. Chatbots accommodated the most used languages (i.e., Thai in Thailand; English, Simplified, and Traditional Chinese in Hong Kong and Singapore) and one of the most widely used communication platforms in their respective regions (i.e., WhatsApp Messenger in Hong Kong and Singapore), and were developed by leading public health research organisations or government agencies in the study sites. The questionnaires were standardised across countries and contexts to compare outcome variables of interest. The chatbots’ high practicality, flexibility (i.e., the ability to adapt to different settings, such as HPV vaccination campaigns), and scalability demonstrated promising evidence for future research and applications.

Nevertheless, our study has several limitations. First, our sample sizes across all three regions were smaller than our target sample sizes. A combination of heightened risk perception owing to increased daily case counts and public health measures, such as school-based vaccine rollout and vaccine mandates, led to surges in vaccination uptake in our study locations, leaving only a small population who remained unvaccinated during our study period. Although we have found some statistically significant findings, our small sample sizes might lack sufficient power to detect the effect of the intervention in its entirety, e.g., dose-dependent effect (Supplementary Table 14). In order to recruit more participants, we relaxed our study criteria to include those who delayed their vaccination until the implementation of governmental vaccine mandates. Another possible reason for our small sample size is the high proportions of participants lost to follow-up, potentially due to our chatbot’s design^31^. For instance, our chatbot was not able to recognize users’ emotions and tailor phrase responses to questions. In addition, since participants recruited by Premise were more familiar with surveys related to market research rather than vaccines, their indifference to domains of chatbot contents might have led to user dissatisfaction and consequently a high drop-out rate. Second, our sample population was not representative of the populations in respective regions. To address this limitation, we applied population weightings in regression models based on respective regions’ census data to adjust for potential biases. Third, the chatbot employed in Hong Kong and Singapore only had COVID-19 vaccine-related content and was unable to answer general COVID-19 questions (i.e., COVID-19 home care instructions, daily COVID-19 cases). As a result, participants might have engaged less with the chatbot and rated the chatbots as less helpful than they would have otherwise. Fourth, our study might have social desirability bias since outcomes are self-reported amid active governmental encouragement and mandates on vaccination during the Omicron outbreak. Fifth, our study design incorporated responses of guardians to gauge vaccine confidence and acceptance of unvaccinated seniors due to lacking eligible senior participants in the existing panel. It is inferred that changes in the guardians’ vaccine confidence and acceptance will affect those of unvaccinated seniors, as family support is one of the most influential factors in senior vaccine hesitancy in this region^32–34^. However, this proxy approach might not directly reflect the changes in unvaccinated seniors’ vaccine confidence and acceptance. Further studies could advance the generalizability of chatbot interventions to target seniors, instead of their guardians, directly, and also investigate whether improve confidence in vaccine effectiveness could be translated into vaccination actions. Finally, our study focused on vaccine confidence and acceptance among numerous other factors that could drive vaccine hesitancy and deter children and seniors’ COVID-19 vaccine uptake. Studies suggest that differences in sociodemographic structures, health literacy, prevalence of chronic diseases, distributions of vaccine supplies, convenience to vaccinations, trust towards healthcare systems, governments, and vaccine developers impact vaccination coverages^35–40^. While vaccine confidence and acceptance are important attributes of vaccine uptake, our findings are not to be interpreted as the sole indicator of vaccination behaviours.

The first major finding of this study is that there was an increase in vaccine acceptance and confidence among some chatbot users across different study groups, adding to a growing body of evidence that well-designed and implemented chatbots can have a positive influence on health behaviours^19,27,41–47^. While there has been growing interest in chatbots across a range of public health areas^19,27,41–43,48^, very few studies have previously investigated the effectiveness of chatbots in promoting vaccine acceptance using RCTs^43,49–51^. For COVID-19 vaccination, our study lends weight to previous findings that interactive conversations between chatbots and users can contribute to increased vaccine confidence, as seen in the Thailand child group^27,52^. Additionally, since our chatbots were hosted on two major mobile messenger apps, WhatsApp and Facebook Messenger, our study also adds weight to previous evidence supporting the potential of mobile messenger applications in delivering vaccination knowledge, debunking vaccine-related misinformation, and providing vaccination guidance to unvaccinated individuals^53,54^. Our data showed that a majority of chatbot users were less likely to decrease in vaccine confidence and acceptance, compared with non-users; however, our study also found some apparent evidence of ‘backfire’ effects, specifically among some chatbot using parents in Hong Kong where we observed decreased vaccine acceptance with chatbot use. ‘Backfire effects’ are a controversial topic within the literature on digital health interventions—some research suggests that, in certain circumstances, pro-vaccine messaging delivered through social media can be counterproductive^29^. This might occur if, for example, messaging runs counter to the values of the target group or conflicts with individuals’ personal experiences—in our case, we noted chatbots worked better for improving vaccine confidence and uptake among minority subpopulations and users with lower level of education, but were less effective in swaying the mainstream population with higher education levels. Some studies have also raised concerns that repeating misinformation in order to correct or debunk it can have the perverse effect of increasing people’s familiarity with the misinformation, or may even spread misinformation to new audiences who had never been exposed to it before^55^. However, other studies have failed to replicate these findings^56^. In the case of our study, it is unclear why certain groups should have seen adverse outcomes on certain variables. Further in-depth investigations are needed. Conceivably, there may have been specific safety concerns or misinformation narratives that some had been less aware of prior to the study, and the process of engaging with the chatbot may have increased their familiarity with these topics or narratives.

Future chatbots may improve on their vaccine promotion and communication strategies as well as their message delivery, such as by using an emotion-based approach that can convey reassurances to chatbot users to ameliorate their doubts and fears^28,57^. An anthropomorphic chatbot that can share anecdotes may also have a positive impact; Loft et al. found that personal stories that go beyond facts and traditional sources of authority can be more persuasive in online communications campaigns^30^. Further advancement in AI technology, Natural Language Processing, and machine learning is immediately needed as the current chatbot operation relies heavily on human analysis to ensure response accuracy, especially in free text conversations. Further, chatbots should be supervised by trusted experts to ensure not only information accuracy, but data security and ethics compliance. Nevertheless, chatbots can be a useful component of a multi-pronged approach to health service delivery and communication, for example in combination with a webinar series or website with interactive features^29,58,59^. A more standardized assessment should be conducted to better analyse and improve chatbot’s effectiveness in handling users’ questions and influencing behaviours.

These study target populations who are unvaccinated or have delayed vaccination to identify viable strategies that could be applied in ongoing endeavours towards vaccine hesitancy alleviation^22,23,60–62^. The findings of this study present that, while chatbots are a potentially beneficial intervention that could provide insights to policymakers on the nature of vaccine concerns and inform strategies that can better address vaccine hesitancy, evidence on the effectiveness of chatbots on vaccine confidence and uptake is not conclusive across different populations and requires further assessment. We suggest interventions be interpreted and modified to address idiosyncratic local contexts in order to reach optimal results. Concurrently, it is important that future investigations on chatbot interventions to enhance vaccine confidence include underrepresented minority groups, such as migrant workers, to broaden the applicability and scalability of chatbots.

## Methods

### Ethics and informed consent

This study was approved by the Institutional Review Board of The University of Hong Kong/ Hospital Authority Hong Kong West Cluster (UW 21–659), National University of Singapore, Saw Swee Hock School of Public Health Departmental Ethics Review Committee (SSHSPH-158), and the Ethics Committee of the Institute for the Development of Human Research Protections of the Ministry of Public Health, Thailand (IHRP 1122–2654). The study protocol was registered at ClinicalTrials.gov (ID: NCT05424952) and made publicly available on July 22, 2022. All study participants electronically signed the consent form.

### Study design

We conducted a multisite, parallel, randomised controlled trial (RCT) designed to evaluate the effectiveness of COVID-19 chatbots for improving COVID-19 vaccine confidence and acceptance in three Asian locations concurrently: Thailand, Hong Kong, and Singapore. Randomisation and group allocation were performed by a participant recruitment and market research company, Premise. We ran a pilot study from January 17th to February 11th and validated the survey tools using confirmatory factor analysis (Supplementary Method 1)^63–65^. From February 11th to June 30th, 2022, eligible users in the online panel within Premise’s mobile application were randomly invited to join the control or intervention group in our study with an allocation ratio of 1:1. The study was double-blinded—both participants and outcomes assessors were concealed from the intervention assignment. After signing the consent form, participants were asked to complete the pre- and post-intervention questionnaires and to use the chatbot if assigned to the intervention group. The formative, impact, and process assessment of the chatbot intervention was conducted following the RE-AIM framework^66,67^, and was quantitatively assessed based on its *reach, efficacy, adoption, implementation,* and *maintenance*.

### Study population

Our study population included guardians of those who were unvaccinated or delayed their COVID-19 vaccinations until the government vaccine mandates (Supplementary Method 2 and Supplementary Fig. 1). Children and seniors had the lowest vaccination coverages in all study regions despite their COVID-19 disease vulnerability. Since guardians can make direct or indirect vaccination decision on behalf of children and seniors, we tested the effectiveness of chatbot in increasing guardians’ vaccine confidence and acceptance for their dependent family members. Taking an average of estimates from similar studies conducted in Japan and France^53,68^, we estimated an effect size of 15% and determined a sample size of 250 for each of the control and intervention group using power analysis. However, our sample sizes did not reach the target due to the surge in vaccine uptake at the time of participant recruitment following the emergence of Omicron and subsequent governmental vaccine mandates, school-based vaccine rollouts and increased risk perceptions of COVID-19 (Supplementary Fig. 1). In Thailand, the eligibility criteria included (1) adults with unvaccinated parents/grandparents aged 60 years or above, or (2) parents of unvaccinated children aged 5–11 years. The eligibility criteria in Hong Kong were slightly relaxed to include (1) adults whose parents/grandparents were 60 years old or above and had not been vaccinated against COVID-19 before the announcement of the mandatory Vaccine Pass on January 4, 2022^69^, or (2) parents of unvaccinated children under 18 years old. In Singapore, the eligibility criteria included parents of unvaccinated children aged 5–11 years (Supplementary Method 2). In all three locations, participants were recruited by Premise, a participant recruitment and market research company^70^, via random sampling using existing online panels.

### Intervention

In Thailand, we adopted and updated ChatSure (Supplementary Method 3), an extant COVID-19 chatbot on Facebook Messenger, developed by the Ministry of Public Health, the Thai Health Promotion Foundation, Facebook Thailand, Hbot, the International Health Policy Program, and the National Vaccine Institute^71^. For Hong Kong and Singapore, we designed D^2^4H vaccine chatbot and implemented it on the WhatsApp platform (Supplementary Method 4). Both free text mode and FAQ-style browsing mode were employed in the chatbots to improve vaccine confidence and acceptance by promptly providing accurate and consolidated vaccine-related information. Questions and comments from the participants are presented in supplementary information as word clouds (Supplementary Figs. 4–7).

The COVID-19 vaccine chatbot content covered seven major categories of commonly asked questions: (1) Importance/Necessity, (2) Safety, (3) Effectiveness, (4) How to get vaccinated, (5) Tips before vaccination, (6) Tips after vaccination, and (7) Debunking COVID-19 vaccine-related misinformation. The information and dialogues were tailored and timely modified to the local contexts and regulations. Participants were allowed to enquire using English or the mainstream local languages (i.e., *Thai* in Thailand; *Traditional Chinese, Simplified Chinese* and *English* in Hong Kong*;* and *Simplified Chinese* and *English* in Singapore).

For both the intervention and control groups, pre-intervention surveys were sent to participants to determine their demographics. All participants were then asked to answer questions regarding COVID-19 vaccine confidence, including perceived importance, effectiveness, and safety, vaccine acceptance, and COVID-19 vaccine-related misinformation. Study instruments are available in Supplementary Methods 5–7.

Next, participants assigned to the intervention group were directed to use the chatbot for up to 1 week through an invite-only link after completing the pre-intervention questionnaire. Participants in the intervention group were prompted by the chatbot to exchange at least ten messages regarding COVID-19 vaccines with the chatbot and submit screenshots of chatbot dialogues for validation before receiving a small reward for participation (<USD$15.00; Supplementary Table 12). Participants assigned to the control group were not given access to COVID-19 vaccine-related chatbots. After the intervention period, participants from both groups were asked to complete the same questionnaire about COVID-19 vaccines; chatbot users were asked to evaluate their usage experience.

### Outcomes

The primary outcomes were changes in the levels of COVID-19 vaccine confidence and acceptance from the pre- and post-intervention questionnaires. Confidence was measured using the Vaccine Confidence Index ^TM^ (VCI)^72^, which has been used in the context of COVID-19 vaccinations^73,74^ and included perceptions of the *importance, effectiveness*, and *safety* of COVID-19 vaccines. VCI was recorded on a 5-point Likert scale (ranging from 5-strongly agree, 4-agree, 3-neither agree nor disagree, 2-disagree, to 1-strongly disagree) and the pre- and post-intervention differences were categorized into a 3-point scale of “improved”, “no change”, and “declined”. For example, if a participant answered “Agree” in the pre-intervention questionnaire and “Strongly Agree” in the post-intervention questionnaire, the participant would score 1-point. Positive, zero, or negative differences were categorized into “improved”, “no change”, or “decreased” outcomes, respectively. Similarly, vaccine acceptance was measured by the change in participants’ expressed willingness to vaccinate their senior parents/grandparents or children from the pre- and post-intervention questionnaires; the difference in pre- and post-intervention responses was categorized into the same 3-point scale. Secondary outcomes included differences in perceived risks and benefits of COVID-19 vaccines and participants’ awareness and knowledge of COVID-19 vaccine-related misinformation before and after the intervention. For the chatbot evaluation, outcomes were described based on the RE-AIM framework criteria (Supplementary Table 13), and conversation contents of D^2^4H chatbot and ChatSure in local languages were presented in word clouds (Supplementary Figs. 4–7).

### Data analysis

All participants who completed the assigned questionnaires and the intervention were analysed per protocol. The changes in outcome variables were measured on a 3-point scale (*“improved”, “no change”, and “decreased”*) and compared by both Fisher’s exact test and a proportional odds logistic regression model between the control and intervention groups to examine the effect of chatbot use on primary outcomes variables. We further employed proportional odds logistic regressions to investigate factors of primary outcome measures—vaccine confidence and acceptance where all participants’ data were weighted with sex and ethnicity using the latest local census data^48,75,76^.

### Reporting summary

Further information on research design is available in the Nature Research Reporting Summary linked to this article.

## Supplementary information

Supplementary Information

Reporting Summary

## Data Availability

Anonymized data and code used can be found at: https://github.com/lkwok/VCF_chatbot.

## Code availability

All codes used for the analyses and visualizations can be found at: https://github.com/lkwok/VCF_chatbot.
