# Supplementary material for: Effectiveness of chatbots on COVID vaccine confidence and acceptance in Thailand, Hong Kong, and Singapore
Source: NPJ Digit Med. 2023 May 25;6:96. doi: 10.1038/s41746-023-00843-6 (PMC10208906; doi:10.1038/s41746-023-00843-6)
Supplement: Supplementary file 1 — Supplementary Information [file 41746_2023_843_MOESM1_ESM.docx]

**Effectiveness of chatbots on COVID vaccine confidence and acceptance in Thailand, Hong Kong, and Singapore**

Kristi Yoonsup Lee, Saudamini Vishwanath Dabak, Vivian Hanxiao Kong, Minah Park, Shirley L. L. Kwok, Madison Silzle, Chayapat Rachatan, Alex Cook, Aly Passanante, Ed Pertwee, Zhengdong Wu, Javier A. Elkin, Heidi J. Larson, Eric H.Y. Lau, Kathy Leung, Joseph T. Wu, Leesa Lin

**Supplementary Information**

**Supplementary Table 1. Baseline characteristics of control and intervention groups for the senior group in Thailand and Hong Kong.**

|  | **Thailand** | | | | **Hong Kong** | | | |
| --- | --- | --- | --- | --- | --- | --- | --- | --- |
|  | **Control (n = 73)** | **Intervention (n = 59)** | **Overall (n = 132)** | **P Value** | **Control (n = 106)** | **Intervention (n = 82)** | **Overall (n = 188)** | **P Value** |
| **Respondent’s ethnicity** |  |  |  | 0·04 |  |  |  | 0·39 |
| Chinese | 0 (0%) | 0 (0%) | 0 (0%) |  | 69 (66%) | 44 (54%) | 113 (60%) |  |
| Filipino | 0 (0%) | 0 (0%) | 0 (0%) |  | 25 (24%) | 24 (29%) | 49 (26%) |  |
| Indonesian | 0 (0%) | 0 (0%) | 0 (0%) |  | 6 (5·7%) | 7 (8·5%) | 13 (7·0%) |  |
| Thai | 62 (85%) | 57 (97%) | 119 (90%) |  | 0 (0%) | 0 (0%) | 0 (0%) |  |
| Other/Prefer not to answer | 11 (15%) | 2 (3·4%) | 13 (9·8%) |  | 5 (4·8%) | 7 (8·5%) | 12 (6·4%) |  |
| Missing | 0 | 0 | 0 |  | 1 | 0 | 1 |  |
| **Respondent’s gender** |  |  |  | 0·11 |  |  |  | 0·36 |
| Female | 36 (49%) | 38 (64%) | 74 (56%) |  | 63 (59%) | 53 (65%) | 116 (62%) |  |
| Male | 37 (51%) | 21 (36%) | 58 (44%) |  | 42 (40%) | 26 (32%) | 68 (36%) |  |
| Other | 0 (0%) | 0 (0%) | 0 (0%) |  | 1 (0·9%) | 3 (3·7%) | 4 (2·1%) |  |
| **Respondent’s age** |  |  |  | 0·27 |  |  |  | 0·37 |
| 35 and under | 43 (59%) | 41 (69%) | 84 (64%) |  | 41 (39%) | 37 (46%) | 78 (42%) |  |
| Over 35 | 30 (41%) | 18 (31%) | 48 (36%) |  | 65 (61%) | 44 (54%) | 109 (58%) |  |
| Missing | 0 | 0 | 0 |  | 0 | 1 | 1 |  |
| **Respondent’s education level** |  |  |  | 0·86 |  |  |  | 0·18 |
| Below college level | 35 (48%) | 31 (53%) | 66 (50%) |  | 54 (51%) | 32 (39%) | 86 (46%) |  |
| College or above | 34 (47%) | 28 (47%) | 62 (47%) |  | 50 (47%) | 46 (56%) | 96 (51%) |  |
| Other | 4 (5·5%) | 0 (0%) | 4 (3·0%) |  | 2 (1·9%) | 4 (4·9%) | 6 (3·2%) |  |
| **Respondent’s employment status** |  |  |  | 0·17 |  |  |  | 0·14 |
| Unemployed/Economically inactive | 22 (31%) | 12 (20%) | 34 (26%) |  | 26 (25%) | 28 (36%) | 54 (30%) |  |
| Employed | 48 (69%) | 47 (80%) | 95 (74%) |  | 77 (75%) | 49 (64%) | 126 (70%) |  |
| Missing | 3 | 0 | 3 |  | 3 | 5 | 8 |  |
| **Is the respondent working in a healthcare setting?** |  |  |  | 0·012 |  |  |  | 0·12 |
| Yes | 19 (26%) | 5 (8·5%) | 24 (18%) |  | 9 (8·5%) | 2 (2·4%) | 11 (5·9%) |  |
| No | 54 (74%) | 54 (92%) | 108 (82%) |  | 97 (92%) | 80 (98%) | 177 (94%) |  |
| **Family income** |  |  |  | NA |  |  |  | 0·95 |
| Under 30K HKD | NA | NA | NA |  | 64 (60%) | 49 (60%) | 113 (60%) |  |
| 30-59K HKD | NA | NA | NA |  | 16 (15%) | 14 (17%) | 30 (16%) |  |
| 60K HKD or above | NA | NA | NA |  | 16 (15%) | 12 (15%) | 28 (15%) |  |
| Other | NA | NA | NA |  | 10 (9·4%) | 7 (8·5%) | 17 (9·0%) |  |
| **Financial situation** |  |  |  | 1 |  |  |  | NA |
| Low | 13 (18%) | 11 (19%) | 24 (18%) |  | NA | NA | NA |  |
| Middle | 25 (34%) | 20 (34%) | 45 (34%) |  | NA | NA | NA |  |
| High | 30 (41%) | 24 (41%) | 54 (41%) |  | NA | NA | NA |  |
| Other | 5 (6·8%) | 4 (6·8%) | 9 (6·8%) |  | NA | NA | NA |  |
| **Geographical location** |  |  |  | 0·48 |  |  |  | NA |
| Urban | 27 (37%) | 26 (44%) | 53 (40%) |  | NA | NA | NA |  |
| Non-urban | 46 (63%) | 33 (56%) | 79 (60%) |  | NA | NA | NA |  |
| **Senior’s gender** |  |  |  | 0·49 |  |  |  | 0·77 |
| Female | 40 (55%) | 36 (61%) | 76 (58%) |  | 59 (56%) | 48 (59%) | 107 (57%) |  |
| Male | 33 (45%) | 23 (39%) | 56 (42%) |  | 47 (44%) | 34 (41%) | 81 (43%) |  |
| **Senior’s age** |  |  |  | 0·67 |  |  |  | 0·51 |
| 60 to 80 | 57 (78%) | 48 (81%) | 105 (80%) |  | 80 (75%) | 58 (71%) | 138 (73%) |  |
| over 80 | 16 (22%) | 11 (19%) | 27 (20%) |  | 26 (25%) | 24 (29%) | 50 (27%) |  |

Statistical significance tested using Fisher’s exact test, p <0.05 bolded. Values are numbers (percentages) of participants unless stated otherwise.

**Supplementary Table 2. Baseline characteristics of control and intervention groups for the child groups in Thailand, Hong Kong, and Singapore.**

|  | Thailand | | | | Hong Kong | | | | Singapore | | | |
| --- | --- | --- | --- | --- | --- | --- | --- | --- | --- | --- | --- | --- |
|  | **Control (n = 65)** | **Intervention (n = 69)** | **Overall (n = 134)** | **P Value** | **Control (n = 109)** | **Intervention (n = 90)** | **Overall (n = 199)** | **P Value** | **Control (n = 50)** | **Intervention (n = 45)** | **Overall (n = 95)** | **P Value** |
| Respondent’s ethnicity |  |  |  | 0·17 |  |  |  |  |  |  |  | 0·16 |
| Chinese | 0 (0%) | 0 (0%) | 0 (0%) |  | 41 (38%) | 46 (51%) | 87 (44%) | 0·18 | 21 (42%) | 23 (51%) | 44 (46%) |  |
| Indian | 0 (0%) | 0 (0%) | 0 (0%) |  | 0 (0%) | 0 (0%) | 0 (0%) |  | 2 (4·0%) | 5 (11%) | 7 (7·4%) |  |
| Malay | 0 (0%) | 0 (0%) | 0 (0%) |  | 0 (0%) | 0 (0%) | 0 (0%) |  | 9 (18%) | 5 (11%) | 14 (15%) |  |
| Filipino | 0 (0%) | 0 (0%) | 0 (0%) |  | 52 (48%) | 33 (37%) | 85 (43%) |  | 16 (32%) | 7 (16%) | 23 (24%) |  |
| Indonesian | 0 (0%) | 0 (0%) | 0 (0%) |  | 10 (9·2%) | 8 (8·9%) | 18 (9·0%) |  | 0 (0%) | 0 (0%) | 0 (0%) |  |
| Thai | 63 (97%) | 62 (90%) | 125 (93%) |  | 0 (0%) | 0 (0%) | 0 (0%) |  | 0 (0%) | 0 (0%) | 0 (0%) |  |
| Other/Prefer not to answer | 2 (3·1%) | 7 (10%) | 9 (6·7%) |  | 6 (5·5%) | 3 (3·3%) | 9 (4·5%) |  | 2 (4·0%) | 5 (11%) | 7 (7·4%) |  |
| Respondent’s gender |  |  |  | 0·29 |  |  |  | 0·11 |  |  |  | 0·84 |
| Female | 44 (68%) | 40 (58%) | 84 (63%) |  | 84 (77%) | 60 (67%) | 144 (72%) |  | 24 (48%) | 21 (47%) | 45 (47%) |  |
| Male | 21 (32%) | 29 (42%) | 50 (37%) |  | 24 (22%) | 29 (32%) | 53 (27%) |  | 25 (50%) | 24 (53%) | 49 (52%) |  |
| Other/Prefer not to answer | 0 (0%) | 0 (0%) | 0 (0%) |  | 1 (0·9%) | 1 (1·1%) | 2 (1·0%) |  | 1 (2·0%) | 0 (0%) | 1 (1·1%) |  |
| Respondent’s age |  |  |  | 0·1 |  |  |  | 0·67 |  |  |  | 0·99 |
| 35 and under | 49 (75%) | 42 (61%) | 91 (68%) |  | 56 (51%) | 49 (55%) | 105 (53%) |  | 21 (42%) | 19 (42%) | 40 (42%) |  |
| Over 35 years old | 16 (25%) | 27 (39%) | 43 (32%) |  | 53 (49%) | 40 (45%) | 93 (47%) |  | 29 (58%) | 26 (58%) | 55 (58%) |  |
| Missing | 0 | 0 | 0 |  | 0 | 1 | 1 |  | 0 | 0 | 0 |  |
| Respondent’s education level |  |  |  | 0·86 |  |  |  | 0·002 |  |  |  | 0·15 |
| Below college level | 32 (49%) | 34 (49%) | 66 (49%) |  | 65 (60%) | 34 (38%) | 99 (50%) |  | 22 (44%) | 27 (60%) | 49 (52%) |  |
| College level or above | 30 (46%) | 34 (49%) | 64 (48%) |  | 40 (37%) | 53 (59%) | 93 (47%) |  | 27 (54%) | 18 (40%) | 45 (47%) |  |
| Other/Prefer not to answer | 3 (4·6%) | 1 (1·4%) | 4 (3·0%) |  | 4 (3·7%) | 3 (3·3%) | 7 (3·5%) |  | 1 (2·0%) | 0 (0%) | 1 (1·1%) |  |
| Respondent’s employment status |  |  |  | 0·12 |  |  |  | 0·003 |  |  |  | 1 |
| Unemployed/Economically inactive | 16 (25%) | 9 (13%) | 25 (19%) |  | 48 (46%) | 22 (25%) | 70 (36%) |  | 8 (16%) | 7 (16%) | 15 (16%) |  |
| Employed | 49 (75%) | 59 (87%) | 108 (81%) |  | 56 (54%) | 67 (75%) | 123 (64%) |  | 42 (84%) | 38 (84%) | 80 (84%) |  |
| Missing | 0 | 1 | 1 |  | 5 | 1 | 6 |  | 0 | 0 | 0 |  |
| Is the respondent working in a healthcare setting? |  |  |  | 0·2 |  |  |  | 0·52 |  |  |  | 0·27 |
| Yes | 11 (17%) | 6 (8·7%) | 17 (13%) |  | 7 (6·4%) | 3 (3·3%) | 10 (5·0%) |  | 10 (20%) | 5 (11%) | 15 (16%) |  |
| No | 54 (83%) | 63 (91%) | 117 (87%) |  | 102 (94%) | 87 (97%) | 189 (95%) |  | 40 (80%) | 40 (89%) | 80 (84%) |  |
| Family income |  |  |  | NA |  |  |  | 0·002 |  |  |  | NA |
| Under 30K HKD | NA | NA | NA |  | 79 (72%) | 48 (53%) | 127 (64%) |  | NA | NA | NA |  |
| 30-59K HKD | NA | NA | NA |  | 13 (12%) | 16 (18%) | 29 (15%) |  | NA | NA | NA |  |
| 60K HKD or above | NA | NA | NA |  | 8 (7·3%) | 21 (23%) | 29 (15%) |  | NA | NA | NA |  |
| Other/Prefer not to answer | NA | NA | NA |  | 9 (8·3%) | 5 (5·6%) | 14 (7·0%) |  | NA | NA | NA |  |
| Housing |  |  |  | NA |  |  |  | NA |  |  |  | 1 |
| HDB | NA | NA | NA |  | NA | NA | NA |  | 34 (68%) | 31 (69%) | 65 (68%) |  |
| Non-HDB | NA | NA | NA |  | NA | NA | NA |  | 16 (32%) | 14 (31%) | 30 (32%) |  |
| Financial situation |  |  |  | 0·47 |  |  |  | NA |  |  |  | NA |
| Low | 17 (26%) | 12 (17%) | 29 (22%) |  | NA | NA | NA |  | NA | NA | NA |  |
| Middle | 17 (26%) | 22 (32%) | 39 (29%) |  | NA | NA | NA |  | NA | NA | NA |  |
| High | 28 (43%) | 31 (45%) | 59 (44%) |  | NA | NA | NA |  | NA | NA | NA |  |
| Other/Prefer not to answer | 3 (4·6%) | 4 (5·8%) | 7 (5·2%) |  | NA | NA | NA |  | NA | NA | NA |  |
| Geographical location |  |  |  | 0·86 |  |  |  | NA |  |  |  | NA |
| Urban | 24 (37%) | 27 (39%) | 51 (38%) |  | NA | NA | NA |  | NA | NA | NA |  |
| Non-urban | 41 (63%) | 42 (61%) | 83 (62%) |  | NA | NA | NA |  | NA | NA | NA |  |
| Child’s gender |  |  |  | 0·16 |  |  |  | 0·38 |  |  |  | 0·51 |
| Female | 34 (52%) | 30 (43%) | 64 (48%) |  | 42 (39%) | 44 (49%) | 86 (43%) |  | 15 (30%) | 18 (40%) | 33 (35%) |  |
| Male | 25 (38%) | 38 (55%) | 63 (47%) |  | 57 (52%) | 45 (50%) | 102 (51%) |  | 30 (60%) | 26 (58%) | 56 (59%) |  |
| Other/Prefer not to answer | 6 (9·2%) | 1 (1·4%) | 7 (5·2%) |  | 10 (9·2%) | 1 (1·1%) | 11 (5·5%) |  | 5 (10%) | 1 (2·2%) | 6 (6·3%) |  |

Statistical significance tested using Fisher’s exact test, p <0.05 bolded. Values are numbers (percentages) of participants unless stated otherwise.

**Supplementary Table 3. Secondary outcome variables for child groups in Thailand, Hong Kong, and Singapore.**

|  | **Thailand** | | | **Hong Kong** | | | **Singapore** | | |
| --- | --- | --- | --- | --- | --- | --- | --- | --- | --- |
|  | **Control**  **(n = 65)** | **Intervention**  **(n = 69)** | **P Value** | **Control**  **(n = 109)** | **Intervention**  **(n = 90)** | **P Value** | **Control**  **(n = 50)** | **Intervention**  **(n = 45)** | **P Value** |
| **My child does not need to be vaccinated** |  |  | **0.006** |  |  | >0.99 |  |  | 0.63 |
| Decreased | 21 (32%) | 9 (13%) |  | 17 (16%) | 14 (16%) |  | 7 (14%) | 6 (13%) |  |
| No change | 22 (34%) | 40 (58%) |  | 57 (52%) | 48 (53%) |  | 23 (46%) | 25 (56%) |  |
| Improved | 22 (34%) | 20 (29%) |  | 35 (32%) | 28 (31%) |  | 20 (40%) | 14 (31%) |  |
| **My child might get COVID-19** |  |  | 0.34 |  |  | 0.62 |  |  | **0.011** |
| Decreased | 17 (26%) | 12 (17%) |  | 25 (23%) | 20 (22%) |  | 15 (30%) | 4 (8.9%) |  |
| No change | 24 (37%) | 33 (48%) |  | 50 (46%) | 47 (52%) |  | 24 (48%) | 21 (47%) |  |
| Improved | 24 (37%) | 24 (35%) |  | 34 (31%) | 23 (26%) |  | 11 (22%) | 20 (44%) |  |
| **COVID-19 is a serious disease** |  |  | **0.008** |  |  | 0.89 |  |  | 0.96 |
| Decreased | 16 (25%) | 5 (7.2%) |  | 17 (16%) | 14 (16%) |  | 8 (16%) | 7 (16%) |  |
| No change | 29 (45%) | 46 (67%) |  | 71 (65%) | 61 (68%) |  | 27 (54%) | 23 (51%) |  |
| Improved | 20 (31%) | 18 (26%) |  | 21 (19%) | 15 (17%) |  | 15 (30%) | 15 (33%) |  |
| **I will be less anxious if my child is vaccinated** |  |  | 0.59 |  |  | 0.64 |  |  | 0.74 |
| Decreased | 8 (12%) | 5 (7.2%) |  | 17 (16%) | 11 (12%) |  | 11 (22%) | 8 (18%) |  |
| No change | 33 (51%) | 35 (51%) |  | 62 (57%) | 57 (63%) |  | 28 (56%) | 29 (64%) |  |
| Improved | 24 (37%) | 29 (42%) |  | 30 (28%) | 22 (24%) |  | 11 (22%) | 8 (18%) |  |
| **Vaccination of children can control the spread of COVID-19** |  |  | 0.87 |  |  | 0.36 |  |  | >0.99 |
| Decreased | 12 (18%) | 10 (14%) |  | 20 (18%) | 10 (11%) |  | 9 (18%) | 8 (18%) |  |
| No change | 34 (52%) | 38 (55%) |  | 65 (60%) | 57 (63%) |  | 27 (54%) | 25 (56%) |  |
| Improved | 19 (29%) | 21 (30%) |  | 24 (22%) | 23 (26%) |  | 14 (28%) | 12 (27%) |  |
| **My child will get vaccinated if many others are vaccinated** |  |  | 0.59 |  |  | 0.18 |  |  | >0.99 |
| Decreased | 9 (14%) | 6 (8.7%) |  | 33 (30%) | 17 (19%) |  | 9 (18%) | 8 (18%) |  |
| No change | 37 (57%) | 39 (57%) |  | 54 (50%) | 54 (60%) |  | 26 (52%) | 23 (51%) |  |
| Improved | 19 (29%) | 24 (35%) |  | 22 (20%) | 19 (21%) |  | 15 (30%) | 14 (31%) |  |
| **My child will get vaccinated if there is a vaccine mandate** |  |  | 0.19 |  |  | 0.44 |  |  | 0.66 |
| Decreased | 10 (15%) | 4 (5.8%) |  | 21 (19%) | 16 (18%) |  | 9 (18%) | 5 (11%) |  |
| No change | 33 (51%) | 42 (61%) |  | 63 (58%) | 46 (51%) |  | 28 (56%) | 26 (58%) |  |
| Improved | 22 (34%) | 23 (33%) |  | 25 (23%) | 28 (31%) |  | 13 (26%) | 14 (31%) |  |
| **I think COVID-19 vaccination should be mandatory** |  |  | 0.56 |  |  | 0.20 |  |  | 0.51 |
| Decreased | 12 (18%) | 9 (13%) |  | 19 (17%) | 8 (8.9%) |  | 3 (6.0%) | 5 (11%) |  |
| No change | 34 (52%) | 35 (51%) |  | 63 (58%) | 60 (67%) |  | 32 (64%) | 24 (53%) |  |
| Improved | 19 (29%) | 25 (36%) |  | 27 (25%) | 22 (24%) |  | 15 (30%) | 16 (36%) |  |
| **It is easy to find information about COVID-19 vaccines** |  |  | 0.11 |  |  | 0.56 |  |  | NA |
| Decreased | 11 (17%) | 4 (5.8%) |  | 22 (20%) | 14 (16%) |  | NA | NA |  |
| No change | 37 (57%) | 42 (61%) |  | 67 (61%) | 55 (61%) |  | NA | NA |  |
| Improved | 17 (26%) | 23 (33%) |  | 20 (18%) | 21 (23%) |  | NA | NA |  |
| **COVID-19 vaccines cause genetic change** |  |  | NA |  |  | 0.089 |  |  | 0.15 |
| Decreased | NA | NA |  | 12 (11%) | 9 (10%) |  | 10 (20%) | 3 (6.7%) |  |
| No change | NA | NA |  | 74 (68%) | 72 (80%) |  | 31 (62%) | 35 (78%) |  |
| Improved | NA | NA |  | 23 (21%) | 9 (10%) |  | 9 (18%) | 7 (16%) |  |
| **COVID-19 vaccines cause death** |  |  | 0.39 |  |  | 0.29 |  |  | 0.51 |
| Decreased | 10 (15%) | 7 (10%) |  | 11 (10%) | 10 (11%) |  | 4 (8.0%) | 5 (11%) |  |
| No change | 35 (54%) | 45 (65%) |  | 71 (65%) | 66 (73%) |  | 36 (72%) | 35 (78%) |  |
| Improved | 20 (31%) | 17 (25%) |  | 27 (25%) | 14 (16%) |  | 10 (20%) | 5 (11%) |  |
| **COVID-19 vaccines cause infertility** |  |  | **0.022** |  |  | 0.32 |  |  | 0.24 |
| Decreased | 16 (25%) | 5 (7.2%) |  | 15 (14%) | 9 (10%) |  | 6 (12%) | 3 (6.7%) |  |
| No change | 34 (52%) | 46 (67%) |  | 69 (63%) | 66 (73%) |  | 39 (78%) | 32 (71%) |  |
| Improved | 15 (23%) | 18 (26%) |  | 25 (23%) | 15 (17%) |  | 5 (10%) | 10 (22%) |  |
| **COVID-19 vaccines cause infection** |  |  | NA |  |  | 0.16 |  |  | 0.69 |
| Decreased | NA | NA |  | 16 (15%) | 13 (14%) |  | 8 (16%) | 5 (11%) |  |
| No change | NA | NA |  | 64 (59%) | 63 (70%) |  | 36 (72%) | 36 (80%) |  |
| Improved | NA | NA |  | 29 (27%) | 14 (16%) |  | 6 (12%) | 4 (8.9%) |  |
| **COVID-19 vaccines were approved without completing clinical trials** |  |  | 0.67 |  |  | 0.74 |  |  | 0.45 |
| Decreased | 10 (15%) | 10 (14%) |  | 13 (12%) | 14 (16%) |  | 5 (10%) | 4 (8.9%) |  |
| No change | 37 (57%) | 44 (64%) |  | 77 (71%) | 60 (67%) |  | 36 (72%) | 28 (62%) |  |
| Improved | 18 (28%) | 15 (22%) |  | 19 (17%) | 16 (18%) |  | 9 (18%) | 13 (29%) |  |
| **Safety of COVID-19 vaccines has not been confirmed** |  |  | NA |  |  | NA |  |  | 0.18 |
| Decreased | NA | NA |  | NA | NA |  | 5 (10%) | 9 (20%) |  |
| No change | NA | NA |  | NA | NA |  | 38 (76%) | 26 (58%) |  |
| Improved | NA | NA |  | NA | NA |  | 7 (14%) | 10 (22%) |  |

Distributions of changes in the secondary outcome variables from the pre- and post-intervention questionnaires. Statistical significance tested using Fisher’s exact test, p <0.05 bolded. Values are numbers (percentages) of participants unless stated otherwise.

**Supplementary Table 4.** **Secondary outcome variables for senior groups in Thailand and Hong Kong**

|  | **Thailand** | | | **Hong Kong** | | |
| --- | --- | --- | --- | --- | --- | --- |
|  | **Control**  **(n = 73)** | **Intervention**  **(n = 59)** | **P Value** | **Control**  **(n = 106)** | **Intervention**  **(n = 82)** | **P Value** |
| **My family member does not need to be vaccinated** |  |  | 0·72 |  |  | 0.20 |
| Decreased | 27 (37%) | 18 (31%) |  | 22 (21%) | 20 (24%) |  |
| No change | 26 (36%) | 22 (37%) |  | 48 (45%) | 44 (54%) |  |
| Improved | 20 (27%) | 19 (32%) |  | 36 (34%) | 18 (22%) |  |
| **My family member might get COVID-19** |  |  | 0·48 |  |  | 0.76 |
| Decreased | 12 (16%) | 14 (24%) |  | 31 (29%) | 28 (34%) |  |
| No change | 26 (36%) | 22 (37%) |  | 47 (44%) | 35 (43%) |  |
| Improved | 35 (48%) | 23 (39%) |  | 28 (26%) | 19 (23%) |  |
| **COVID-19 is a serious disease** |  |  | 0·093 |  |  | 0.11 |
| Decreased | 17 (23%) | 10 (17%) |  | 30 (28%) | 13 (16%) |  |
| No change | 28 (38%) | 34 (58%) |  | 58 (55%) | 56 (68%) |  |
| Improved | 28 (38%) | 15 (25%) |  | 18 (17%) | 13 (16%) |  |
| **I will be less anxious if my family member is vaccinated** |  |  | 0·85 |  |  | 0.69 |
| Decreased | 17 (23%) | 12 (20%) |  | 21 (20%) | 15 (18%) |  |
| No change | 34 (47%) | 27 (46%) |  | 60 (57%) | 43 (52%) |  |
| Improved | 22 (30%) | 20 (34%) |  | 25 (24%) | 24 (29%) |  |
| **Vaccination of elderly can control the spread of COVID-19** |  |  | 0·91 |  |  | 0.15 |
| Decreased | 11 (15%) | 7 (12%) |  | 25 (24%) | 11 (13%) |  |
| No change | 38 (52%) | 32 (54%) |  | 54 (51%) | 52 (63%) |  |
| Improved | 24 (33%) | 20 (34%) |  | 27 (25%) | 19 (23%) |  |
| **My family member will get vaccinated if many others are vaccinated** |  |  | 0·82 |  |  | 0.20 |
| Decreased | 14 (19%) | 12 (20%) |  | 33 (31%) | 16 (20%) |  |
| No change | 38 (52%) | 33 (56%) |  | 54 (51%) | 49 (60%) |  |
| Improved | 21 (29%) | 14 (24%) |  | 19 (18%) | 17 (21%) |  |
| **My family member will get vaccinated if there is a vaccine mandate** |  |  | **0·036** |  |  | 0.45 |
| Decreased | 9 (12%) | 18 (31%) |  | 12 (11%) | 14 (17%) |  |
| No change | 37 (51%) | 25 (42%) |  | 66 (62%) | 45 (55%) |  |
| Improved | 27 (37%) | 16 (27%) |  | 28 (26%) | 23 (28%) |  |
| **I think COVID-19 vaccination should be mandatory** |  |  | 0·57 |  |  | 0.63 |
| Decreased | 12 (16%) | 14 (24%) |  | 24 (23%) | 14 (17%) |  |
| No change | 37 (51%) | 29 (49%) |  | 54 (51%) | 46 (56%) |  |
| Improved | 24 (33%) | 16 (27%) |  | 28 (26%) | 22 (27%) |  |
| **It is easy to find information about COVID-19 vaccines** |  |  | 0·86 |  |  | 0.37 |
| Decreased | 12 (16%) | 10 (17%) |  | 14 (13%) | 15 (18%) |  |
| No change | 39 (53%) | 34 (58%) |  | 61 (58%) | 39 (48%) |  |
| Improved | 22 (30%) | 15 (25%) |  | 31 (29%) | 28 (34%) |  |
| **COVID-19 vaccines cause genetic change** |  |  | NA |  |  | 0.25 |
| Decreased | NA | NA |  | 17 (16%) | 7 (8.5%) |  |
| No change | NA | NA |  | 69 (65%) | 61 (74%) |  |
| Improved | NA | NA |  | 20 (19%) | 14 (17%) |  |
| **COVID-19 vaccines cause death** |  |  | 0·92 |  |  | 0.81 |
| Decreased | 6 (8·2%) | 4 (6·8%) |  | 11 (10%) | 11 (13%) |  |
| No change | 53 (73%) | 42 (71%) |  | 72 (68%) | 54 (66%) |  |
| Improved | 14 (19%) | 13 (22%) |  | 23 (22%) | 17 (21%) |  |
| **COVID-19 vaccines cause infertility** |  |  | 0·51 |  |  | 0.94 |
| Decreased | 16 (22%) | 8 (14%) |  | 17 (16%) | 14 (17%) |  |
| No change | 48 (66%) | 43 (73%) |  | 63 (59%) | 47 (57%) |  |
| Improved | 9 (12%) | 8 (14%) |  | 26 (25%) | 21 (26%) |  |
| **COVID-19 vaccines cause infection** |  |  | NA |  |  | 0.52 |
| Decreased | NA | NA |  | 11 (10%) | 11 (13%) |  |
| No change | NA | NA |  | 70 (66%) | 57 (70%) |  |
| Improved | NA | NA |  | 25 (24%) | 14 (17%) |  |
| **COVID-19 vaccines were approved without completing clinical trials** |  |  | **0·026** |  |  | 0.58 |
| Decreased | 13 (18%) | 2 (3·4%) |  | 13 (12%) | 8 (9.8%) |  |
| No change | 47 (64%) | 42 (71%) |  | 63 (59%) | 55 (67%) |  |
| Improved | 13 (18%) | 15 (25%) |  | 30 (28%) | 19 (23%) |  |

Distributions of changes in the secondary outcome variables from the pre- and post-intervention questionnaires. Statistical significance tested using Fisher’s exact test, p <0.05 bolded. Values are numbers (percentages) of participants unless stated otherwise

**Supplementary Table 5. Primary outcome variables for child groups in Thailand, Hong Kong, and Singapore (excluding Filipino and Indonesian sub-communities)**

|  | **Thailand** | | | **Hong Kong*** | | | **Singapore*** | | |
| --- | --- | --- | --- | --- | --- | --- | --- | --- | --- |
|  | **Control**  **(n = 65)** | **Intervention**  **(n = 69)** | **P Value** | **Control**  **(n = 47)** | **Intervention**  **(n = 49)** | **P Value** | **Control**  **(n = 34)** | **Intervention**  **(n = 38)** | **P Value** |
| **Vaccines are important** |  |  | 0.82 |  |  | 0.20 |  |  | 0.16 |
| Decreased | 9 (14%) | 7 (10%) |  | 11 (23%) | 7 (14%) |  | 1 (2.9%) | 5 (13%) |  |
| No change | 41 (63%) | 45 (65%) |  | 22 (47%) | 32 (65%) |  | 24 (71%) | 28 (74%) |  |
| Improved | 15 (23%) | 17 (25%) |  | 14 (30%) | 10 (20%) |  | 9 (26%) | 5 (13%) |  |
| **Vaccines are safe** |  |  | 0.27 |  |  | **0.047** |  |  | 0.11 |
| Decreased | 9 (14%) | 7 (10%) |  | 10 (21%) | 6 (12%) |  | 3 (8.8%) | 10 (26%) |  |
| No change | 34 (52%) | 46 (67%) |  | 23 (49%) | 36 (73%) |  | 23 (68%) | 18 (47%) |  |
| Improved | 22 (34%) | 16 (23%) |  | 14 (30%) | 7 (14%) |  | 8 (24%) | 10 (26%) |  |
| **Vaccines are effective** |  |  | **0.023** |  |  | **0.034** |  |  | NA |
| Decreased | 11 (17%) | 3 (4.3%) |  | 12 (26%) | 5 (10%) |  | NA | NA |  |
| No change | 32 (49%) | 47 (68%) |  | 21 (45%) | 34 (69%) |  | NA | NA |  |
| Improved | 22 (34%) | 19 (28%) |  | 14 (30%) | 10 (20%) |  | NA | NA |  |
| **Vaccines are effective in reducing severe conditions** |  |  | 0.065 |  |  | **0.031** |  |  | 0.78 |
| Decreased | 12 (18%) | 4 (5.8%) |  | 10 (21%) | 7 (14%) |  | 4 (12%) | 6 (16%) |  |
| No change | 36 (55%) | 40 (58%) |  | 24 (51%) | 37 (76%) |  | 22 (65%) | 25 (66%) |  |
| Improved | 17 (26%) | 25 (36%) |  | 13 (28%) | 5 (10%) |  | 8 (24%) | 7 (18%) |  |
| **Vaccines are effective in preventing infection** |  |  | NA |  |  | NA |  |  | 0.79 |
| Decreased | NA | NA |  | NA | NA |  | 7 (21%) | 9 (24%) |  |
| No change | NA | NA |  | NA | NA |  | 14 (41%) | 18 (47%) |  |
| Improved | NA | NA |  | NA | NA |  | 13 (38%) | 11 (29%) |  |
| **Vaccines are effective regardless of manufacturers** |  |  | **<0.001** |  |  | NA |  |  | NA |
| Decreased | 36 (55%) | 10 (14%) |  | NA | NA |  | NA | NA |  |
| No change | 13 (20%) | 40 (58%) |  | NA | NA |  | NA | NA |  |
| Improved | 16 (25%) | 19 (28%) |  | NA | NA |  | NA | NA |  |
| **Vaccines are effective against all variants** |  |  | **<0.001** |  |  | NA |  |  | NA |
| Decreased | 31 (48%) | 6 (8.7%) |  | NA | NA |  | NA | NA |  |
| No change | 19 (29%) | 44 (64%) |  | NA | NA |  | NA | NA |  |
| Improved | 15 (23%) | 19 (28%) |  | NA | NA |  | NA | NA |  |
| **Has your child received a COVID-19 vaccine?** |  |  | 0.79 |  |  | 0.80 |  |  | 1 |
| Decreased | 2 (3.1%) | 3 (4.3%) |  | 2 (4.3%) | 4 (8.2%) |  | 1 (2.9%) | 1 (2.6%) |  |
| No change | 48 (74%) | 53 (77%) |  | 38 (81%) | 38 (78%) |  | 32 (94%) | 35 (92%) |  |
| Improved | 15 (23%) | 13 (19%) |  | 7 (15%) | 7 (14%) |  | 1 (2.9%) | 2 (5.3%) |  |
| **Has your child received/do you intend for your child to receive a COVID-19 vaccine?** |  |  | 0.60 |  |  | 0.057 |  |  | 0.93 |
| Decreased | 3 (4.6%) | 6 (8.7%) |  | 4 (8.5%) | 13 (27%) |  | 2 (5.9%) | 3 (7.9%) |  |
| No change | 39 (60%) | 42 (61%) |  | 25 (53%) | 24 (49%) |  | 23 (68%) | 27 (71%) |  |
| Improved | 23 (35%) | 21 (30%) |  | 18 (38%) | 12 (24%) |  | 9 (26%) | 8 (21%) |  |

Distributions of changes in the levels of COVID-19 vaccine confidence and acceptance from the pre- and post-intervention questionnaires. Statistical significance tested using Fisher’s exact test, p <0.05 bolded. Values are numbers (percentages) of participants unless stated otherwise. * Filipino and Indonesian sub-communities were excluded.

**Supplementary Table 6. Primary outcome variables for senior groups in Thailand and Hong Kong (excluding Filipino and Indonesian sub-communities)**

|  | **Thailand** | | | **Hong Kong*** | | |
| --- | --- | --- | --- | --- | --- | --- |
|  | **Control**  **(n = 73)** | **Intervention**  **(n = 59)** | **P Value** | **Control**  **(n = 75)** | **Intervention**  **(n = 51)** | **P Value** |
| **Vaccines are important** |  |  | 0.42 |  |  | 0.96 |
| Decreased | 12 (16%) | 12 (20%) |  | 18 (24%) | 11 (22%) |  |
| No change | 32 (44%) | 30 (51%) |  | 47 (63%) | 33 (65%) |  |
| Improved | 29 (40%) | 17 (29%) |  | 10 (13%) | 7 (14%) |  |
| **Vaccines are safe** |  |  | 0.43 |  |  | 0.23 |
| Decreased | 8 (11%) | 10 (17%) |  | 12 (16%) | 15 (29%) |  |
| No change | 39 (53%) | 33 (56%) |  | 47 (63%) | 27 (53%) |  |
| Improved | 26 (36%) | 16 (27%) |  | 16 (21%) | 9 (18%) |  |
| **Vaccines are effective** |  |  | 0.46 |  |  | 0.76 |
| Decreased | 8 (11%) | 11 (19%) |  | 13 (17%) | 11 (22%) |  |
| No change | 42 (58%) | 32 (54%) |  | 43 (57%) | 26 (51%) |  |
| Improved | 23 (32%) | 16 (27%) |  | 19 (25%) | 14 (27%) |  |
| **Vaccines are effective in reducing severe conditions** |  |  | **0.024** |  |  | 0.54 |
| Decreased | 15 (21%) | 7 (12%) |  | 16 (21%) | 7 (14%) |  |
| No change | 27 (37%) | 36 (61%) |  | 44 (59%) | 34 (67%) |  |
| Improved | 31 (42%) | 16 (27%) |  | 15 (20%) | 10 (20%) |  |
| **Vaccines are effective regardless of manufacturers** |  |  | 0.41 |  |  | NA |
| Decreased | 8 (11%) | 7 (12%) |  | NA | NA |  |
| No change | 32 (44%) | 32 (54%) |  | NA | NA |  |
| Improved | 33 (45%) | 20 (34%) |  | NA | NA |  |
| **Vaccines are effective against all variants** |  |  | 0.44 |  |  | NA |
| Decreased | 12 (16%) | 6 (10%) |  | NA | NA |  |
| No change | 36 (49%) | 35 (59%) |  | NA | NA |  |
| Improved | 25 (34%) | 18 (31%) |  | NA | NA |  |
| **Has your family member received a COVID-19 vaccine?** |  |  | 0.19 |  |  | 0.18 |
| Decreased | 3 (4.1%) | 0 (0%) |  | 3 (4.0%) | 0 (0%) |  |
| No change | 58 (79%) | 53 (90%) |  | 66 (88%) | 43 (84%) |  |
| Improved | 12 (16%) | 6 (10%) |  | 6 (8.0%) | 8 (16%) |  |
| **Has your family member received/do you intend for your family member to receive a COVID-19 vaccine?** |  |  | 0.33 |  |  | 0.50 |
| Decreased | 6 (8.2%) | 2 (3.4%) |  | 3 (4.0%) | 1 (2.0%) |  |
| No change | 38 (52%) | 37 (63%) |  | 58 (77%) | 36 (71%) |  |
| Improved | 29 (40%) | 20 (34%) |  | 14 (19%) | 14 (27%) |  |

Distributions of changes in the levels of COVID-19 vaccine confidence and acceptance from the pre- and post-intervention questionnaires. Statistical significance tested using Fisher’s exact test, p <0.05 bolded. Values are numbers (percentages) of participants unless stated otherwise. * Filipino and Indonesian sub-communities were excluded.

**Supplementary Table 7. Secondary outcome variables for child groups in Thailand, Hong Kong, and Singapore (excluding Filipino and Indonesian sub-communities)**

|  | **Thailand** | | | **Hong Kong*** | | | **Singapore*** | | |
| --- | --- | --- | --- | --- | --- | --- | --- | --- | --- |
|  | **Control**  **(n = 65)** | **Intervention**  **(n = 69)** | **P Value** | **Control**  **(n = 47)** | **Intervention**  **(n = 49)** | **P Value** | **Control**  **(n = 34)** | **Intervention**  **(n = 38)** | **P Value** |
| **My child does not need to be vaccinated** |  |  | **0·006** |  |  | 0·55 |  |  | 0·77 |
| Decreased | 21 (32%) | 9 (13%) |  | 10 (21%) | 9 (18%) |  | 6 (18%) | 5 (13%) |  |
| No change | 22 (34%) | 40 (58%) |  | 25 (53%) | 22 (45%) |  | 15 (44%) | 20 (53%) |  |
| Improved | 22 (34%) | 20 (29%) |  | 12 (26%) | 18 (37%) |  | 13 (38%) | 13 (34%) |  |
| **My child might get COVID-19** |  |  | 0·34 |  |  | 0·62 |  |  | **0·015** |
| Decreased | 17 (26%) | 12 (17%) |  | 11 (23%) | 8 (16%) |  | 11 (32%) | 3 (7·9%) |  |
| No change | 24 (37%) | 33 (48%) |  | 24 (51%) | 29 (59%) |  | 15 (44%) | 17 (45%) |  |
| Improved | 24 (37%) | 24 (35%) |  | 12 (26%) | 12 (24%) |  | 8 (24%) | 18 (47%) |  |
| **COVID-19 is a serious disease** |  |  | **0·008** |  |  | 0·30 |  |  | 0·65 |
| Decreased | 16 (25%) | 5 (7·2%) |  | 9 (19%) | 8 (16%) |  | 6 (18%) | 5 (13%) |  |
| No change | 29 (45%) | 46 (67%) |  | 26 (55%) | 34 (69%) |  | 19 (56%) | 19 (50%) |  |
| Improved | 20 (31%) | 18 (26%) |  | 12 (26%) | 7 (14%) |  | 9 (26%) | 14 (37%) |  |
| **I will be less anxious if my child is vaccinated** |  |  | 0·59 |  |  | 0·18 |  |  | 0·74 |
| Decreased | 8 (12%) | 5 (7·2%) |  | 9 (19%) | 5 (10%) |  | 8 (24%) | 6 (16%) |  |
| No change | 33 (51%) | 35 (51%) |  | 24 (51%) | 34 (69%) |  | 21 (62%) | 26 (68%) |  |
| Improved | 24 (37%) | 29 (42%) |  | 14 (30%) | 10 (20%) |  | 5 (15%) | 6 (16%) |  |
| **Vaccination of children can control the spread of COVID-19** |  |  | 0·87 |  |  | 0·073 |  |  | 1 |
| Decreased | 12 (18%) | 10 (14%) |  | 13 (28%) | 5 (10%) |  | 6 (18%) | 7 (18%) |  |
| No change | 34 (52%) | 38 (55%) |  | 23 (49%) | 33 (67%) |  | 19 (56%) | 22 (58%) |  |
| Improved | 19 (29%) | 21 (30%) |  | 11 (23%) | 11 (22%) |  | 9 (26%) | 9 (24%) |  |
| **My child will get vaccinated if many others are vaccinated** |  |  | 0·59 |  |  | 0·33 |  |  | 0·85 |
| Decreased | 9 (14%) | 6 (8·7%) |  | 10 (21%) | 6 (12%) |  | 5 (15%) | 8 (21%) |  |
| No change | 37 (57%) | 39 (57%) |  | 23 (49%) | 31 (63%) |  | 19 (56%) | 20 (53%) |  |
| Improved | 19 (29%) | 24 (35%) |  | 14 (30%) | 12 (24%) |  | 10 (29%) | 10 (26%) |  |
| **My child will get vaccinated if there is a vaccine mandate** |  |  | 0·19 |  |  | 0·60 |  |  | 0·94 |
| Decreased | 10 (15%) | 4 (5·8%) |  | 9 (19%) | 7 (14%) |  | 6 (18%) | 5 (13%) |  |
| No change | 33 (51%) | 42 (61%) |  | 26 (55%) | 25 (51%) |  | 19 (56%) | 23 (61%) |  |
| Improved | 22 (34%) | 23 (33%) |  | 12 (26%) | 17 (35%) |  | 9 (26%) | 10 (26%) |  |
| **I think COVID-19 vaccination should be mandatory** |  |  | 0·56 |  |  | 0·088 |  |  | 0·60 |
| Decreased | 12 (18%) | 9 (13%) |  | 9 (19%) | 3 (6·1%) |  | 2 (5·9%) | 5 (13%) |  |
| No change | 34 (52%) | 35 (51%) |  | 26 (55%) | 36 (73%) |  | 21 (62%) | 23 (61%) |  |
| Improved | 19 (29%) | 25 (36%) |  | 12 (26%) | 10 (20%) |  | 11 (32%) | 10 (26%) |  |
| **It is easy to find information about COVID-19 vaccines** |  |  | 0·11 |  |  | 0·52 |  |  | NA |
| Decreased | 11 (17%) | 4 (5·8%) |  | 13 (28%) | 9 (18%) |  | NA | NA |  |
| No change | 37 (57%) | 42 (61%) |  | 23 (49%) | 25 (51%) |  | NA | NA |  |
| Improved | 17 (26%) | 23 (33%) |  | 11 (23%) | 15 (31%) |  | NA | NA |  |
| **COVID-19 vaccines cause genetic change** |  |  | NA |  |  | **0·031** |  |  | 0·26 |
| Decreased | NA | NA |  | 4 (8·5%) | 5 (10%) |  | 6 (18%) | 3 (7·9%) |  |
| No change | NA | NA |  | 28 (60%) | 39 (80%) |  | 20 (59%) | 29 (76%) |  |
| Improved | NA | NA |  | 15 (32%) | 5 (10%) |  | 8 (24%) | 6 (16%) |  |
| **COVID-19 vaccines cause death** |  |  | 0·39 |  |  | 0·94 |  |  | 0·52 |
| Decreased | 10 (15%) | 7 (10%) |  | 4 (8·5%) | 3 (6·1%) |  | 3 (8·8%) | 4 (11%) |  |
| No change | 35 (54%) | 45 (65%) |  | 33 (70%) | 36 (73%) |  | 24 (71%) | 30 (79%) |  |
| Improved | 20 (31%) | 17 (25%) |  | 10 (21%) | 10 (20%) |  | 7 (21%) | 4 (11%) |  |
| **COVID-19 vaccines cause infertility** |  |  | **0·022** |  |  | 0·11 |  |  | 0·52 |
| Decreased | 16 (25%) | 5 (7·2%) |  | 8 (17%) | 6 (12%) |  | 3 (8·8%) | 3 (7·9%) |  |
| No change | 34 (52%) | 46 (67%) |  | 25 (53%) | 36 (73%) |  | 27 (79%) | 27 (71%) |  |
| Improved | 15 (23%) | 18 (26%) |  | 14 (30%) | 7 (14%) |  | 4 (12%) | 8 (21%) |  |
| **COVID-19 vaccines cause infection** |  |  | NA |  |  | 0·16 |  |  | 0·40 |
| Decreased | NA | NA |  | 5 (11%) | 8 (16%) |  | 7 (21%) | 5 (13%) |  |
| No change | NA | NA |  | 29 (62%) | 35 (71%) |  | 23 (68%) | 31 (82%) |  |
| Improved | NA | NA |  | 13 (28%) | 6 (12%) |  | 4 (12%) | 2 (5·3%) |  |
| **COVID-19 vaccines were approved without completing clinical trials** |  |  | 0·67 |  |  | 0·74 |  |  | 0·24 |
| Decreased | 10 (15%) | 10 (14%) |  | 4 (8·5%) | 7 (14%) |  | 2 (5·9%) | 4 (11%) |  |
| No change | 37 (57%) | 44 (64%) |  | 33 (70%) | 32 (65%) |  | 27 (79%) | 23 (61%) |  |
| Improved | 18 (28%) | 15 (22%) |  | 10 (21%) | 10 (20%) |  | 5 (15%) | 11 (29%) |  |
| **Safety of COVID-19 vaccines has not been confirmed** |  |  | NA |  |  | NA |  |  | 0·19 |
| Decreased | NA | NA |  | NA | NA |  | 3 (8·8%) | 8 (21%) |  |
| No change | NA | NA |  | NA | NA |  | 25 (74%) | 20 (53%) |  |
| Improved | NA | NA |  | NA | NA |  | 6 (18%) | 10 (26%) |  |

Distributions of changes in the secondary outcomes from the pre- and post-intervention questionnaires. Statistical significance tested using Fisher’s exact test, p <0.05 bolded. Values are numbers (percentages) of participants unless stated otherwise. * Filipino and Indonesian sub-communities were excluded.

**Supplementary Table 8.** **Secondary outcome variables for senior groups in Thailand and Hong Kong** **(excluding Filipino and Indonesian sub-communities)**

|  | **Thailand** | | | **Hong Kong*** | | |
| --- | --- | --- | --- | --- | --- | --- |
|  | **Control**  **(n = 73)** | **Intervention**  **(n = 59)** | **P Value** | **Control**  **(n = 75)** | **Intervention**  **(n = 51)** | **P Value** |
| **My family member does not need to be vaccinated** |  |  | 0·72 |  |  | 0·34 |
| Decreased | 27 (37%) | 18 (31%) |  | 16 (21%) | 13 (25%) |  |
| No change | 26 (36%) | 22 (37%) |  | 32 (43%) | 26 (51%) |  |
| Improved | 20 (27%) | 19 (32%) |  | 27 (36%) | 12 (24%) |  |
| **My family member might get COVID-19** |  |  | 0·48 |  |  | 0·95 |
| Decreased | 12 (16%) | 14 (24%) |  | 24 (32%) | 17 (33%) |  |
| No change | 26 (36%) | 22 (37%) |  | 30 (40%) | 21 (41%) |  |
| Improved | 35 (48%) | 23 (39%) |  | 21 (28%) | 13 (25%) |  |
| **COVID-19 is a serious disease** |  |  | 0·093 |  |  | 0·14 |
| Decreased | 17 (23%) | 10 (17%) |  | 25 (33%) | 9 (18%) |  |
| No change | 28 (38%) | 34 (58%) |  | 37 (49%) | 33 (65%) |  |
| Improved | 28 (38%) | 15 (25%) |  | 13 (17%) | 9 (18%) |  |
| **I will be less anxious if my family member is vaccinated** |  |  | 0·85 |  |  | 0·73 |
| Decreased | 17 (23%) | 12 (20%) |  | 16 (21%) | 9 (18%) |  |
| No change | 34 (47%) | 27 (46%) |  | 43 (57%) | 28 (55%) |  |
| Improved | 22 (30%) | 20 (34%) |  | 16 (21%) | 14 (27%) |  |
| **Vaccination of elderly can control the spread of COVID-19** |  |  | 0·91 |  |  | 0·44 |
| Decreased | 11 (15%) | 7 (12%) |  | 19 (25%) | 8 (16%) |  |
| No change | 38 (52%) | 32 (54%) |  | 40 (53%) | 31 (61%) |  |
| Improved | 24 (33%) | 20 (34%) |  | 16 (21%) | 12 (24%) |  |
| **My family member will get vaccinated if many others are vaccinated** |  |  | 0·82 |  |  | 0·19 |
| Decreased | 14 (19%) | 12 (20%) |  | 24 (32%) | 9 (18%) |  |
| No change | 38 (52%) | 33 (56%) |  | 39 (52%) | 31 (61%) |  |
| Improved | 21 (29%) | 14 (24%) |  | 12 (16%) | 11 (22%) |  |
| **My family member will get vaccinated if there is a vaccine mandate** |  |  | **0·036** |  |  | 0·56 |
| Decreased | 9 (12%) | 18 (31%) |  | 7 (9·3%) | 8 (16%) |  |
| No change | 37 (51%) | 25 (42%) |  | 48 (64%) | 29 (57%) |  |
| Improved | 27 (37%) | 16 (27%) |  | 20 (27%) | 14 (27%) |  |
| **I think COVID-19 vaccination should be mandatory** |  |  | 0·57 |  |  | 0·72 |
| Decreased | 12 (16%) | 14 (24%) |  | 18 (24%) | 10 (20%) |  |
| No change | 37 (51%) | 29 (49%) |  | 37 (49%) | 29 (57%) |  |
| Improved | 24 (33%) | 16 (27%) |  | 20 (27%) | 12 (24%) |  |
| **It is easy to find information about COVID-19 vaccines** |  |  | 0·86 |  |  | 0·65 |
| Decreased | 12 (16%) | 10 (17%) |  | 11 (15%) | 10 (20%) |  |
| No change | 39 (53%) | 34 (58%) |  | 41 (55%) | 24 (47%) |  |
| Improved | 22 (30%) | 15 (25%) |  | 23 (31%) | 17 (33%) |  |
| **COVID-19 vaccines cause genetic change** |  |  | NA |  |  | 0·079 |
| Decreased | NA | NA |  | 10 (13%) | 4 (7·8%) |  |
| No change | NA | NA |  | 48 (64%) | 42 (82%) |  |
| Improved | NA | NA |  | 17 (23%) | 5 (9·8%) |  |
| **COVID-19 vaccines cause death** |  |  | 0·92 |  |  | 0·45 |
| Decreased | 6 (8·2%) | 4 (6·8%) |  | 10 (13%) | 8 (16%) |  |
| No change | 53 (73%) | 42 (71%) |  | 48 (64%) | 36 (71%) |  |
| Improved | 14 (19%) | 13 (22%) |  | 17 (23%) | 7 (14%) |  |
| **COVID-19 vaccines cause infertility** |  |  | 0·51 |  |  | 0·80 |
| Decreased | 16 (22%) | 8 (14%) |  | 13 (17%) | 11 (22%) |  |
| No change | 48 (66%) | 43 (73%) |  | 41 (55%) | 28 (55%) |  |
| Improved | 9 (12%) | 8 (14%) |  | 21 (28%) | 12 (24%) |  |
| **COVID-19 vaccines cause infection** |  |  | NA |  |  | 0·69 |
| Decreased | NA | NA |  | 7 (9·3%) | 5 (9·8%) |  |
| No change | NA | NA |  | 53 (71%) | 39 (76%) |  |
| Improved | NA | NA |  | 15 (20%) | 7 (14%) |  |
| **COVID-19 vaccines were approved without completing clinical trials** |  |  | **0·026** |  |  | 0·11 |
| Decreased | 13 (18%) | 2 (3·4%) |  | 10 (13%) | 6 (12%) |  |
| No change | 47 (64%) | 42 (71%) |  | 42 (56%) | 37 (73%) |  |
| Improved | 13 (18%) | 15 (25%) |  | 23 (31%) | 8 (16%) |  |

Distributions of changes in the secondary outcomes from the pre- and post-intervention questionnaires. Statistical significance tested using Fisher’s exact test, p <0.05 bolded. Values are numbers (percentages) of participants unless stated otherwise. * Filipino and Indonesian sub-communities were excluded.

**Supplementary Table 9. Associations between vaccine confidence and acceptance and sociodemographic factors, misinformation, risk perception, and chatbot use in Thailand 1) child and 2) senior groups.**

| 1. Thailand Child Group | | | | | | | | | | | | | | | | | | | | | | | | | |  |  |  |
| --- | --- | --- | --- | --- | --- | --- | --- | --- | --- | --- | --- | --- | --- | --- | --- | --- | --- | --- | --- | --- | --- | --- | --- | --- | --- | --- | --- | --- |
|  |  | **"Vaccines are important"** | | | **"Vaccines are safe"** | | | **"Vaccines are effective"** | | | **"Vaccines are effective in reducing severe conditions"** | | | **"Vaccines are effective regardless of manufacturers"** | | | | **"Vaccines are effective against all variants"** | | | | **"Has your child received/do you intend for your child to receive a COVID-19 vaccine?"** | | | |  |  |  |
|  | n (%) | Odds Ratio (95% CI) | | | Odds Ratio (95% CI) | | | Odds Ratio (95% CI) | | | Odds Ratio (95% CI) | | | Odds Ratio (95% CI) | | | | Odds Ratio (95% CI) | | | | Odds Ratio (95% CI) | | | |  |  |  |
| SOCIODEMOGRAPHICS | | |  | | |  | | | |  | | |  | | | | | |  | | | |  |  | |  | |  |
| Respondent’s ethnicity |  |  | | |  | | |  | | |  | | |  | | | |  | | | |  | | | |  |  |  |
| Thai | 125 (93%) | - | | | - | | | - | | | - | | | - | | | | - | | | | - | | | |  |  |  |
| Other | 9 (6.7%) | 1.18 (0.31 to 4.41) | | | 1.26 (0.35 to 4.56) | | | **12.91 (3.56 to 46.78)** | | | 1.69 (0.47 to 6.1) | | | **2.99 (1.28 to 6.97)** | | | | **4.9 (1.74 to 13.85)** | | | | **13.19 (4.92 to 35.3)** | | | |  |  |  |
| Respondent’s education level |  |  | | |  | | |  | | |  | | |  | | | |  | | | |  | | | |  |  |  |
| Below college level | 66 (49%) | - | | | - | | | - | | | - | | | - | | | | - | | | | - | | | |  |  |  |
| College or above | 64 (48%) | **2.15 (1.16 to 3.97)** | | | 1.38 (0.82 to 2.34) | | | 1.26 (0.7 to 2.26) | | | 0.66 (0.39 to 1.11) | | | 0.86 (0.62 to 1.19) | | | | 1 (0.69 to 1.44) | | | | 0.92 (0.62 to 1.36) | | | |  |  |  |
| Other | 4 (3.0%) | 3.02 (0.38 to 24.26) | | | 0.3 (0.04 to 2.12) | | | 0.69 (0.08 to 6.23) | | | 0.34 (0.05 to 2.34) | | | **8.23 (2.32 to 29.16)** | | | | **35.21 (6.77 to 183.02)** | | | | 0.31 (0.08 to 1.26) | | | |  |  |  |
| Is the respondent working in a healthcare setting? |  |  | | |  | | |  | | |  | | |  | | | |  | | | |  | | | |  |  |  |
| Yes | 17 (13%) | - | | | - | | | - | | | - | | | - | | | | - | | | | - | | | |  |  |  |
| No | 108 (81%) | **0.15 (0.07 to 0.34)** | | | 0.78 (0.37 to 1.67) | | | 0.46 (0.2 to 1.05) | | | **0.46 (0.22 to 0.98)** | | | 0.88 (0.54 to 1.44) | | | | 1 (0.57 to 1.75) | | | | 1.23 (0.69 to 2.2) | | | |  |  |  |
| Financial situation |  |  | | |  | | |  | | |  | | |  | | | |  | | | |  | | | |  |  |  |
| Low | 29 (22%) | - | | | - | | | - | | | - | | | - | | | | - | | | | - | | | |  |  |  |
| Middle | 39 (29%) | **0.2 (0.09 to 0.45)** | | | 0.74 (0.36 to 1.49) | | | 0.79 (0.37 to 1.68) | | | 1.22 (0.59 to 2.5) | | | 0.89 (0.57 to 1.4) | | | | **1.85 (1.1 to 3.09)** | | | | 1.04 (0.61 to 1.76) | | | |  |  |  |
| High | 59 (44%) | **0.24 (0.1 to 0.56)** | | | 0.53 (0.26 to 1.11) | | | **0.37 (0.16 to 0.83)** | | | 0.91 (0.43 to 1.91) | | | 1.08 (0.68 to 1.71) | | | | 1.08 (0.65 to 1.81) | | | | 0.65 (0.38 to 1.13) | | | |  |  |  |
| Other | 7 (5.2%) | **0.15 (0.02 to 0.85)** | | | 1.6 (0.35 to 7.35) | | | 0.38 (0.06 to 2.34) | | | **4.62 (1.02 to 20.89)** | | | 0.87 (0.31 to 2.41) | | | | 1.6 (0.48 to 5.34) | | | | 1.84 (0.6 to 5.7) | | | |  |  |  |
| Geographical location |  |  | | |  | | |  | | |  | | |  | | | |  | | | |  | | | |  |  |  |
| Urban | 51 (38%) | - | | | - | | | - | | | - | | | - | | | | - | | | | - | | | |  |  |  |
| Non-urban | 83 (62%) | 0.97 (0.52 to 1.79) | | | **0.55 (0.32 to 0.94)** | | | 1.01 (0.55 to 1.85) | | | 0.83 (0.49 to 1.42) | | | 1.23 (0.88 to 1.73) | | | | **1.94 (1.33 to 2.85)** | | | | 0.8 (0.54 to 1.2) | | | |  |  |  |
| Child’s gender |  |  | | |  | | |  | | |  | | |  | | | |  | | | |  | | | |  |  |  |
| Female | 64 (48%) | - | | | - | | | - | | | - | | | - | | | | - | | | | - | | | |  |  |  |
| Male | 63 (47%) | 0.95 (0.53 to 1.69) | | | **1.74 (1.04 to 2.92)** | | | 1.27  (0.72 to 2.24) | | | 0.77  (0.46 to 1.28) | | | 1.22  (0.89 to 1.69) | | | | 1.25  (0.87 to 1.78) | | | | 0.89 (0.61 to 1.3) | | | |  |  |  |
| Other | 7 (5.2%) | 1.53 (0.44 to 5.31) | | | 0.51 (0.16 to 1.65) | | | 0.72  (0.2 to 2.63) | | | **0.22**  **(0.07 to 0.71)** | | | **2.78**  **(1.32 to 5.85)** | | | | **3.31**  **(1.39 to 7.9)** | | | | 0.97 (0.41 to 2.29) | | | |  |  |  |
| Child’s age | **-** | 0.89 (0.78 to 1.02) | | | 1.01 (0.9 to 1.13) | | | 0.99  (0.87 to 1.13) | | | 1.06  (0.94 to 1.19) | | | **0.91**  **(0.85 to 0.98)** | | | | 0.94  (0.86 to 1.02) | | | | 1.01 (0.93 to 1.11) | | | |  |  |  |
| MISINFORMATION |  |  | | |  | | |  | | |  | | |  | | | |  | | | |  | | | |  |  |  |
| "COVID-19 vaccines cause death" | - | 1.25 (0.79 to 1.96) | | | 0.93 (0.63 to 1.37) | | | 0.84 (0.55 to 1.3) | | | 1.15 (0.78 to 1.69) | | | 0.99 (0.77 to 1.26) | | | | 1.41 (1.07 to 1.84) | | | | 0.96 (0.72 to 1.28) | | | |  |  |  |
| "COVID-19 vaccines cause infertility" | - | 0.63 (0.37 to 1.08) | | | 0.72 (0.45 to 1.16) | | | 1.02  (0.6 to 1.72) | | | **0.51**  **(0.32 to 0.81)** | | | 1.01  (0.75 to 1.34) | | | | 1.03  (0.75 to 1.43) | | | | 0.86  (0.61 to 1.21) | | | |  |  |  |
| "COVID-19 vaccines were approved without completing clinical trials" | - | **0.6 (0.39 to 0.91)** | | | 0.91 (0.63 to 1.33) | | | 0.84 (0.56 to 1.27) | | | **1.61 (1.1 to 2.35)** | | | 0.89 (0.71 to 1.13) | | | | **0.61 (0.47 to 0.8)** | | | | 1.1 (0.84 to 1.45) | | | |  |  |  |
| RISK PERCEPTION |  |  | | |  | | |  | | |  | | |  | | | |  | | | |  | | | |  |  |  |
| "My child might get COVID-19" | - | 1.04 (0.8 to 1.36) | | | 0.93 (0.74 to 1.18) | | | 0.78 (0.6 to 1.01) | | | 1.02 (0.81 to 1.29) | | | **0.81 (0.7 to 0.94)** | | | | **0.8 (0.68 to 0.95)** | | | | 1.04 (0.87 to 1.23) | | | |  |  |  |
| "COVID-19 is a serious disease" | - | **0.62 (0.45 to 0.86)** | | | **0.7 (0.53 to 0.92)** | | | 0.84 (0.62 to 1.14) | | | **0.71 (0.54 to 0.93)** | | | **0.65 (0.55 to 0.78)** | | | | **0.67 (0.55 to 0.82)** | | | | 1.04 (0.85 to 1.28) | | | |  |  |  |
| Chatbot Use |  |  | | |  | | |  | | |  | | |  | |  | | | |  | | | | | |  |  |  |
| Control | 65 (49%) | - | | | - | | | - | | | - | | | - | | - | | | | - | | | | | |  |  |  |
| Intervention | 69 (51%) | **2.40 (1.34 to 4.32)** | | | 0.73 (0.44 to 1.21) | | | 1.25 (0.71 to 2.19) | | | **2.07 (1.23 to 3.48)** | | | **4.04 (2.87 to 5.69)** | | **3.21 (2.22 to 4.66)** | | | | **0.66 (0.45 to 0.96)** | | | | | |  |  |  |
|  | | | | | | | | | | | | | | | | | | | | | | | | | |  |  |  |
| 2) Thailand Senior Group | | | | | | | | | | | | | | | | | | | | | | | | | |  |  |  |
|  |  | **"Vaccines are important"** | | | **"Vaccines are safe"** | | | **"Vaccines are effective"** | | | **"Vaccines are effective in reducing severe conditions"** | | | **"Vaccines are effective regardless of manufacturers"** | | | | **"Vaccines are effective against all variants"** | | | | **"Has your family member received/do you intend for your family member to receive a COVID-19 vaccine?"** | | | |  |  |  |
|  | n (%) | Odds Ratio (95% CI) | | | Odds Ratio (95% CI) | | | Odds Ratio (95% CI) | | | Odds Ratio (95% CI) | | | Odds Ratio (95% CI) | | | | Odds Ratio (95% CI) | | | | Odds Ratio (95% CI) | | | |  |  |  |
| SOCIODEMOGRAPHICS | | | |  | | |  | |  | | |  | | |  | | | | | | | | | |  |  |  | |
| Respondent’s ethnicity |  |  | | |  | | |  | | |  | | |  | | | |  | | | |  | | | |  |  |  |
| Thai | 119 (90%) | - | | | - | | | - | | | - | | | - | | | | - | | | | - | | | |  |  |  |
| Other | 13 (9.8%) | **12.3 (4.74 to 31.91)** | | | 2.25 (0.8 to 6.34) | | | 0.54 (0.17 to 1.7) | | | 1.11 (0.37 to 3.32) | | | 0.65 (0.21 to 1.97) | | | | 1.06 (0.28 to 4) | | | | 0.87 (0.65 to 1.17) | | | |  |  |  |
| Respondent’s gender |  |  | | |  | | |  | | |  | | |  | | | |  | | | |  | | | |  |  |  |
| Female | 74 (56%) | - | | | - | | | - | | | - | | | - | | | | - | | | | - | | | |  |  |  |
| Male | 58 (44%) | 1.12 (0.74 to 1.67) | | | **0.6 (0.39 to 0.9)** | | | 1.52 (0.98 to 2.36) | | | 1.52 (0.99 to 2.34) | | | 1.05 (0.68 to 1.63) | | | | 0.86 (0.51 to 1.45) | | | | **1.23 (1.09 to 1.39)** | | | |  |  |  |
| Respondent’s age |  |  | | |  | | |  | | |  | | |  | | | |  | | | |  | | | |  |  |  |
| 35 and under | 84 (64%) | - | | | - | | | - | | | - | | | - | | | | - | | | | - | | | |  |  |  |
| Over 35 | 48 (36%) | **0.56 (0.36 to 0.86)** | | | **0.56 (0.36 to 0.88)** | | | **0.6 (0.37 to 0.96)** | | | **0.61 (0.39 to 0.98)** | | | **0.58 (0.37 to 0.93)** | | | | 1.12 (0.65 to 1.96) | | | | **0.69 (0.6 to 0.79)** | | | |  |  |  |
| Respondent’s education level |  |  | | |  | | |  | | |  | | |  | | | |  | | | |  | | | |  |  |  |
| Below college level | 66 (50%) | - | | | - | | | - | | | - | | | - | | | | - | | | | - | | | |  |  |  |
| College or above | 62 (47%) | 0.8 (0.52 to 1.24) | | | 0.7 (0.46 to 1.09) | | | 0.88 (0.56 to 1.39) | | | 0.66 (0.42 to 1.04) | | | 0.66 (0.42 to 1.05) | | | | 0.7 (0.41 to 1.21) | | | | **0.7 (0.61 to 0.79)** | | | |  |  |  |
| Other | 4 (3.0%) | 0.25 (0.04 to 1.48) | | | **0.07 (0.01 to 0.41)** | | | 0.33 (0.05 to 2.28) | | | **0.11 (0.02 to 0.6)** | | | **0.15 (0.02 to 0.93)** | | | | 4.61 (0.59 to 35.8) | | | | **27.67 (8.97 to 85.41)** | | | |  |  |  |
| Is the respondent working in a healthcare setting? |  |  | | |  | | |  | | |  | | |  | | | |  | | | |  | | | |  |  |  |
| Yes | 24 (18%) | - | | | - | | | - | | | - | | | - | | | | - | | | |  | | | |  |  |  |
| No | 108 (82%) | 0.61 (0.36 to 1.04) | | | 1.29 (0.74 to 2.23) | | | 1.09 (0.61 to 1.94) | | | **0.38 (0.21 to 0.68)** | | | 0.9 (0.5 to 1.6) | | | | 0.85 (0.43 to 1.68) | | | | **1.24 (1.06 to 1.46)** | | | |  |  |  |
| Financial situation |  |  | | |  | | |  | | |  | | |  | | | |  | | | |  | | | |  |  |  |
| Low | 24 (18%) | - | | | - | | | - | | | - | | | - | | | | - | | | | - | | | |  |  |  |
| Middle | 45 (34%) | 0.81 (0.46 to 1.42) | | | 1.18 (0.66 to 2.12) | | | **2.17 (1.15 to 4.1)** | | | **2.1 (1.13 to 3.9)** | | | 1.03 (0.54 to 1.95) | | | | 0.73 (0.35 to 1.5) | | | | 1.08 (0.89 to 1.31) | | | |  |  |  |
| High | 54 (41%) | **0.48 (0.27 to 0.86)** | | | 0.57 (0.31 to 1.05) | | | 1.59 (0.84 to 3.02) | | | **2.9 (1.54 to 5.47)** | | | **0.46 (0.24 to 0.88)** | | | | 0.51 (0.24 to 1.05) | | | | 0.99 (0.82 to 1.19) | | | |  |  |  |
| Other | 9 (6.8%) | **0.1 (0.04 to 0.26)** | | | **0.35 (0.14 to 0.84)** | | | 1.88 (0.75 to 4.72) | | | 0.93 (0.38 to 2.28) | | | 0.46 (0.18 to 1.17) | | | | 0.59 (0.2 to 1.78) | | | | **0.59 (0.46 to 0.75)** | | | |  |  |  |
| Geographical location |  |  | | |  | | |  | | |  | | |  | | | |  | | | |  | | | |  |  |  |
| Urban | 53 (40%) | - | | | - | | | - | | | - | | | - | | | | - | | | | - | | | |  |  |  |
| Non-urban | 79 (60%) | **0.6 (0.41 to 0.9)** | | | 1.14 (0.76 to 1.72) | | | 0.9 (0.59 to 1.38) | | | 0.99 (0.65 to 1.51) | | | 1.46 (0.95 to 2.24) | | | | 1 (0.6 to 1.66) | | | | **1.24 (1.1 to 1.4)** | | | |  |  |  |
| Senior's gender |  |  | | |  | | |  | | |  | | |  | | | |  | | | |  | | | |  |  |  |
| Female | 76 (58%) | - | | | - | | | - | | | - | | | - | | | | - | | | | - | | | |  |  |  |
| Male | 56 (42%) | 1.32 (0.89 to 1.97) | | | 1.3 (0.86 to 1.95) | | | 1 (0.65 to 1.52) | | | 1.44 (0.94 to 2.2) | | | 1.17 (0.76 to 1.8) | | | | 0.66 (0.4 to 1.11) | | | | **0.65 (0.57 to 0.73)** | | | |  |  |  |
| Senior's age |  |  | | |  | | |  | | |  | | |  | | | |  | | | |  | | | |  |  |  |
| 60 to 80 | 105 (80%) | - | | | - | | | - | | | - | | | - | | | | - | | | | - | | | |  |  |  |
| Over 80 | 27 (20%) | **2.91 (1.78 to 4.77)** | | | 1.04 (0.62 to 1.74) | | | 0.74 (0.43 to 1.28) | | | 0.9 (0.53 to 1.51) | | | 1.34 (0.78 to 2.28) | | | | 0.83 (0.44 to 1.6) | | | | **2.47 (2.06 to 2.95)** | | | |  |  |  |
| MISINFORMATION |  |  | | |  | | |  | | |  | | |  | | | |  | | | |  | | | |  |  |  |
| "COVID-19 vaccines cause death" | - | 1.36 (1 to 1.84) | | | 1.37 (1 to 1.88) | | | 1.28 (0.92 to 1.78) | | | 0.85 (0.61 to 1.17) | | | **0.55 (0.4 to 0.77)** | | | | **1.68 (1.13 to 2.49)** | | | | 1.08 (0.99 to 1.19) | | | |  |  |  |
| "COVID-19 vaccines cause infertility" | - | 1.4 (0.92 to 2.14) | | | 0.75 (0.49 to 1.16) | | | 0.89 (0.56 to 1.42) | | | **1.8 (1.14 to 2.83)** | | | 1.45 (0.91 to 2.31) | | | | 0.63 (0.36 to 1.09) | | | | 0.96 (0.84 to 1.09) | | | |  |  |  |
| "COVID-19 vaccines were approved without completing clinical trials" | - | **0.47 (0.34 to 0.65)** | | | **0.59 (0.43 to 0.82)** | | | **0.47 (0.33 to 0.66)** | | | **0.58 (0.41 to 0.81)** | | | 0.74 (0.52 to 1.03) | | | | 0.82 (0.55 to 1.22) | | | | 1.01 (0.91 to 1.11) | | | |  |  |  |
| RISK PERCEPTION |  |  | | |  | | |  | | |  | | |  | | | |  | | | |  | | | |  |  |  |
| "My family member might get COVID-19" | - | **1.26 (1.06 to 1.49)** | | | **0.77 (0.64 to 0.92)** | | | 0.95 (0.79 to 1.15) | | | 0.95 (0.79 to 1.15) | | | 1.17 (0.97 to 1.4) | | | | 0.92 (0.73 to 1.15) | | | | 1.03 (0.98 to 1.09) | | | |  |  |  |
| "COVID-19 is a serious disease" | - | 0.94 (0.77 to 1.14) | | | 0.85 (0.69 to 1.04) | | | **0.74 (0.6 to 0.92)** | | | **0.64 (0.51 to 0.79)** | | | **0.76 (0.61 to 0.95)** | | | | **0.76 (0.59 to 0.98)** | | | | 1.01 (0.95 to 1.07) | | | |  |  |  |
| Chatbot use |  |  | | |  | | |  | | |  | | |  | | |  | | | |  | | | | |  |  |  |
| Control | 73 (55%) | - | | | - | | | - | | | - | | | - | | | - | | | | - | | | | |  |  |  |
| Intervention | 59 (45%) | 0.74 (0.49 to 1.11) | | | **0.63 (0.41 to 0.96)** | | | 0.77 (0.5 to 1.2) | | | 1.02 (0.66 to 1.58) | | | **0.57 (0.36 to 0.88)** | | | 1.07 (0.63 to 1.79) | | | | 0.91 (0.8 to 1.04) | | | | |  |  |  |

Adjusted for respondent’s sex, age, and employment status for Thailand child group, and respondent’s employment status for Thailand senior group.

**Supplementary Table 10. Associations between vaccine confidence and acceptance and sociodemographic factors, misinformation, risk perception, and chatbot use in Hong Kong 1) child and 2) senior groups.**

| 1. Hong Kong Child Group | | | | | | | | | | | | | | | | |
| --- | --- | --- | --- | --- | --- | --- | --- | --- | --- | --- | --- | --- | --- | --- | --- | --- |
|  |  | | **"Vaccines are important"** | | **"Vaccines are safe"** | | **"Vaccines are effective"** | | **"Vaccines are effective in reducing severe conditions"** | | | **"Has your child received/do you intend for your child to receive a COVID-19 vaccine?"** | | | | |
|  | n (%) | | Odds Ratio (95% CI) | | Odds Ratio (95% CI) | | Odds Ratio (95% CI) | | Odds Ratio (95% CI) | | | Odds Ratio (95% CI) | | | | |
| SOCIODEMOGRAPHICS |  | |  | |  | |  | |  | | |  | | | | |
| Respondent’s ethnicity |  | |  | |  | |  | |  | | |  | | | | |
| Chinese | 87 (44%) | | - | | - | | - | | - | | | - | | | | |
| Other | 112 (56%) | | 0.88 (0.33 to 2.34) | | **5.68 (2.01 to 16.1)** | | **2.73 (1.11 to 6.7)** | | 0.86 (0.33 to 2.27) | | | 1.02 (0.55 to 1.87) | | | | |
| Respondent’s education level |  | |  | |  | |  | |  | | |  | | | | |
| Below college level | 99 (50%) | | - | | - | | - | | - | | | - | | | | |
| College or above | 93 (47%) | | **0.26 (0.14 to 0.48)** | | 1.67 (0.86 to 3.22) | | 1.2 (0.69 to 2.09) | | **0.34 (0.18 to 0.64)** | | | **0.33 (0.22 to 0.48)** | | | | |
| Other | 7 (3.5%) | | 0.37 (0.03 to 4.88) | | 0.12 (0.01 to 2.05) | | 0.63 (0.06 to 6.31) | | **0.02 (0 to 0.22)** | | | 0.79 (0.16 to 3.92) | | | | |
| Is the respondent working in a healthcare setting? |  | |  | |  | |  | |  | | |  | | | | |
| Yes | 10 (5.0%) | | - | | - | | - | | - | | | - | | | | |
| No | 189 (95%) | | **4.37 (1.46 to 13.1)** | | **0.28 (0.08 to 0.93)** | | 0.5 (0.18 to 1.33) | | 1.51 (0.52 to 4.4) | | | **6.99 (3.4 to 14.36)** | | | | |
| Family income |  | |  | |  | |  | |  | | |  | | | | |
| Under 30K HKD | 127 (64%) | | - | | - | | - | | - | | | - | | | | |
| 30-59K HKD | 29 (15%) | | **2.75 (1.34 to 5.62)** | | **2.51 (1.18 to 5.35)** | | **3.75 (1.95 to 7.23)** | | 1.27 (0.61 to 2.64) | | | **3.48 (2.22 to 5.44)** | | | | |
| 60K HKD or above | 29 (15%) | | 0.78 (0.35 to 1.77) | | 1.7 (0.72 to 4) | | 1.81 (0.86 to 3.83) | | 1.96 (0.85 to 4.52) | | | **6.94 (4.08 to 11.83)** | | | | |
| Other | 14 (7.0%) | | 4.65 (0.66 to 32.67) | | **12.66 (1.59 to 100.58)** | | **9.1 (1.58 to 52.46)** | | **19.58 (3.84 to 99.91)** | | | 1.28 (0.37 to 4.43) | | | | |
| Child’s gender |  | |  | |  | |  | |  | | |  | | | | |
| Female | 86 (43%) | | - | | - | | - | | - | | | - | | | | |
| Male | 102 (51%) | | **3.06 (1.67 to 5.6)** | | 1.24 (0.66 to 2.3) | | 1.45 (0.85 to 2.49) | | 1.02 (0.56 to 1.87) | | | 1.09 (0.76 to 1.57) | | | | |
| Other | 11 (5.5%) | | **5.51 (1.28 to 23.7)** | | 0.99 (0.21 to 4.71) | | **3.74 (1.06 to 13.14)** | | **9.71 (2.5 to 37.71)** | | | **12.15 (5.23 to 28.22)** | | | | |
| Child’s age | - | | 1.07 (1 to 1.15) | | **0.91 (0.84 to 0.98)** | | 0.95 (0.89 to 1.01) | | 1.07 (1 to 1.15) | | | 0.96 (0.91 to 1) | | | | |
| MISINFORMATION |  | |  | |  | |  | |  | | |  | | | | |
| "COVID-19 vaccines cause genetic change" | - | | **0.52 (0.32 to 0.84)** | | **0.49 (0.3 to 0.8)** | | **0.65 (0.42 to 0.99)** | | 1.02 (0.62 to 1.66) | | | **0.65 (0.48 to 0.87)** | | | | |
| "COVID-19 vaccines cause death" | - | | 1.09 (0.71 to 1.66) | | 1.12 (0.72 to 1.75) | | 1.42 (0.96 to 2.09) | | 1.04 (0.67 to 1.6) | | | **0.68 (0.52 to 0.88)** | | | | |
| "COVID-19 vaccines cause infertility" | - | | 0.81 (0.51 to 1.28) | | **0.46 (0.29 to 0.74)** | | **0.65 (0.43 to 0.98)** | | 0.98 (0.61 to 1.57) | | | 1.19 (0.9 to 1.58) | | | | |
| "COVID-19 vaccines cause infection" | - | | **2.24 (1.39 to 3.62)** | | **6.09 (3.51 to 10.55)** | | **1.67 (1.09 to 2.56)** | | 0.83 (0.51 to 1.33) | | | **1.77 (1.32 to 2.36)** | | | | |
| "COVID-19 vaccines were approved without completing clinical trials" | - | | **0.58 (0.4 to 0.85)** | | **0.43 (0.29 to 0.64)** | | **0.53 (0.37 to 0.74)** | | **0.68 (0.46 to 0.98)** | | | **0.67 (0.53 to 0.84)** | | | | |
| RISK PERCEPTION |  | |  | |  | |  | |  | | |  | | | | |
| "My child might get COVID-19" | - | | **1.56 (1.16 to 2.1)** | | 0.88 (0.65 to 1.21) | | **1.55 (1.18 to 2.03)** | | 1.33 (0.98 to 1.79) | | | 1.15 (0.95 to 1.38) | | | | |
| "COVID-19 is a serious disease" | - | | 0.85 (0.64 to 1.13) | | 1.34 (0.99 to 1.81) | | **0.73 (0.57 to 0.95)** | | 0.84 (0.63 to 1.11) | | | **1.39 (1.17 to 1.66)** | | | | |
| Chatbot Use |  | |  | |  | |  | |  | | |  | | | | |
| Control | 109 (54.8%) | | - | | - | | - | | - | | | - | | | | |
| Intervention | 90 (45.2%) | | 0.58 (0.33 to 1.02) | | 0.7 (0.39 to 1.28) | | 0.79 (0.47 to 1.32) | | 0.76 (0.42 to 1.37) | | | **0.24 (0.17 to 0.34)** | | | | |
|  | | | |  | | | |  | | |  | | |  |  |  |
| 2) Hong Kong Senior Group | | | | | | | | | | | | | | | | |
|  |  | | **"Vaccines are important"** | | **"Vaccines are safe"** | | **"Vaccines are effective"** | | **"Vaccines are effective in preventing infection"** | | | **"Has your family member received/do you intend for your family member to receive a COVID-19 vaccine?"** | | | | |
|  | n (%) | | Odds Ratio (95% CI) | | Odds Ratio (95% CI) | | Odds Ratio (95% CI) | | Odds Ratio (95% CI) | | | Odds Ratio (95% CI) | | | | |
| SOCIODEMOGRAPHICS | |  | |  | |  | |  | |  | | |  | | | |
| Respondent’s ethnicity |  | |  | |  | |  | |  | | |  | | | | |
| Chinese | 113 (60%) | | - | | - | | - | | - | | | - | | | | |
| Other | 74 (40%) | | 0.47 (0.21 to 1.07) | | 0.58 (0.28 to 1.19) | | 0.88 (0.44 to 1.76) | | **0.36 (0.16 to 0.8)** | | | **0.14 (0.1 to 0.2)** | | | | |
| Respondent’s gender |  | |  | |  | |  | |  | | |  | | | | |
| Female | 116 (62%) | | - | | - | | - | | - | | | - | | | | |
| Male | 68 (36%) | | 1.28 (0.78 to 2.11) | | **1.61 (1.04 to 2.48)** | | 1.44 (0.94 to 2.19) | | 0.82 (0.5 to 1.33) | | | **1.76 (1.39 to 2.23)** | | | | |
| Other | 4 (2.1%) | | 1.54 (0.21 to 11.03) | | **6.59 (1.19 to 36.41)** | | 0.65 (0.11 to 3.9) | | 2.03 (0.29 to 14.13) | | | 1.46 (0.66 to 3.23) | | | | |
| Respondent’s age |  | |  | |  | |  | |  | | |  | | | | |
| 35 and under | 78 (42%) | | - | | - | | - | | - | | | - | | | | |
| Over 35 | 109 (58%) | | 1 (0.61 to 1.65) | | **3.19 (2.03 to 5.01)** | | 1.14 (0.74 to 1.75) | | 1.2 (0.74 to 1.95) | | | **1.8 (1.42 to 2.29)** | | | | |
| Respondent’s education level |  | |  | |  | |  | |  | | |  | | | | |
| Below college level | 86 (46%) | | - | | - | | - | | - | | | - | | | | |
| College or above | 96 (51%) | | **0.31 (0.18 to 0.55)** | | **0.18 (0.11 to 0.29)** | | **0.41 (0.26 to 0.67)** | | 1.39 (0.81 to 2.39) | | | 0.82 (0.63 to 1.07) | | | | |
| Other | 6 (3.2%) | | 0.69 (0.13 to 3.69) | | **0.14 (0.03 to 0.6)** | | **16.36 (4.46 to 60.02)** | | 3.28 (0.65 to 16.49) | | | **0.33 (0.16 to 0.69)** | | | | |
| Is the respondent working in a healthcare setting? |  | |  | |  | |  | |  | | |  | | | | |
| Yes | 11 (5.9%) | | - | | - | | - | | - | | | - | | | | |
| No | 177 (94%) | | 1.17 (0.38 to 3.57) | | **4.93 (1.86 to 13.07)** | | **0.23 (0.1 to 0.51)** | | 0.68 (0.23 to 2.01) | | | **0.19 (0.12 to 0.31)** | | | | |
| Family income |  | |  | |  | |  | |  | | |  | | | | |
| Under 30K HKD | 113 (60%) | | - | | - | | - | | - | | | - | | | | |
| 30-59K HKD | 30 (16%) | | 0.73 (0.37 to 1.41) | | 1.27 (0.72 to 2.23) | | 1.01 (0.57 to 1.76) | | 0.71 (0.37 to 1.34) | | | 1.34 (0.97 to 1.83) | | | | |
| 60K HKD or above | 28 (15%) | | 1.63 (0.82 to 3.25) | | 1.6 (0.88 to 2.9) | | 0.89 (0.5 to 1.6) | | 0.93 (0.48 to 1.83) | | | **0.48 (0.34 to 0.66)** | | | | |
| Other | 17 (9.0%) | | **6.34 (2.12 to 19.03)** | | **9.74 (3.66 to 25.95)** | | 1.61 (0.56 to 4.67) | | **0.17 (0.06 to 0.53)** | | | **8.4 (4.86 to 14.53)** | | | | |
| Senior’s gender |  | |  | |  | |  | |  | | |  | | | | |
| Female | 107 (57%) | | - | | - | | - | | - | | | - | | | | |
| Male | 81 (43%) | | **2.07 (1.25 to 3.42)** | | 0.89 (0.58 to 1.36) | | 1.24 (0.82 to 1.88) | | 0.86 (0.53 to 1.4) | | | **0.75 (0.59 to 0.94)** | | | | |
| Senior’s age |  | |  | |  | |  | |  | | |  | | | | |
| 60 to 80 | 138 (73%) | | - | | - | | - | | - | | | - | | | | |
| Over 80 | 50 (27%) | | 0.83 (0.49 to 1.41) | | **0.3 (0.18 to 0.48)** | | **0.46 (0.28 to 0.73)** | | 1.3 (0.78 to 2.18) | | | 0.79 (0.61 to 1.02) | | | | |
| MISINFORMATION |  | |  | |  | |  | |  | | |  | | | | |
| "COVID-19 vaccines cause genetic change" | - | | 0.92 (0.59 to 1.46) | | 1.33 (0.9 to 1.97) | | 0.83 (0.56 to 1.22) | | 0.65 (0.41 to 1.01) | | | **0.7 (0.56 to 0.88)** | | | | |
| "COVID-19 vaccines cause death" | - | | 0.84 (0.55 to 1.28) | | 0.85 (0.59 to 1.22) | | **1.84 (1.28 to 2.64)** | | **1.91 (1.26 to 2.88)** | | | **0.78 (0.64 to 0.95)** | | | | |
| "COVID-19 vaccines cause infertility" | - | | 1.06 (0.68 to 1.66) | | 0.75 (0.51 to 1.12) | | **0.53 (0.36 to 0.78)** | | 0.96 (0.62 to 1.49) | | | 0.95 (0.76 to 1.17) | | | | |
| "COVID-19 vaccines cause infection" | - | | 0.75 (0.48 to 1.18) | | 1.08 (0.73 to 1.59) | | **0.58 (0.4 to 0.83)** | | 0.65 (0.42 to 1) | | | 0.98 (0.79 to 1.21) | | | | |
| "COVID-19 vaccines were approved without completing clinical trials" | - | | 0.92 (0.65 to 1.3) | | 1.07 (0.8 to 1.45) | | 0.86 (0.64 to 1.17) | | 0.77 (0.55 to 1.09) | | | 1.13 (0.97 to 1.33) | | | | |
| RISK PERCEPTION |  | |  | |  | |  | |  | | |  | | | | |
| "My family member might get COVID-19" | - | | **0.7 (0.54 to 0.91)** | | 0.86 (0.68 to 1.08) | | **0.62 (0.49 to 0.78)** | | **0.49 (0.38 to 0.65)** | | | 1.05 (0.93 to 1.19) | | | | |
| "COVID-19 is a serious disease" | - | | **1.58 (1.17 to 2.14)** | | **1.57 (1.21 to 2.05)** | | 0.99 (0.78 to 1.27) | | 1.04 (0.78 to 1.39) | | | 1.59 (1.39 to 1.83) | | | | |
| Chatbot Use |  | |  | |  | |  | |  | | |  | | | | |
| Control | 106 (56%) | | - | | - | | - | | - | | | - | | | | |
| Intervention | 82 (44%) | | **1.87 (1.12 to 3.12)** | | 0.97 (0.62 to 1.52) | | **1.72 (1.11 to 2.64)** | | 1.29 (0.78 to 2.13) | | | **3.26 (2.53 to 4.21)** | | | | |

Adjusted for respondent’s sex, age, and employment status for Hong Kong child group, and respondent’s employment status for Hong Kong senior group.

**Supplementary Table 11. Associations between vaccine confidence and acceptance and sociodemographic factors, misinformation, risk perception, and chatbot use in 1) Singapore child group and 2) sub-communities in Singapore and Hong Kong.**

| 1. Singapore Child Group | | | | | | |
| --- | --- | --- | --- | --- | --- | --- |
|  |  | **"Vaccines are important"** | **"Vaccines are safe"** | **"Vaccines are effective in reducing severe conditions"** | **"Vaccines are effective in preventing infection"** | **"Has your family member received/do you intend for your family member to receive a COVID-19 vaccine?"** |
|  | n (%) | Odds Ratio (95% CI) | Odds Ratio (95% CI) | Odds Ratio (95% CI) | Odds Ratio (95% CI) | Odds Ratio (95% CI) |
| SOCIODEMOGRAPHICS |  |  |  |  |  |  |
| Respondent’s ethnicity |  |  |  |  |  |  |
| Chinese | 44 (46%) | - | - | - | - | - |
| Malay | 7 (7.4%) | 1.22 (0.14 to 10.78) | **10.46 (1.14 to 95.79)** | 3.15 (0.43 to 23.05) | **14.27 (2.21 to 92.18)** | 1.71 (0.37 to 7.89) |
| Indians | 14 (15%) | 0.9 (0.15 to 5.55) | 4.6 (0.71 to 29.75) | 0.32 (0.05 to 2.14) | 1.37 (0.29 to 6.45) | **5.87 (1.55 to 22.25)** |
| Other | 30 (32%) | 0.22 (0.03 to 1.65) | 0.49 (0.08 to 2.93) | 0.25 (0.03 to 1.83) | 0.37 (0.08 to 1.83) | 2.12 (0.45 to 10.06) |
| Respondent’s age |  |  |  |  |  |  |
| 35 and under | 40 (42%) | - | - | - | - | - |
| Over 35 | 55 (58%) | **9.24 (2.08 to 41.1)** | 0.38 (0.1 to 1.41) | 0.82 (0.22 to 3.03) | 0.61 (0.2 to 1.91) | 0.42 (0.14 to 1.24) |
| Respondent’s education level |  |  |  |  |  |  |
| Below college level | 49 (52%) | - | - | - | - | - |
| College or above | 45 (47%) | 2.49 (0.67 to 9.23) | 1.73 (0.53 to 5.69) | 1.01 (0.31 to 3.27) | 2.06 (0.71 to 6.01) | **3.08 (1.04 to 9.12)** |
| Other | 1 (1.1%) | 0.13 (0 to 5870.35) | 5.52 (0 to 10739.49) | 0.1 (0 to 489.11) | 0.38 (0 to 240.6) | 0.71 (0.02 to 33.17) |
| Is the respondent working in a healthcare setting? |  |  |  |  |  |  |
| Yes | 15 (16%) | - | - | - | - | - |
| No | 80 (84%) | **0.1 (0.02 to 0.48)** | 2.99 (0.62 to 14.42) | 3.1 (0.58 to 16.58) | 1.27 (0.32 to 5.08) | 1.31 (0.4 to 4.26) |
| Child’s gender |  |  |  |  |  |  |
| Female | 33 (35%) | - | - | - | - | - |
| Male | 56 (59%) | 1.4 (0.46 to 4.29) | 1.24 (0.43 to 3.58) | 0.63 (0.21 to 1.9) | 0.56 (0.22 to 1.45) | 0.51 (0.2 to 1.29) |
| Other | 6 (6.3%) | 3.83 (0.47 to 30.97) | 0.33 (0.04 to 2.83) | **21.95 (3.06 to 157.31)** | 3.67 (0.53 to 25.61) | **14.14 (2.01 to 99.34)** |
| Child’s age |  | 0.82 (0.61 to 1.1) | 1.25 (0.95 to 1.64) | **1.37 (1.03 to 1.83)** | 1.21 (0.95 to 1.54) | 0.84 (0.66 to 1.07) |
| MISINFORMATION |  |  |  |  |  |  |
| "COVID-19 vaccines cause genetic change" | - | **7.28 (2.35 to 22.54)** | 1.86 (0.73 to 4.76) | **5.24 (1.81 to 15.16)** | 1.31 (0.58 to 2.96) | 0.57 (0.26 to 1.23) |
| "COVID-19 vaccines cause death" | - | 0.59 (0.22 to 1.56) | 0.93 (0.38 to 2.29) | 0.77 (0.32 to 1.85) | **0.37 (0.16 to 0.83)** | 0.34 (0.16 to 0.72) |
| "COVID-19 vaccines cause infertility" | - | 1.6 (0.52 to 4.95) | 0.54 (0.18 to 1.64) | 1.52 (0.56 to 4.18) | 0.66 (0.25 to 1.71) | **4.34 (1.64 to 11.45)** |
| "COVID-19 vaccines cause infection" | - | 0.34 (0.11 to 1.07) | 1.21 (0.42 to 3.51) | **0.23 (0.08 to 0.67)** | **0.26 (0.1 to 0.68)** | **2.44 (1.01 to 5.86)** |
| "COVID-19 vaccines were approved without completing clinical trials" | - | 1.03 (0.36 to 2.93) | **0.31 (0.1 to 0.95)** | 0.57 (0.19 to 1.69) | 1.11 (0.43 to 2.89) | 0.54 (0.22 to 1.32) |
| "Safety of COVID-19 vaccines has not been confirmed" | - | 0.94 (0.34 to 2.59) | 2.41 (0.88 to 6.65) | 1.88 (0.69 to 5.13) | **6.7 (2.48 to 18.1)** | 1.13 (0.52 to 2.44) |
| Risk perception |  |  |  |  |  |  |
| "My child might get COVID-19" | - | 0.69 (0.42 to 1.14) | 0.75 (0.45 to 1.27) | 0.86 (0.52 to 1.44) | 0.93 (0.6 to 1.44) | **0.42 (0.26 to 0.68)** |
| "COVID-19 is a serious disease" | - | 1.06 (0.53 to 2.13) | 1.15 (0.62 to 2.12) | 1.1 (0.58 to 2.1) | 1.17 (0.68 to 2.01) | 0.75 (0.44 to 1.28) |
| Chatbot Use |  |  |  |  |  |  |
| Control | 50 (52.6%) | - | - | - | - | - |
| Intervention | 45 (47.4%) | 0.52 (0.17 to 1.58) | 0.43 (0.15 to 1.24) | 1.32 (0.47 to 3.67) | 0.54 (0.22 to 1.36) | 1.1 (0.45 to 2.68) |
| 2) Sub-communities: Filipino and Indonesian residents in Singapore and Hong Kong | | | | | | |
|  |  | **“Vaccines are important”** | **“Vaccines are safe”** | **“Vaccines are effective in reducing severe conditions”** | **"Has your family member received/do you intend for your family member to receive a COVID-19 vaccine?"** |  |
| Respondent's gender | n (%) | Odds Ratio (95% CI) | Odds Ratio (95% CI) | Odds Ratio (95% CI) | Odds Ratio (95% CI) |  |
| Female | 181 (96%) | - | - | - | - |  |
| Male | 6 (3.2%) | 1.11 (0.66 to 1.85) | 1.02 (0.65 to 1.61) | 1.16 (0.69 to 1.97) | **0.53 (0.4 to 0.71)** |  |
| Other | 1 (0.5%) |  |  |  |  |  |
| Respondent's age |  | - | - | - | - |  |
| 35 and under | 90 (48%) | 2.18 (0.61 to 7.81) | 1.08 (0.32 to 3.68) | 1.34 (0.33 to 5.39) | 0.67 (0.32 to 1.41) |  |
| Over 35 | 98 (52%) | 0.29 (0.01 to 9.97) | 0.21 (0.01 to 5.35) | **0.02 (0 to 0.37)** | 0.66 (0.09 to 4.62) |  |
| Respondent's education Level |  |  |  |  |  |  |
| Below college level | 117 (62%) | - | - | - | - |  |
| College or above | 63 (34%) | 1.02 (0.6 to 1.71) | 0.94 (0.59 to 1.5) | 1.54 (0.9 to 2.63) | 0.8 (0.61 to 1.06) |  |
| Other | 8 (4.3%) |  |  |  |  |  |
| Respondent's employment status |  | - | - | - | - |  |
| Unemployed/Economically inactive | 78 (44%) | 1.25 (0.72 to 2.16) | **1.8 (1.11 to 2.94)** | 0.86 (0.49 to 1.52) | **1.42 (1.07 to 1.91)** |  |
| Employed | 98 (56%) | 1.24 (0.28 to 5.44) | 1.02 (0.27 to 3.95) | 1.02 (0.23 to 4.62) | **2.43 (1.2 to 4.96)** |  |
| Is the Respondent Working in a Healthcare Setting? |  |  |  |  |  |  |
| Yes | 8 (4.3%) | - | - | - | - |  |
| No | 180 (96%) | 0.92 (0.55 to 1.55) | 0.77 (0.49 to 1.22) | 0.79 (0.46 to 1.33) | **0.73 (0.56 to 0.96)** |  |
| MISINFORMATION |  |  |  |  |  |  |
| “COVID-19 vaccines cause genetic change” | - | 1.1 (0.68 to 1.78) | 1.21 (0.79 to 1.88) | 1.06 (0.64 to 1.74) | **0.72 (0.56 to 0.92)** |  |
| “COVID-19 vaccines cause death” | - | 0.69 (0.45 to 1.06) | 0.87 (0.59 to 1.27) | 0.74 (0.48 to 1.15) | 1 (0.8 to 1.25) |  |
| “COVID-19 vaccines cause infertility” | - | 1.08 (0.64 to 1.81) | 0.64 (0.41 to 1.02) | 1.49 (0.88 to 2.53) | 1.06 (0.81 to 1.4) |  |
| “COVID-19 vaccines cause infection” | - | 1.29 (0.8 to 2.09) | 1.26 (0.82 to 1.95) | 1.06 (0.65 to 1.73) | **1.3 (1.01 to 1.67)** |  |
| “COVID-19 vaccines were approved without completing clinical trials” | - | 0.95 (0.64 to 1.43) | 0.93 (0.64 to 1.33) | 0.82 (0.54 to 1.25) | 0.89 (0.72 to 1.1) |  |
| RISK PERCEPTION |  |  |  |  |  |  |
| “My family member might get COVID-19” | - | **1.35 (1.07 to 1.71)** | **1.28 (1.04 to 1.57)** | 1.19 (0.94 to 1.5) | 1.02 (0.91 to 1.15) |  |
| “COVID-19 is a serious disease” | - | 0.83 (0.6 to 1.14) | 0.83 (0.63 to 1.1) | **0.6 (0.43 to 0.83)** | 1.07 (0.9 to 1.28) |  |
| Chatbot Use |  |  |  |  |  |  |
| Control | 109 (58.0%) | - | - | - | - |  |
| Intervention | 79 (42.0%) | 1.08 (0.67 to 1.74) | 0.9 (0.6 to 1.37) | 1.1 (0.7 to 1.75) | **0.57 (0.45 to 0.73)** |  |

Adjusted for respondent’s sex and employment status, and housing in Singapore child group.

**Supplementary Table 12. Minimum renumeration amount for participants in each study group**

|  | Hong Kong | Singapore | Thailand |
| --- | --- | --- | --- |
| Control group | 54.20 HKD | 9.40 SGD | 91 THB |
| Intervention group | 77.30 HKD | 13.35 SGD | 129 THB |

**Supplementary Table 13. RE-AIM framework chatbot evaluation**

|  |  |  | Chatbot users in study locations | | |
| --- | --- | --- | --- | --- | --- |
|  | **Definition^1^** | **Metrics used** | **Hong Kong** | **Singapore** | **Thailand** |
| Reach | *"The absolute number, proportion, and representativeness of individuals who participate in a given intervention or program."* | the total number of unique chatbot users | 144 | 40 | 41 (Study participants) 1454 (Public users) |
|  |  | the mean numbers of messages exchanged | 15 | 12 | 6.7% (Study participants) 4.8% (Public users) |
| Effectiveness | *"The impact of an intervention on important outcomes"* | changes in vaccine confidence | Discussed in the result section | | |
|  |  | changes in vaccine acceptance | Discussed in the result section | | |
| Adoption | *"The absolute number, proportion, and representativeness of settings and intervention agents who initiate a program"* | the proportion of users who reported that they would use chatbots again *"I intend to user chatbot again"* | 107/147 (73%) | 31/38 (82%) | 103/119 (87%) |
|  |  | the proportion of users who reported that they would recommend our chatbots to family and friends "*I intend to recommend to family/friends*" | 101/147 (69%) | 30/38 (79%) | 95/119 (80%) |
| Implementation | *" The intervention agents' fidelity to and adaptations of an intervention and associated implementation strategies, including consistency of delivery as intended and the time and costs"* | congruence between the planned and actual implementation of the chatbots in terms of the time and cost of chatbot development | For the course of the chatbot development period from October 2021 to January 2022, the actual cost of the chatbot development and implementation and operational cost was HKD$257,441 for Hong Kong and Singapore and $16,666 THB (Free of charge for the implementation, and approximately 16,666 THB for operational costs) for Thailand, averages of 1,392HKD and 11THB per unique users, respectively. The chatbots were developed and updated according to the plan. However, during the study period, the chatbot in Thailand, ChatSure, was temporarily unavailable while Facebook reviewed ChatSure’s app credentials and use cases for the integration such as Facebook login, like and share buttons. | | |
| Maintenance | *"The extent to which a program or policy becomes institutionalized or part of the routine organizational practices and policies"* | the long-term potential of the chatbots. | Although Hong Kong and Singapore have newly developed a D^2^4H chatbot for the closed trial participants, ChatSure in Thailand has been a part of the Thai government's vaccination campaign initiative to address COVID-19-related concerns and questions. The content available on ChatSure may be expanded to other public health topics in the future. The D24H chatbot is under re-imagination and will be expanded to cover other vaccines such as HPV vaccines and potentially as a supplemental measure to existing vaccination campaigns. | | |

**Supplementary Table 14. Relationship between dose (number of meaningful conversations, excludes commands to change languages or other settings) and outcome for Hong Kong and Singapore intervention groups.**

| **Outcomes** | **Odds Ratio**  **(95% CI)** | **P value** | **Adjusted P Value*** |
| --- | --- | --- | --- |
| “Vaccines are important” | 0.98 (0.95 to 1) | 0.104 | 1.000 |
| “Vaccines are safe” | 1.03 (1 to 1.06) | 0.036 | 0.654 |
| “Vaccines are effective in reducing severe conditions” | 1.03 (1 to 1.06) | 0.091 | 1.000 |
| “Has your family member received/do you intend for your family member to receive a COVID-19 vaccine?” | 0.99 (0.94 to 1.04) | 0.591 | 1.000 |
| Vaccine acceptance | 0.99 (0.97 to 1.01) | 0.331 | 1.000 |
| “My family member does not need to be vaccinated” | 1.03 (1.01 to 1.04) | 0.004 | 0.064 |
| “My family member might get COVID-19” | 1.01 (0.99 to 1.02) | 0.562 | 1.000 |
| “COVID-19 is a serious disease” | 0.99 (0.96 to 1.02) | 0.628 | 1.000 |
| “I will be less anxious if my family member is vaccinated” | 0.99 (0.96 to 1.01) | 0.361 | 1.000 |
| “Vaccination of children can control the spread of COVID-19” | 1.03 (1 to 1.06) | 0.047 | 1.000 |
| “My family member will get vaccinated if many others are vaccinated” | 1.01 (0.99 to 1.04) | 0.378 | 1.000 |
| “My family member will get vaccinated if there is a vaccine mandate” | 1.02 (0.99 to 1.04) | 0.136 | 1.000 |
| “I think COVID-19 vaccination should be mandatory” | 0.98 (0.95 to 1) | 0.080 | 1.000 |
| “It is easy to find information about COVID-19 vaccines” | 1.02 (0.98 to 1.06) | 0.272 | 1.000 |
| “COVID-19 vaccines cause death” | 1.01 (0.97 to 1.04) | 0.720 | 1.000 |
| “COVID-19 vaccines cause infertility” | 1.03 (1 to 1.06) | 0.086 | 1.000 |
| “COVID-19 vaccines cause infection” | 0.99 (0.96 to 1.03) | 0.716 | 1.000 |
| “COVID-19 vaccines were approved without completing clinical trials” | 1.02 (0.99 to 1.05) | 0.172 | 1.000 |

Adjusted using the conservative Bonferroni method.

**Supplementary Figure 1. Percentage of fully vaccinated population (two doses) and cumulative COVID-19 cases per million in Thailand, Hong Kong, and Singapore from January 2022 to June 2022.**


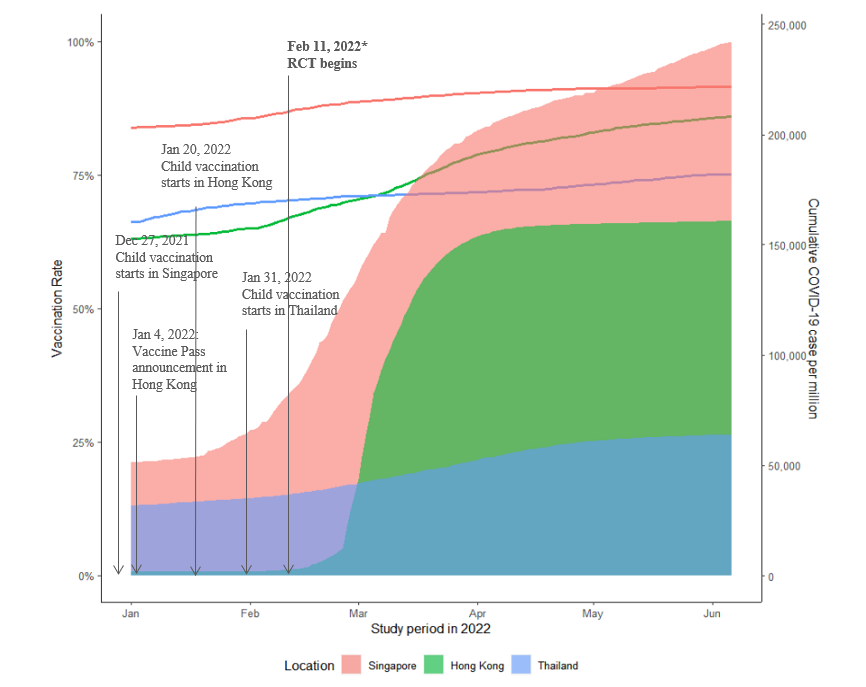


**Supplementary Figure 2. Sensitivity analysis for vaccine confidence and acceptance measures with and without Filipino and Indonesian subgroup.**

**
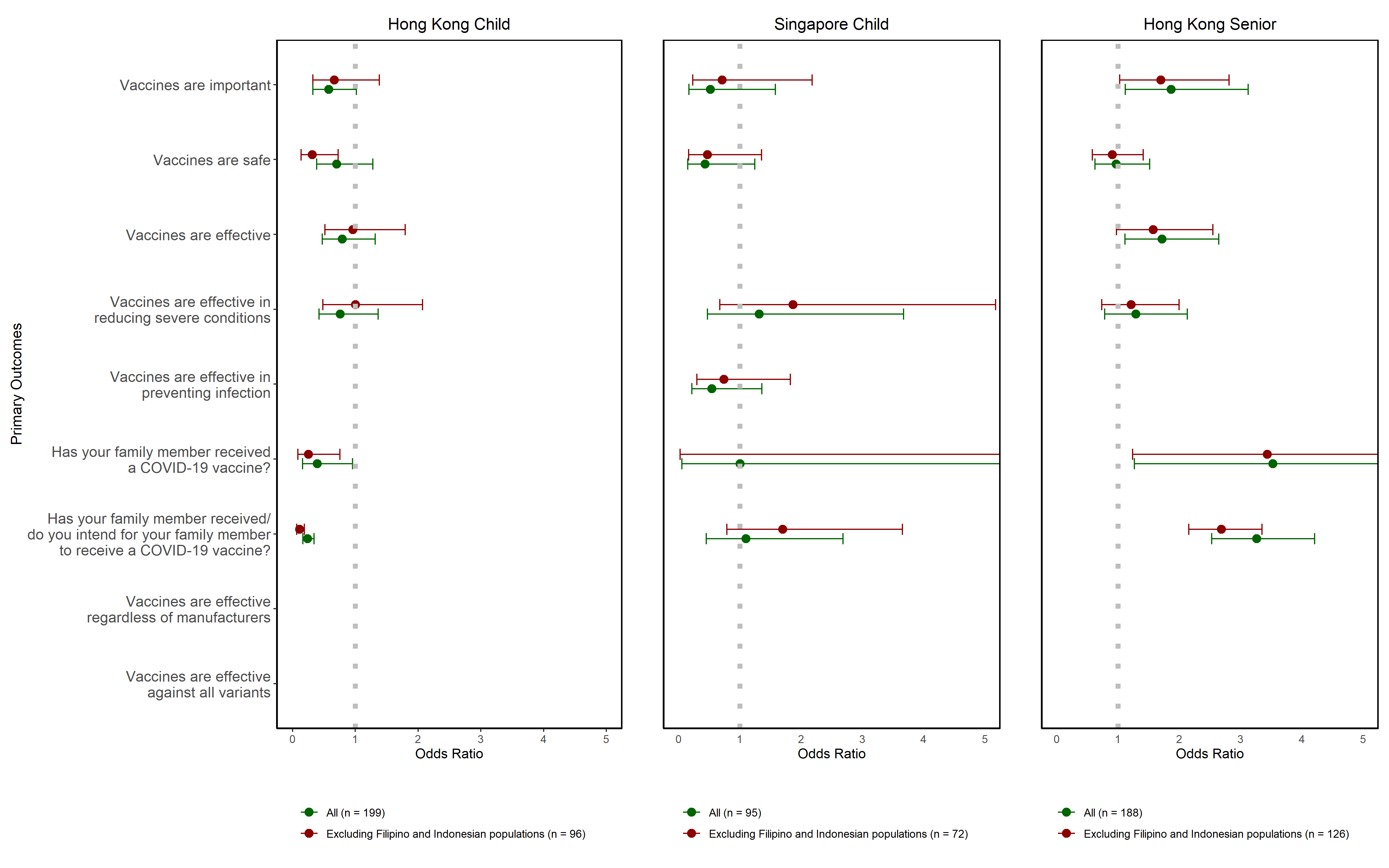
**

Values indicate odds ratios; values above 1 indicate that chatbot intervention was more likely to lead to an improvement in the above indices in comparison to control group with no chatbot intervention, values below 1 indicate that chatbot intervention was less likely to lead to an improvement in the indices. Bars indicate 95% confidence interval calculated with the profile likelihood method. Rows left empty in each study group are variables not covered in the corresponding study group.

**Supplementary Figure 3. Sensitivity analysis for secondary outcomes with and without Filipino and Indonesian subgroup.**
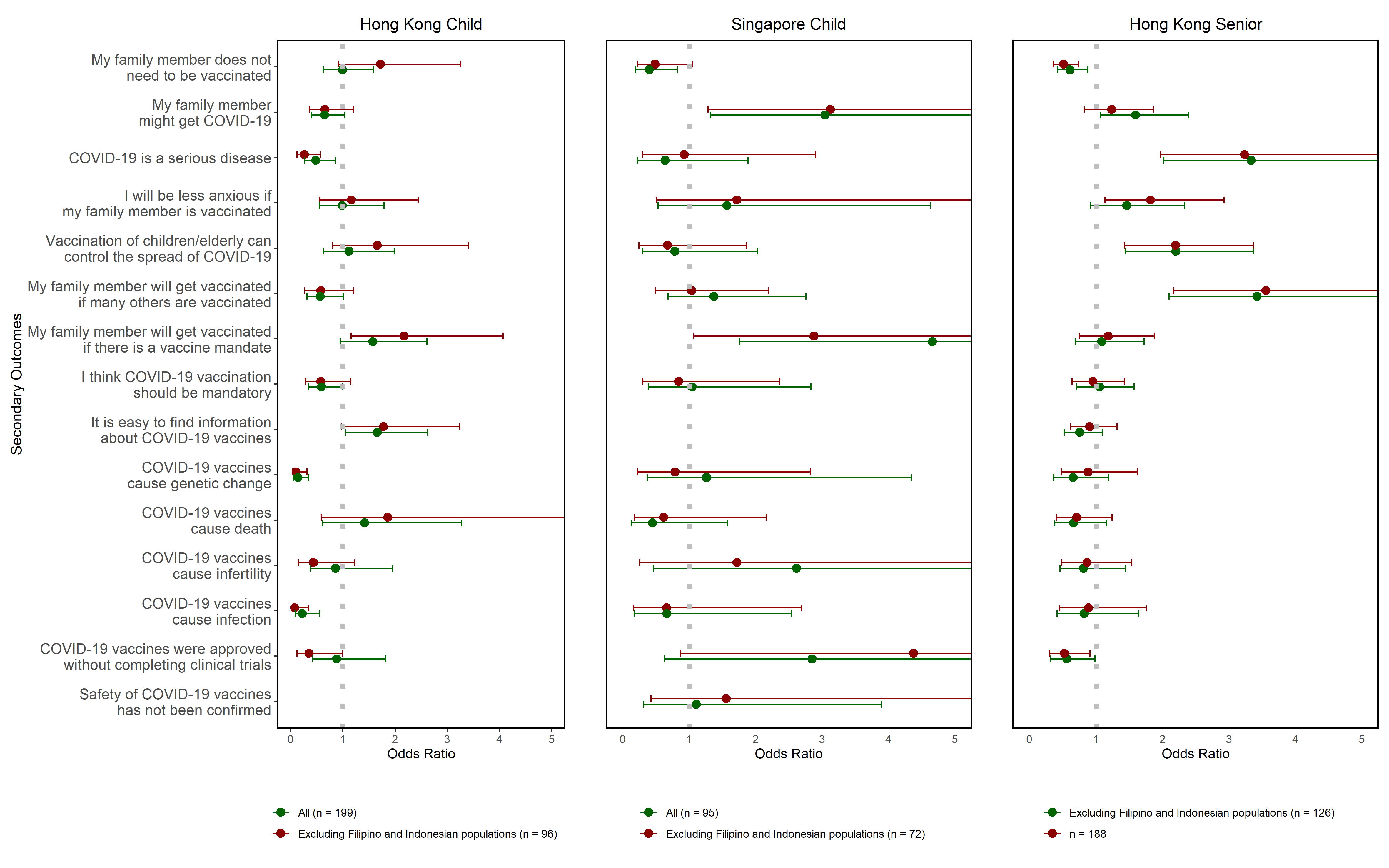


Values indicate odds ratios; values above 1 indicate that chatbot intervention was more likely to lead to an improvement in the above indices in comparison to control group with no chatbot intervention, values below 1 indicate that chatbot intervention was less likely to lead to an improvement in the indices. Bars indicate 95% confidence interval calculated with the profile likelihood method.

**Supplementary Figure 4. Word cloud of English keywords from participant interactions with chatbots in Hong Kong and Singapore.**


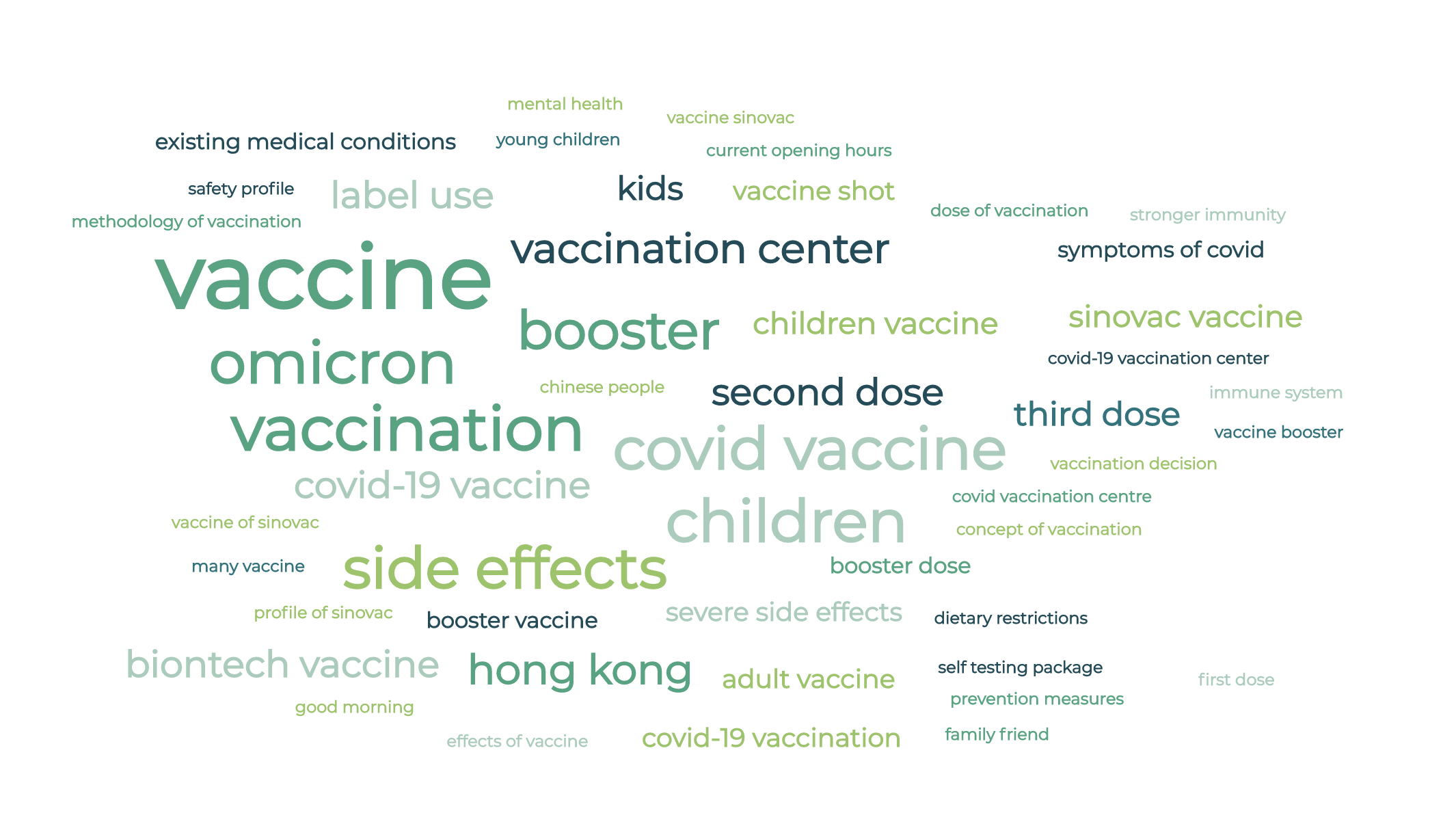


**Supplementary Figure 5. Word cloud of Chinese keywords from participant interactions with chatbots in Hong Kong and Singapore.**


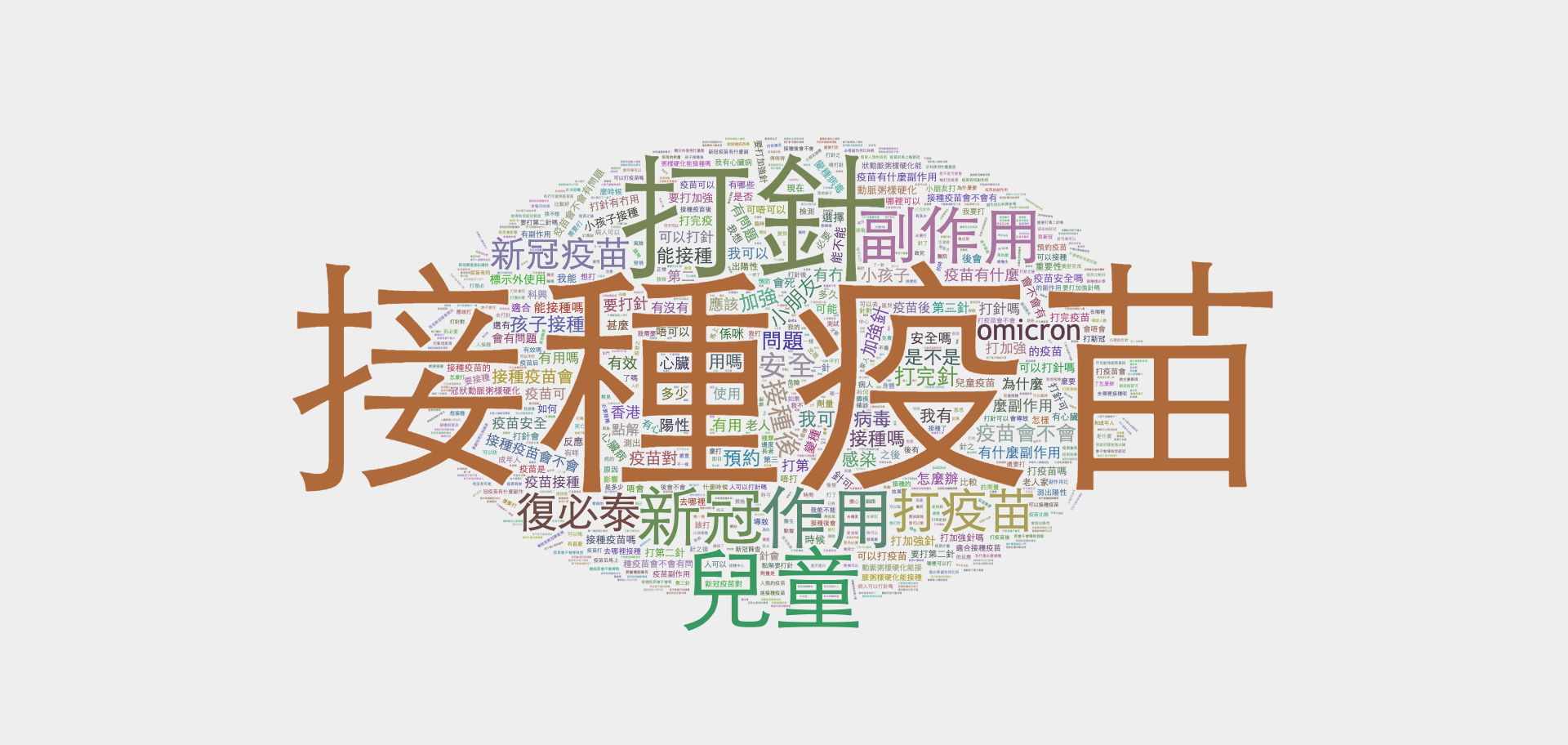


**Supplementary Figure 6. Word cloud of Chinese key phrases from participant interactions with chatbots in Hong Kong and Singapore.**


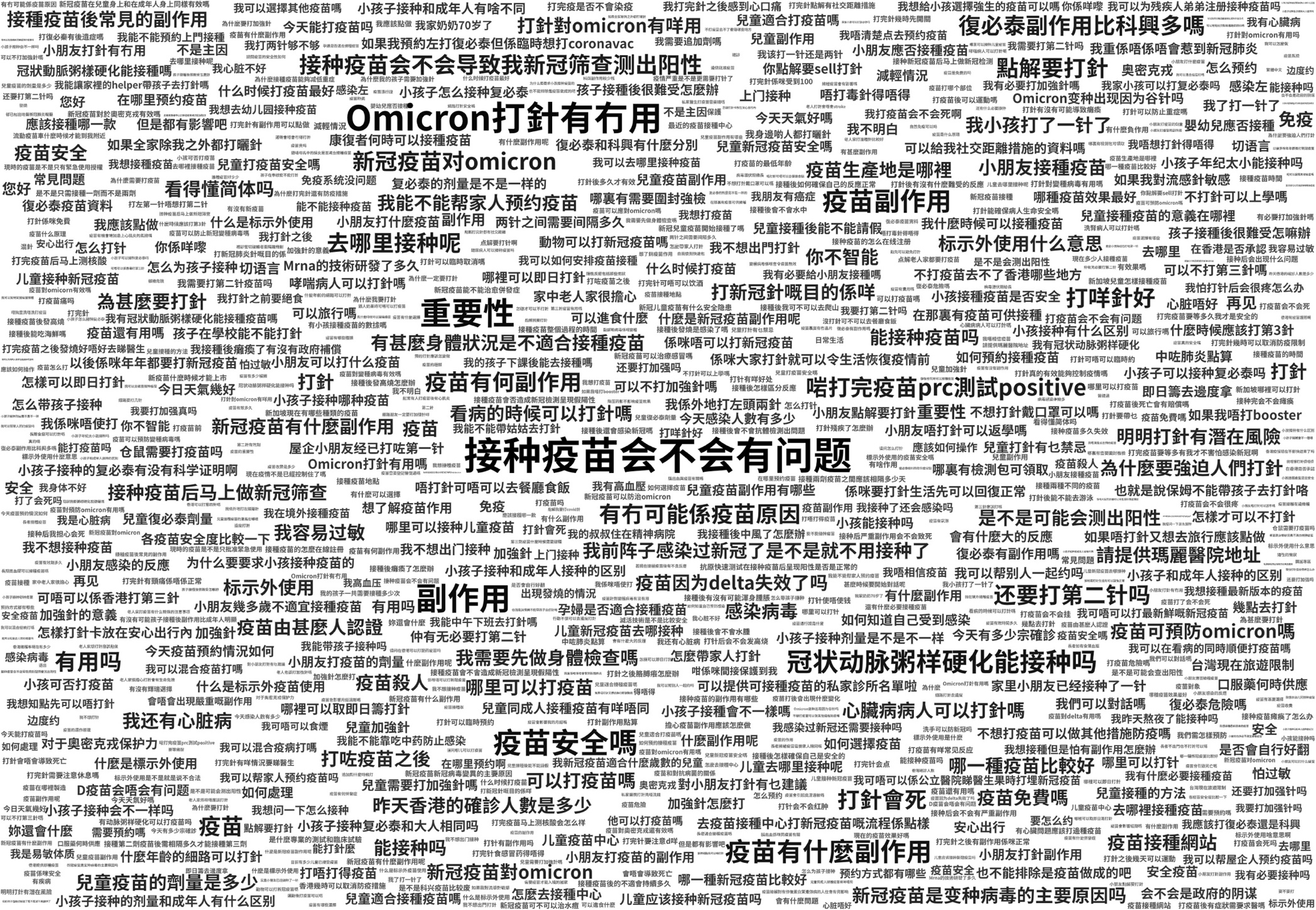


**Supplementary Figure 7. Word cloud of Thai key phrases (with English translation) from Thai participant interactions with ChatSure.**


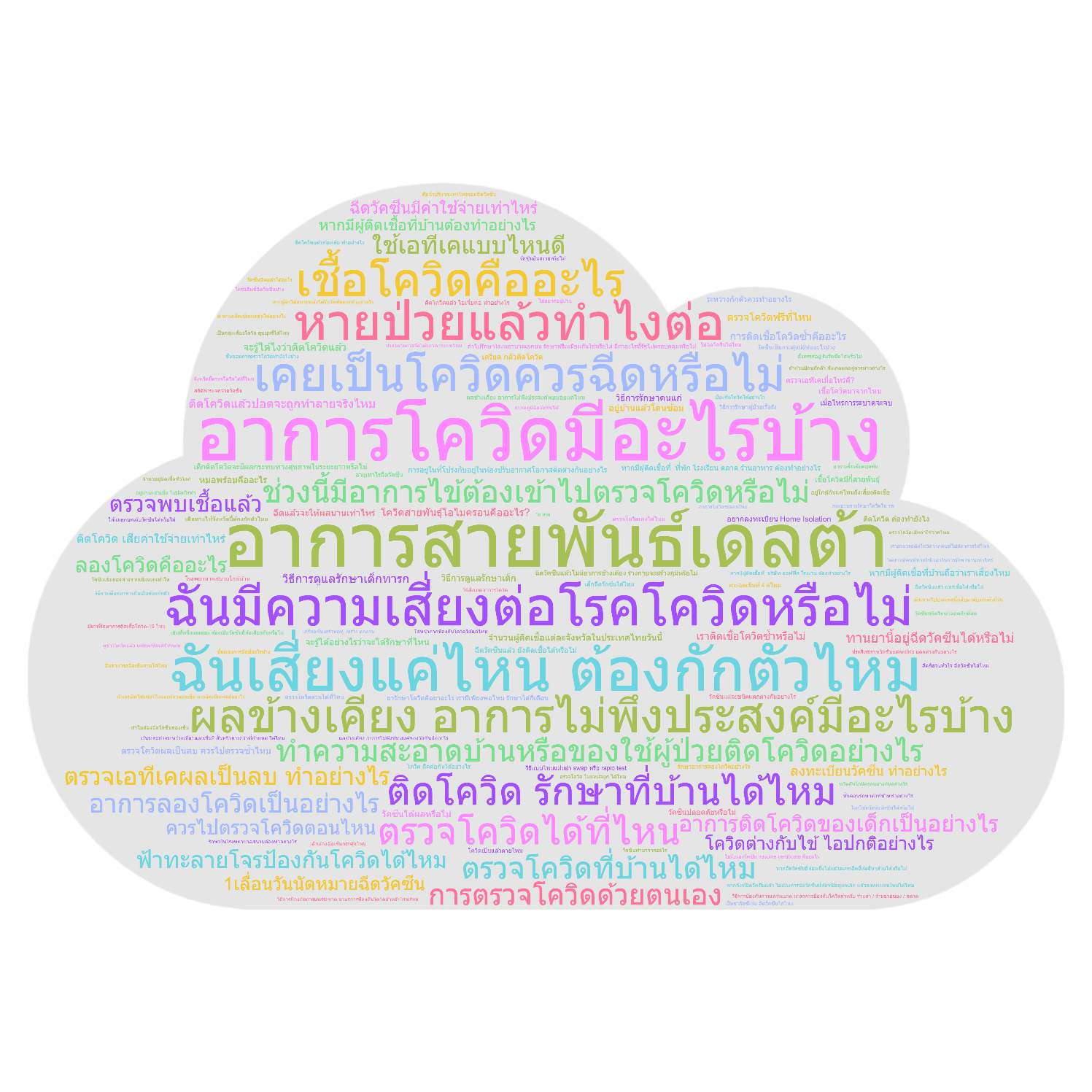
 **
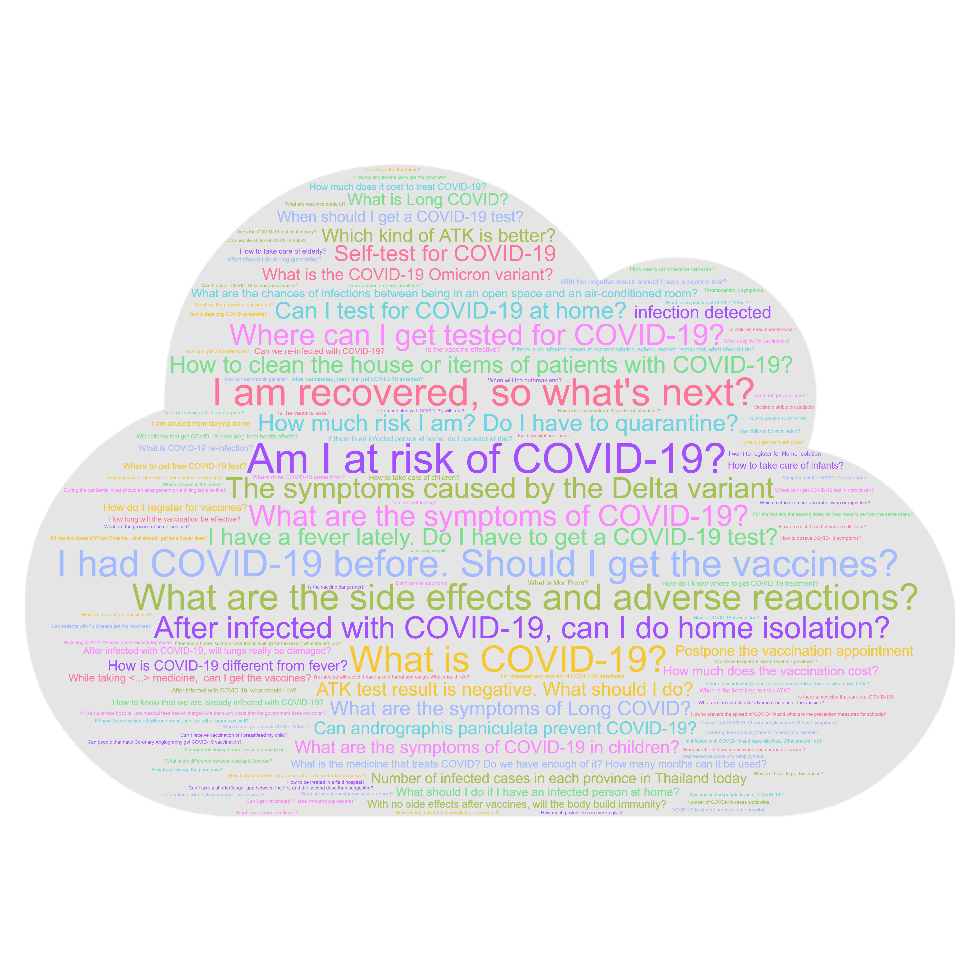
**

**Supplementary Figure 8. Flow diagram of the randomised controlled trial in Thailand, Hong Kong, and Singapore in detail**


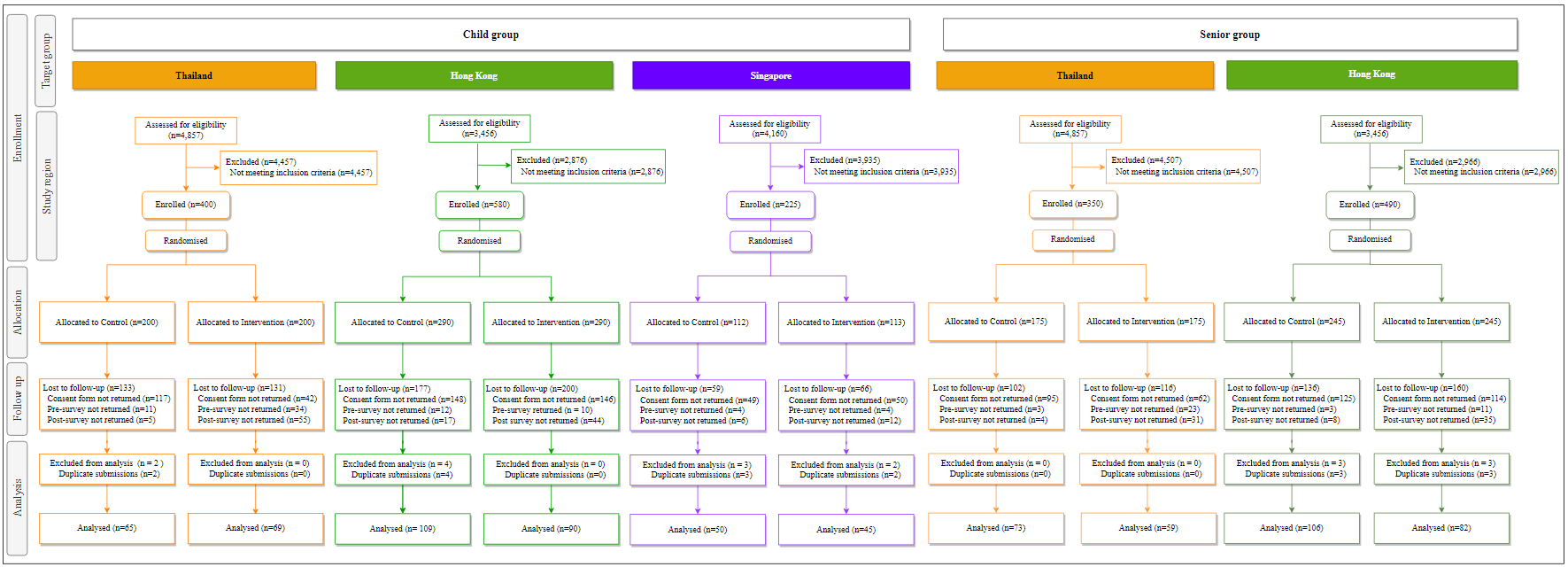


**Supplementary Methods**

**Supplementary Method 1. Confirmatory Factor Analysis (CFA) of the Vaccine Confidence Index (VCI) ^2-4^**

| **VCI Versions** | **Number of Observations** | **GFI** | **AGFI** | **CFI** | **TLI** | **RNI** | **RMSEA** | **SRMR** |
| --- | --- | --- | --- | --- | --- | --- | --- | --- |
| Thai | 532 | 0.832 | 0.609 | 0.824 | 0.707 | 0.824 | 0.287 | 0.098 |
| Chinese  (Hong Kong) | 400 | 0.992 | 0.959 | 0.996 | 0.989 | 0.996 | 0.079 | 0.01 |
| Chinese (Singapore) | 88 | 0.984 | 0.921 | 0.995 | 0.986 | 0.995 | 0.068 | 0.023 |
| English  (Hong Kong) | 372 | 0.975 | 0.874 | 0.981 | 0.942 | 0.981 | 0.158 | 0.025 |
| English (Singapore) | 102 | 0.934 | 0.669 | 0.955 | 0.864 | 0.955 | 0.246 | 0.035 |

CFA was done using the lavaan package in R.^4^;GFI = goodness-of-fit index, ranges from 0 to 1 with larger values indicating better fit; AGFI = approximate goodness of fit index, ranges from 0 to 1 with larger values indicating better fit; CFI = comparative fit index, good fit should be close to 0.95; TLI = Tucker-Lewis index, good fit should be close to 0.95; RNI = relative noncentrality index, good fit should be close to 0.95; RMSEA = root mean square error of approximation, below 0.05 is considered a good fit; SRMR = standardized root mean square residual, below 0.08 is considered acceptable

*Below are items included in each version of the scale, translated versions can be found in the supplementary material, answers were recorded on a five-point Likert scale from “strongly disagree” to “strongly agree”.*

*Thailand:*

- *“Overall, I think COVID-19 vaccines are important for children/elderly”,*
- *“Overall, I think COVID-19 vaccines are safe for children/elderly”,*
- *“Overall, I think COVID-19 vaccines are effective for children/elderly”,*
- *“Overall, I think COVID-19 vaccines are effective in reducing the risk of developing severe conditions”,*
- *“Overall, I believe COVID-19 vaccines are effective for children regardless of the manufacturer of the vaccine”,*
- *“Overall, I believe COVID-19 vaccines are effective for children against all variants of COVID-19”*

*Hong Kong:*

- *“Overall, I think COVID-19 vaccines are important for children/elderly”,*
- *“Overall, I think COVID-19 vaccines are safe for children/elderly”,*
- *“Overall, I think COVID-19 vaccines are effective for children/elderly”,*
- *“Overall, I think COVID-19 vaccines are effective in reducing the risk of developing severe conditions”*

*Singapore:*

- *“Overall, I think COVID-19 vaccines are important for children/elderly”,*
- *“Overall, I think COVID-19 vaccines are safe for children/elderly”,*
- *“Overall, I think COVID-19 vaccines are effective in reducing the risk of developing severe conditions”,*
- *“Overall, I think COVID-19 vaccines are effective at preventing infection”*

**Supplementary Method 2. Study population and primary outcome variables**

| **Study**  **location** | **Number of  target groups** | **Respondent characteristics** | **Details** | **Sample for pilot** | **Analysed sample population** | **Outcomes** |
| --- | --- | --- | --- | --- | --- | --- |
| Thailand | 2 | Parents with no age restriction | Vaccinated or unvaccinated parent of an unvaccinated 5 to 11 year old | 40:  - 20 control,  - 20 intervention | 134:  - 65 control  - 69 intervention through ChatSure Messenger chatbot | 1) Vaccine Acceptance   - “Has your child received/do you intend for your child to receive a COVID-19 vaccine?”   2) Vaccine Confidence  - Importance   - “Overall, I think COVID-19 vaccines are important for children/elderly."   - Effectiveness   - “Overall, I think COVID-19 vaccines are effective for children/elderly.” - “Overall, I think COVID-19 vaccines are effective in reducing the risk of developing severe conditions." - “Overall, I believe COVID-19 vaccines are effective for children regardless of the manufacturer of the vaccine.” - “Overall, I believe COVID-19 vaccines are effective for children against all variants of COVID-19.”   - Safety   - “Overall, I think COVID-19 vaccines are safe for children/elderly.” |
|  |  | Guardians who are 18+ years old | Vaccinated or unvaccinated son/daughter/grandchild of an unvaccinated 60+ year old | 40:  - 20 control,  - 20 intervention | 132:  - 73 control  - 59 intervention through ChatSure Messenger chatbot |  |
| Hong Kong | 2 | Parents with no age restriction | Vaccinated or unvaccinated parent of an unvaccinated child under 18 years old | 40:  - 20 control,  - 20 intervention | 199:  - 109 control  - 90 intervention through D^2^4H WhatsApp chatbot | 1) Vaccine Acceptance   - “Has your child received/do you intend for your child to receive a COVID-19 vaccine?”   2) Vaccine Confidence  - Importance   - “Overall, I think COVID-19 vaccines are important for children/elderly."   - Effectiveness   - “Overall, I think COVID-19 vaccines are effective for children/elderly.” - “Overall, I think COVID-19 vaccines are effective in reducing the risk of developing severe conditions."   - Safety   - “Overall, I think COVID-19 vaccines are safe for children/elderly.” |
|  |  | Guardians who are 18+ years old | Vaccinated or unvaccinated son/daughter/grandchild of an unvaccinated 60+ year old | 40:  - 20 control,  - 20 intervention | 188:  - 106 control  - 82 intervention through D^2^4H WhatsApp chatbot |  |
| Singapore | 1 | Parents with no age restriction | Vaccinated or unvaccinated parent of an unvaccinated 5 to 11 year old | 40:  - 20 control,  - 20 intervention | 95:  - 50 control  - 45 intervention through D^2^4H WhatsApp chatbot | 1) Vaccine Acceptance   - “Has your child received/do you intend for your child to receive a COVID-19 vaccine?”   2) Vaccine Confidence  - Importance   - “Overall, I think COVID-19 vaccines are important for children/elderly."   - Effectiveness   - "Overall, I think COVID-19 vaccines are effective in reducing the risk of developing severe conditions." - “Overall, I think COVID-19 vaccines are effective at preventing infection.”   - Safety   - “Overall, I think COVID-19 vaccines are safe for children/elderly." |

**Supplementary Method 3. ChatSure link used as an intervention in Thailand:** [**https://www.facebook.com/socialmarketingth**](https://www.facebook.com/socialmarketingth)


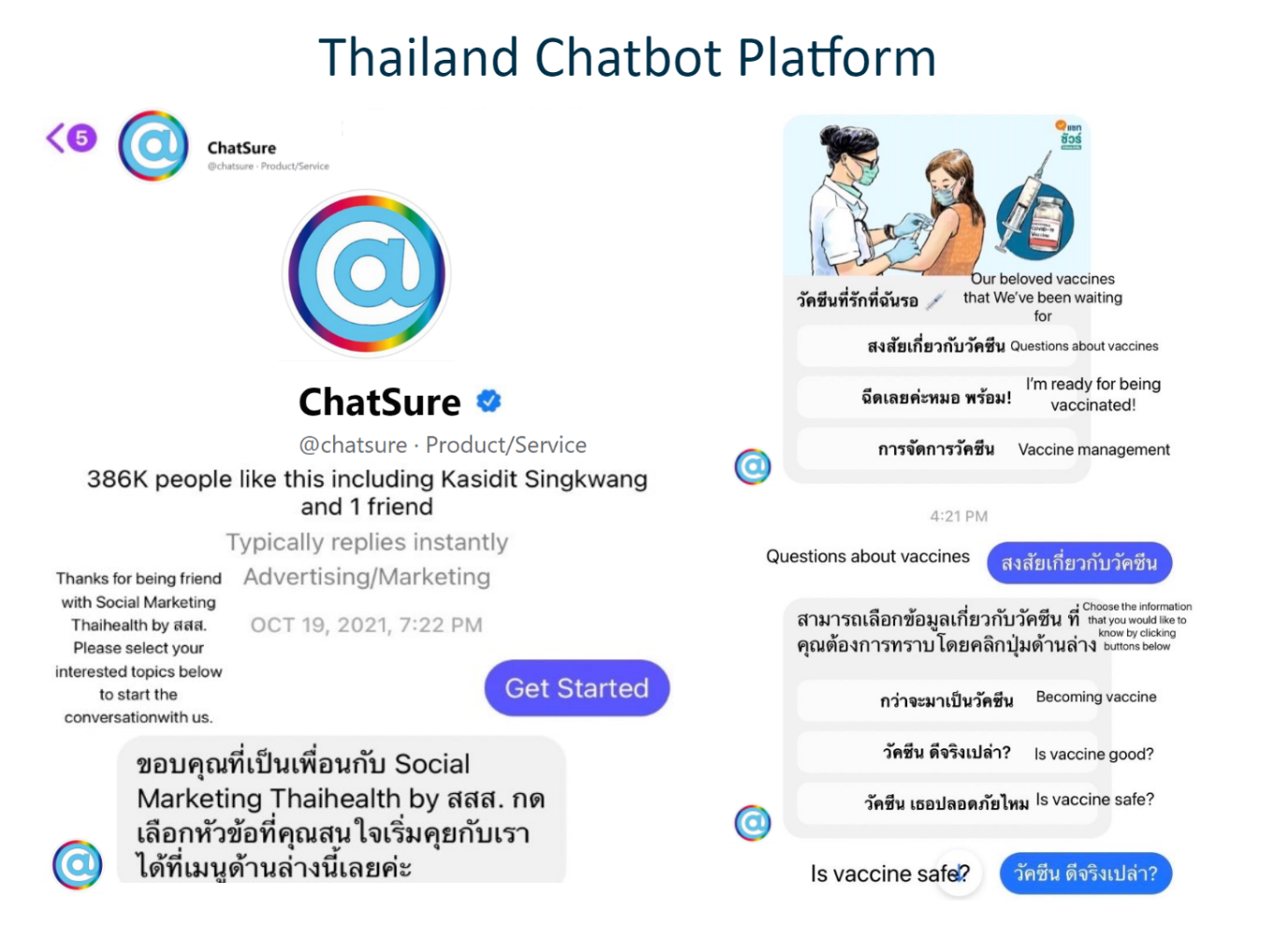


**Supplementary Method 4. D24H Chatbot link used as an intervention in Hong Kong and Singapore:** [**https://wa.me/85267444704?text=hi**](https://wa.me/85267444704?text=hi)

**
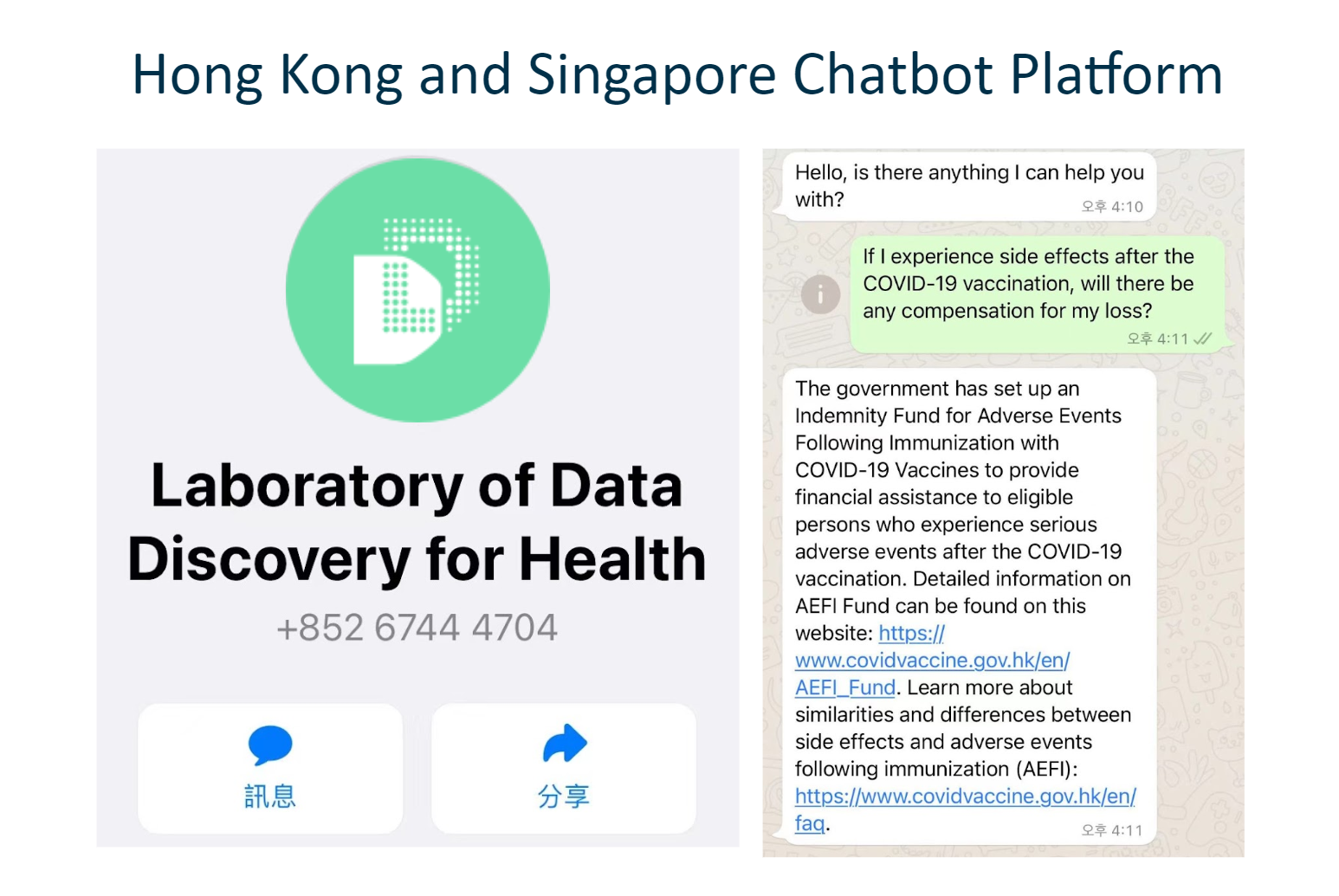
**

**Supplementary Method 5. English Questionnaire used in Thailand for both the control and intervention groups.**

| Task title | Pre-questionnaire – Senior group (control/intervention) |
| --- | --- |
| Target locations | Thailand |
| Submission goal | 200 control / 200 intervention |

| # | Conditional logic | Question | Question type | Hint | Response options |
| --- | --- | --- | --- | --- | --- |
| 1 |  | We appreciate your participation in this conversational COVID19 chatbot evaluation. This is the first out of three tasks that you will need to complete. This survey will ask you some questions with regards to your elderly parents//grandparents (60 years old or above).  Before we begin the survey, please tell us about yourself: | Select one |  | I understand, continue. |
| 2 |  | Please tell us which of the following statements best describe YOUR current COVID-19 vaccination status: | Select one |  | - I have not been vaccinated, and I do not plan to get it anytime soon - I have not been vaccinated, but I plan to get vaccinated soon - I have received 1 dose - I have received 2 doses - I have received 3 doses |
| 3 | If ‘I have not been vaccinated, and I do not plan to get it anytime soon’ OR I have not been vaccinated, but I plan to get vaccinated soon’ in Q2 | Have you registered to get a COVID-19 vaccine? | Select one |  | - Yes - No |
| 4 |  | Are you currently working in a healthcare setting? | Select one |  | - Yes, as a medical doctor - Yes, as a health professional other than a medical doctor - No |
| 5 |  | Which of the following describes your legal status in Thailand? | Select one |  | - Citizen - Resident - Prefer not to say |
| 6 |  | What province do you currently live in? | Select one |  | - Bangkok Metropolis - Nakhon Pathom - Pathum Thani - Nonthaburi - Samut Prakan - Samut Sakhon |
| 7 |  | Now we would like to ask some background questions about ONE elderly parent/grandparent. If both of your parents/grandparents are around and are 60 years old or above, please select the parent/grandparent who had the most recent birthday: | Select one |  | - I understand, continue. |
| 8 |  | What is the vaccination status of your family member as of now? | Select one |  | - My elderly family member is vaccinated with at least the first dose of the vaccine - My elderly family member has made an appointment but has not been vaccinated with the first dose yet - My elderly family member has not made an appointment but will get the vaccine. - My elderly family member has no plan |
|  |  |  |  |  | to get vaccinated anytime soon. |
| 9 | If you choose “My elderly family member has not made an appointment but will get  the vaccine.” in Q8 | When will your family member receive the first dose? | Date |  |  |
| 10 |  | What is the relationship between you and the elderly parent/grandparent you are answering for in this survey? | Select one |  | - Father - Mother - Grandfather - Grandmother |
| 11 |  | How old is your elderly family member? | Select one |  | - Between 60 to 70 years old - Between 71 to 80 years old - Between 81 and 90 years old - Older than 91 years old |
| 12 |  | Does your elderly family member have any of the underlying medical conditions which could increase their risk of severe illness from COVID- 19? | Select many | Please select all that apply. | - Chronic disease (respiratory/kidney/h eart/liver) - Diabetes - Blood disease - Disease with an immune suppression - Receiving treatment that may suppress the immune system - Neurological or neuromuscular disease due to immune deficiency - Physical decline associated with a neurological disease or a neuromuscular disease - Chromosomal abnormality - Severe psychosomatic disorder - Depression/anxiety disorder |
|  |  |  |  |  | - Sleep Apnea Syndrome - Obesity - None of the above - Other |
| 13 |  | Does your elderly family member normally receive the influenza vaccine? | Select one |  | - Yes - No - Unsure |
| 14 |  | Has your elderly family member ever been diagnosed (tested positive or diagnosed by doctor) with COVID- 19? | Select one |  | - Yes - No - Unsure |
| 15 |  | Has your elderly family member ever experienced a side effect or allergy after any vaccination? | Select one |  | - Yes - No - Unsure |
| 16 |  | "Overall, I think COVID-19 vaccines are IMPORTANT for the elderly." | Select one | Please rate your level of agreement to the following statement. | - Strongly disagree - Tend to disagree - Neither disagree nor agree - Tend to agree - Strongly agree |
| 17 |  | "Overall, I think COVID-19 vaccines are SAFE for the elderly." | Select one | Please rate your level of agreement to the following statement. | - Strongly disagree - Tend to disagree - Neither disagree nor agree - Tend to agree - Strongly agree |
| 18 |  | "Overall, I think COVID-19 vaccines  are EFFECTIVE for the elderly." | Select one | Please rate your level of agreement to the following statement. | - Strongly disagree - Tend to disagree - Neither disagree nor agree - Tend to agree - Strongly agree |
| 19 |  | "Vaccination of elderly people is compatible with my religious/cultural beliefs." | Select one | Please rate your level of agreement to the following statement. | - Strongly disagree - Tend to disagree - Neither disagree nor agree - Tend to agree - Strongly agree |
| 20 |  | “I believe COVID-19 vaccines are effective for the elderly regardless of the manufacturer of the vaccine” | Select one | Please rate your level of agreement to the following statement. | - Strongly disagree - Tend to disagree - Neither disagree nor agree - Tend to agree - Strongly agree |
| 21 |  | “I believe COVID-19 vaccines are effective for the elderly against all variants of COVID- 19” | Select one | Please rate your level of agreement to the following statement. | - Strongly disagree - Tend to disagree - Neither disagree nor agree - Tend to agree - Strongly agree |
| 22 |  | Do you want your elderly family member vaccinated against COVID-19 if  vaccination is indicated and available for them? | Select one | Please rate your level of agreement to the following statement. | - Yes, definitely - Unsure, but leaning towards yes - Unsure, but leaning towards no - No, definitely not |
| 23 | If Unsure but leaning towards yes OR Unsure but leaning towards no OR No,  definitely not in Q20 | From the following list, please select whose recommendation might convince you to want your elderly family member vaccinated against COVID-19. | Select many |  | - A friend - A parent - A teacher - A politician I like - A celebrity I like - The Government - A doctor or healthcare worker - Other - I don’t know |
| 24 | If Unsure, but leaning towards yes OR Unsure, but leaning towards no OR No,  definitely not in Q20 | You indicated that you are either unsure or reluctant to the idea of your elder family member receiving a COVID19 vaccine.  Please briefly explain why. | Text |  |  |
| 25 | If Unsure, but leaning towards yes OR Unsure, but leaning towards no OR No,  definitely not in Q20 | Please tell us briefly what could change your mind about how you feel with regards to your elder family member receiving a COVID19 vaccine in the future. | Text |  |  |
| 26 |  | “My elderly family member may get COVID-19 infection within the next 6 month.” | Select one | Please rate your level of agreement to the following statement. | - Strongly disagree - Tend to disagree - Neither disagree nor agree - Tend to agree - Strongly agree |
| 27 |  | “COVID-19 is a serious disease” | Select one | Please rate your level of agreement to the following statement. | - Strongly disagree - Tend to disagree - Neither disagree nor agree - Tend to agree - Strongly agree |
| 28 |  | “Overall, I think COVID-19 vaccines are effective in reducing the risk of developing severe conditions.” | Select one | Please rate your level of agreement to the following statement. | - Strongly disagree - Tend to disagree - Neither disagree nor agree - Tend to agree - Strongly agree |
| 29 |  | “I believe I will be less anxious about my elderly family member chance of contracting the COVID-19, if  he/she is vaccinated” | Select one | Please rate your level of agreement to the following statement. | - Strongly disagree - Tend to disagree - Neither disagree nor agree - Tend to agree - Strongly agree |
| 30 |  | “My elderly family member does not need to take a COVID-19 vaccine because they practise social distancing and wash hands frequently with soap or sanitizer, which can help prevent the spread of COVID- 19” | Select one | Please rate your level of agreement to the following statement. | - Strongly disagree - Tend to disagree - Neither disagree nor agree - Tend to agree - Strongly agree |
| 31 |  | “I believe vaccination of senior citizens can help control the spread of COVID-19” | Select one | Please rate your level of agreement to the following statement. | - Strongly disagree - Tend to disagree - Neither disagree nor agree - Tend to agree - Strongly agree |
| 32 |  | “It is easy for my elderly family member to find relevant information on COVID- 19 vaccines” | Select one | Please rate your level of agreement to the following statement. | - Strongly disagree - Tend to disagree - Neither disagree nor agree |
|  |  |  |  |  | - Tend to agree - Strongly agree |
| 33 |  | “I will want my elderly family member to take a COVID-19 vaccine if many others have  taken it” | Select one | Please rate your level of agreement to the following statement. | - Strongly disagree - Tend to disagree - Neither disagree nor agree - Tend to agree - Strongly agree |
| 34 |  | “I will want my elderly family member to take a COVID-19 vaccine if a COVID-19 vaccine certificate or passport is required for work, travel, social events or dine-in” | Select one | Please rate your level of agreement to the following statement. | - Strongly disagree - Tend to disagree - Neither disagree nor agree - Tend to agree - Strongly agree |
| 35 |  | “COVID-19 vaccination should be compulsory for all senior citizens in Thailand” | Select one | Please rate your level of agreement to the following statement. | - Strongly disagree - Tend to disagree - Neither disagree nor agree - Tend to agree - Strongly agree |
| 36 |  | “I think the government and health authorities are acting in the interest of public  health” | Select one | Please rate your level of agreement to the following statement. | - Strongly disagree - Tend to disagree - Neither disagree nor agree - Tend to agree - Strongly agree |
| 37 |  | In the past month, on average, how much time per day have you spent actively using social media? | Select one |  | - I’m not a social media user - Less than 10 minutes per day - 11–30 minutes per day - 31–60 minutes per day - 1--2 hours per day - 2–3 hours per day - More than 3 hours per day |
| 38 |  | In the past month, from which of these social media platforms did you receive information regarding COVID-19 vaccines? | Select many | Please select all that apply. | - Facebook - Twitter - YouTube - WhatsApp - LINE |
|  |  |  |  |  | - Facebook Messenger - Instagram - TikTok - LinkedIn - Other |
| 39 |  | From the following list, please select the source that you use and trust the MOST to learn about COVID-19 vaccines. | Select one |  | - Local public television (e.g. NBT2HD, ThaiPBS) - Radio - International television (e.g. CNN, BBC) - Newspapers or magazines - Social media (e.g. Line, Facebook, Twitter, WhatsApp, etc.) - Local public health authorities, (e.g. the Ministry of Public Health, Department of Health Promotion) - Healthcare workers - Religious leaders - International health authorities (such as the World Health Organisation) - Community organisations - Scientists - Government websites - The internet or search engines (e.g. Google, Yahoo, etc.) - Family and friends - Work, school, or college/university - I don’t know - Other |
| 40 |  | Have you ever seen or heard any COVID-19 associated information on social media which might have persuaded you NOT to immunise your elderly family member against COVID-19? | Select one |  | - Yes - No - I don’t know |
| 41 | If Yes in Q38 | Please describe what COVID-19 associated information on social | Text |  |  |
|  |  | media persuaded you NOT to immunise your elderly family member against COVID-19. |  |  |  |
| 42 |  | Up next we will show you a set of statements and you will have to indicate if you think they are true, false or if you are unsure. | Select one |  | - I understand, continue. |
| 43 |  | “Many people have died after getting the COVID-19 vaccines” | Select one |  | - True - Unsure - False |
| 44 |  | “COVID-19 vaccination is associated with infertility and/or miscarriage” | Select one |  | - True - Unsure - False |
| 45 |  | “COVID-19 vaccines were approved without completing the normal process of the clinical trial” | Select one |  | - True - Unsure - False |
| 46 |  | Thank you for completing the first task of this study - the baseline survey. Next, please be reminded to complete the following to qualify for the post- intervention survey about chatbot user experience.  In the next week, please 1) use the chatbot for more than ten minutes; 2) ensure that you have more than ten conversations with the chatbot.  Participants who meet the above requirements and complete the post- survey within a week will receive the rewards as a “thank you” for fulfilling the tasks. (After verification, those who were unable to complete the tasks within the designated |  |  | - I understand, and will start using the ‘Chat Sure' Messenger chatbot. |
|  |  | time will not be eligible for compensation). Thank you very much for your cooperation. |  |  |  |

| Task title | Post-questionnaire – Senior group (control/intervention) |
| --- | --- |
| Target locations | Thailand |
| Submission goal | 200 control / 200 intervention |

| # | Conditional logic | Question | Question type | Hint | Response options |
| --- | --- | --- | --- | --- | --- |
| 1 |  | We appreciate your participation in this conversational COVID19 chatbot evaluation. This is the third out of three tasks that you will need to complete. | Select one |  | - I understand, continue |
| 2 |  | Before we begin, could you please tell us which of the following statements best describe YOUR current COVID-19 vaccination status | Select one |  | - I have not been vaccinated, and I do not plan to get it anytime soon - I have not been vaccinated, but I plan to get vaccinated soon - I have received 1 dose - I have received 2 doses - I have received 3 doses |
| 3 |  | Now we would like to follow up on questions about your elderly family member for whom you completed the previous survey.  Has your elderly family member received at least one dose of the COVID-19 vaccine? | Select one |  | - Yes - No |
| 4 | If Yes in Q3 | Please briefly tell us why you decided to get your elderly family member vaccinated. | Text |  |  |
| 5 | If No in Q3 | Do you want your elderly family member to receive a COVID-19 vaccine in the future if vaccination is indicated and available for them? | Select one |  | - Yes, definitely - Unsure, but leaning towards yes - Unsure, but leaning towards no - No, definitely not |
| 6 | If Yes definitely in Q5 | Please briefly tell us why you want your elderly family member to receive a COVID-19 vaccine. | Text |  |  |
| 7 | If any response BUT Yes definitely in Q5 | Please briefly tell us why you are hesitant or reluctant to the idea of your elder family member receiving a COVID19 vaccine. | Text |  |  |
| 8 |  | Have you personally encouraged any of the following to take a COVID-19 vaccine? | Select many |  | - Spouse - Parent - Sibling - Son or daughter - Friend - Colleague - Other - I have not encouraged anyone to take a COVID-19 vaccine |
| 9 |  | Would you be willing for your elderly family member to receive an annual booster vaccine against COVID-19 if it is recommended and available for them? | Select one |  | - Yes, definitely - Unsure, but leaning towards yes - Unsure, but leaning towards no - No, definitely not |
| 10 |  | "Overall, I think COVID-19 vaccines are IMPORTANT for the elderly." | Select one | Please rate your level of agreement to the following statement. | - Strongly agree - Tend to agree - Neither agree nor disagree - Tend to disagree - Strongly disagree |
| 11 |  | "Overall, I think COVID-19 vaccines are SAFE for the elderly." | Select one | Please rate your level of agreement to the following statement. | - Strongly agree - Tend to agree - Neither agree nor disagree - Tend to disagree - Strongly disagree |
| 12 |  | "Overall, I think COVID-19 vaccines  are EFFECTIVE for the elderly." | Select one | Please rate your level of agreement to the following statement. | - Strongly agree - Tend to agree - Neither agree nor disagree - Tend to disagree - Strongly disagree |
| 13 |  | "Vaccination of elderly people is compatible with my religious/cultural beliefs." | Select one | Please rate your level of agreement to the following statement. | - Strongly agree - Tend to agree - Neither agree nor disagree - Tend to disagree - Strongly disagree |
| 14 |  | “I believe COVID-19 vaccines are effective for the elderly regardless of the manufacturer of the vaccine” | Select one | Please rate your level of agreement to the following statement. | - Strongly disagree - Tend to disagree - Neither disagree nor agree - Tend to agree - Strongly agree |
| 15 |  | “I believe COVID-19 vaccines are effective for the elderly against all variants of COVID- 19” | Select one | Please rate your level of agreement to the following statement. | - Strongly disagree - Tend to disagree - Neither disagree nor agree - Tend to agree - Strongly agree |
| 16 |  | “My elderly family member may get COVID-19 infection within the next 6 month.” | Select one | Please rate your level of agreement to the following statement. | - Strongly agree - Agree - Neither agree nor disagree - Disagree - Strongly disagree |
| 17 |  | “COVID-19 is a serious disease” | Select one | Please rate your level of agreement to the following statement. | - Strongly agree - Agree - Neither agree nor disagree - Disagree - Strongly disagree |
| 18 |  | “Overall, I think COVID-19 vaccines are effective in reducing the risk of developing severe conditions.” | Select one | Please rate your level of agreement to the following statement. | - Strongly agree - Agree - Neither agree nor disagree - Disagree |
|  |  |  |  |  | - Strongly disagree |
| 19 |  | “I believe I will be less anxious about my elderly family member chance of contracting the COVID-19, if  he/she is vaccinated” | Select one | Please rate your level of agreement to the following statement. | - Strongly agree - Agree - Neither agree nor disagree - Disagree - Strongly disagree |
| 20 |  | “My elderly family member does not need to take a COVID-19 vaccine because they practise social distancing and wash hands frequently with soap or sanitizer, which can help prevent the spread of COVID- 19” | Select one | Please rate your level of agreement to the following statement. | - Strongly agree - Agree - Neither agree nor disagree - Disagree - Strongly disagree |
| 21 |  | “I believe vaccination of senior citizens can help control the spread of COVID-19” | Select one | Please rate your level of agreement to the following statement. | - Strongly agree - Agree - Neither agree nor disagree - Disagree - Strongly disagree |
| 22 |  | “It is easy for my elderly family member to find relevant information on COVID- 19 vaccines” | Select one | Please rate your level of agreement to the following statement. | - Strongly agree - Agree - Neither agree nor disagree - Disagree - Strongly disagree |
| 23 |  | “I will want my elderly family member to take a COVID-19 vaccine if many others have  taken it” | Select one | Please rate your level of agreement to the following statement. | - Strongly agree - Agree - Neither agree nor disagree - Disagree - Strongly disagree |
| 24 |  | “I will want my elderly family member to take a COVID-19 vaccine if a COVID-19 vaccine certificate or passport is required for work, travel, social events or dine-in” | Select one | Please rate your level of agreement to the following statement. | - Strongly agree - Agree - Neither agree nor disagree - Disagree - Strongly disagree |
| 25 |  | “COVID-19 vaccination should be compulsory for all senior citizens in Thailand” | Select one | Please rate your level of agreement to the following statement. | - Strongly agree - Agree - Neither agree nor disagree - Disagree - Strongly disagree |
| 26 |  | “I think the government and health authorities are acting in the interest of public  health” | Select one | Please rate your level of agreement to the following statement. | - Strongly disagree - Tend to disagree - Neither disagree nor agree - Tend to agree - Strongly agree |
| 27 |  | Have you ever seen or heard any COVID-19 associated information on social media which might have persuaded you NOT to immunise your elderly family member against COVID-19? | Select one |  | - Yes - No - I don’t know |
| 28 | If Yes in Q26 | Please describe what COVID-19 associated information on social media persuaded you NOT to immunise your elderly family member against COVID-19. | Text |  |  |
| 29 |  | Up next we will show you a set of statements and you will have to indicate if you think they are true, false or if you are unsure. | Select one |  | - I understand, continue. |
| 30 |  | “Many people have died after getting the COVID-19 vaccines” | Select one |  | - True - Unsure - False |
| 31 |  | “COVID-19 vaccination is associated with infertility and/or miscarriage” | Select one |  | - True - Unsure - False |
| 32 |  | “COVID-19 vaccines were approved without completing the normal process of the clinical trial” | Select one |  | - True - Unsure - False |
| 33 |  | Up next we will ask you to rate your level of agreement towards statements about your experience with the chatbot. | Select one |  | - I understand, continue. |
| 34 |  | In the past week, how much time in total have you spent on the chatbot? [Best estimate] | Select one |  | - I did not use the chatbot - Less than 10 minutes - 11-30 minutes - 31–60 minutes - 1-2 hours - 2-3 hours - More than 3 hours |
| 35 |  | “I found the information I was searching for” | Select one |  | - Strongly agree - Agree - Neither agree nor disagree - Disagree - Strongly disagree |
| 36 |  | What types of COVID19 related information were you seeking? | Select many | Please select all that apply. | - Vaccine safety - Vaccine effectiveness - Vaccine appointments - Vaccine centre locations - Vaccine boosters - Vaccines and pregnancy - Other |
| 37 | If Other in Q35 | What other types of information were you seeking? | Text |  |  |
| 38 |  | “The chatbot had a quick response time to my question(s)” | Select one |  | - Strongly agree - Agree - Neither agree nor disagree - Disagree - Strongly disagree |
| 39 |  | “I intend to use the chatbot again” | Select one |  | - Strongly agree - Agree - Neither agree nor disagree - Disagree |
|  |  |  |  |  | - Strongly disagree |
| 40 |  | “I like the design of the chatbot (font, colour, etc.)” | Select one |  | - Strongly agree - Agree - Neither agree nor disagree - Disagree - Strongly disagree |
| 41 |  | “I intend to recommend the chatbot to my  friends and family.” | Select one |  | - Strongly agree - Agree - Neither agree nor disagree - Disagree - Strongly disagree |
| 42 |  | Where do you think this chatbot should be offered? | Select many | Please select all that apply. | - Ministry of Public Health website - Personal patient portal at Hospital Authority - Social media (e.g., Line, WhatsApp, Facebook, Twitter) - Other |
| 43 | If Other in Q41 | Where else do you think the chatbot should be offered? | Text |  |  |
| 44 |  | How could the chatbot be improved? | Select many | Please select all that apply. | - Quicker responses - More detailed information - Change in style of chatbot (e.g. font, colour, size, etc.) - Other |
| 45 | If Other in Q43 | What else could be done to improve the chatbot? | Text |  |  |

| Task title | Pre-questionnaire – Child group (control/intervention) |
| --- | --- |
| Target locations | Thailand |
| Submission goal | 200 control / 200 intervention |

| # | | Conditional logic | | Question | | Question type | | Hint | | Response options | |
| --- | --- | --- | --- | --- | --- | --- | --- | --- | --- | --- | --- |
| 1 | |  | | We appreciate your participation in this conversational COVID19 chatbot evaluation. This is the first out of three tasks that you will need to complete. This survey will ask you some questions with regards to the younger members of your family.  Before we begin the survey, please tell us about yourself: | | Select one | |  | | - I understand, continue. | |
| 2 | |  | | Please tell us which of the following statements best describe YOUR current COVID-19 vaccination status | | Select one | |  | | - I have not been vaccinated, and I do not plan to get it anytime soon - I have not been vaccinated, but I plan to get vaccinated soon - I have received 1 dose - I have received 2 doses - I have received 3 doses | |
| 3 | | If ‘I have not been vaccinated, and I do not plan to get it anytime soon’ OR I have not been vaccinated, but I plan to get vaccinated soon’ in Q2 | | Have you registered to get a COVID-19 vaccine? | | Select one | |  | | - Yes - No | |
| 4 | |  | | Are you currently working in a healthcare setting? | | Select one | |  | | - Yes, as a medical doctor - Yes, as a health professional other than a medical doctor - No | |
| 5 | |  | | What province do you currently live in? | | Select one | |  | | - Bangkok Metropolis - Nakhon Pathom | |
|  | |  | |  | |  | |  | | - Pathum Thani - Nonthaburi - Samut Prakan - Samut Sakhon | |
| 6 | |  | | For the next following questions, we’d like to ask you about your child aged between 5 and 11. If you have more than one child in this age group, please select the child who had the most recent birthday. If they are twins (or triplets or other multiple births!) please select the oldest twin. | | Select one | |  | | - I understand, continue. | |
| 7 | |  | | Have your child/children aged 5 to 11 received at least one dose of the COVID-19 vaccine? | | Select one | |  | | - Yes - No, but I've already booked a vaccination appointment - No | |
| 8 | | If ‘No, but I've already booked a vaccination appointment’ OR ‘No’ in Q7 | | Do you want to have your child/children vaccinated against COVID-19 if  vaccination is recommended and available for them? | | Select one | |  | | - Yes, definitely - Unsure, but leaning towards yes - Unsure, but leaning towards no - No, definitely not | |
| 9 | |  | | What is your child’s gender? | | Select one | |  | | - Male - Female - Other - Prefer not to say | |
| 10 | |  | | How old is your child? | | Select one | |  | | - 5 - 6 - 7 - 8 - 9 - 10 - 11 | |
| 11 | |  | | What is your child’s current education level? | | Select one | |  | | - Kindergarten - Elementary school | |
|  | |  | |  | |  | |  | | - Secondary school - High school | |
| 12 | |  | | Does your child have any of the underlying medical conditions which could increase their risk of severe illness from COVID- 19? | | Select many | | Please select all that apply. | | - Chronic disease (respiratory/kidney/h eart/liver) - Diabetes - Blood disease - Disease with an immune suppression - Receiving treatment that may suppress the immune system - Neurological or neuromuscular disease due to immune deficiency - Physical decline associated with a neurological disease or a neuromuscular disease - Chromosomal abnormality - Severe psychosomatic disorder - Depression/anxiety disorder - Sleep Apnea Syndrome - Obesity - None of the above - Other | |
| 13 | |  | | Does your child normally receive the influenza vaccine? | | Select one | |  | | - Yes - No - Unsure | |
| 14 | |  | | Has your child ever been diagnosed (tested positive or diagnosed by doctor) with COVID-19? | | Select one | |  | | - Yes - No - Unsure | |
| 15 | |  | | Has your child ever experienced a side effect or allergy after any vaccination? | | Select one | |  | | - Yes - No - Unsure | |
| 16 | |  | | "Overall, I think COVID-19 vaccines are IMPORTANT for children" | | Select one | | Please rate your level of agreement to the following statement. | | - Strongly agree - Tend to agree - Neither agree nor disagree | |
|  | |  | |  | |  | |  | | - Tend to disagree - Strongly disagree | |
| 17 | |  | | "Overall, I think COVID-19 vaccines  are SAFE for children." | | Select one | | Please rate your level of agreement to the following statement. | | - Strongly agree - Tend to agree - Neither agree nor disagree - Tend to disagree - Strongly disagree | |
| 18 | |  | | "Overall, I think COVID-19 vaccines are EFFECTIVE for children." | | Select one | | Please rate your level of agreement to the following statement. | | - Strongly agree - Tend to agree - Neither agree nor disagree - Tend to disagree - Strongly disagree | |
| 19 | |  | | "Vaccination of children is compatible with my religious/cultural beliefs." | | Select one | | Please rate your level of agreement to the following statement. | | - Strongly agree - Tend to agree - Neither agree nor disagree - Tend to disagree - Strongly disagree | |
| 20 | |  | | “I believe COVID-19 vaccines are effective for children regardless of the manufacturer of the vaccine” | | Select one | | Please rate your level of agreement to the following statement. | | - Strongly agree - Tend to agree - Neither agree nor disagree - Tend to disagree - Strongly disagree | |
| 21 | |  | | “I believe COVID-19 vaccines are effective for children against all variants of COVID-19” | | Select one | | Please rate your level of agreement to the following statement. | | - Strongly agree - Tend to agree - Neither agree nor disagree - Tend to disagree - Strongly disagree | |
| 22 | | If Unsure but leaning towards yes OR Unsure but leaning towards no OR No,  definitely not in Q8 | | From the following list, please select who’s recommendation might convince you to let your child receive a COVID-19 vaccine. | | Select one | |  | | - A friend - A parent - A teacher - A politician I like - A celebrity I like - The Government - A doctor or healthcare worker - Other - I don’t know | |
| 23 | | If Unsure, but leaning towards yes OR Unsure, but leaning towards no OR No,  definitely not in Q8 | | You indicated that you are either unsure or reluctant to the idea of your children receiving a COVID19 vaccine. Please briefly explain why. | | Text | |  | |  | |
| 24 | | If Unsure, but leaning towards yes OR Unsure, but leaning towards no OR No,  definitely not in Q8 | | Please tell us briefly what would change your mind about a COVID-19 vaccine and let your child be vaccinated? | | Text | |  | |  | |
| 25 | |  | | “My child may get COVID-19 infection within the next 6 months.” | | Select one | | Please rate your level of agreement to the following statement. | | - Strongly agree - Agree - Neither agree nor disagree - Disagree - Strongly disagree | |
| 26 | |  | | “COVID-19 is a serious disease” | | Select one | | Please rate your level of agreement to the following statement. | | - Strongly agree - Agree - Neither agree nor disagree - Disagree - Strongly disagree | |
| 27 | |  | | “Overall, I think COVID-19 vaccines are effective in reducing the risk of developing severe conditions.” | | Select one | | Please rate your level of agreement to the following statement. | | - Strongly agree - Agree - Neither agree nor disagree - Disagree - Strongly disagree | |
| 28 | |  | | “I believe I will be less anxious about my child’s chance of contracting the COVID- 19, if he/she is vaccinated.” | | Select one | | Please rate your level of agreement to the following statement. | | - Strongly agree - Agree - Neither agree nor disagree - Disagree - Strongly disagree | |
| 29 | |  | | “My child does not need to take a COVID- 19 vaccine because he/she practises social distancing and washes hands frequently with | | Select one | | Please rate your level of agreement to the following statement. | | - Strongly agree - Agree - Neither agree nor disagree - Disagree | |
|  | |  | | soap or sanitizer, which can help prevent the spread of COVID- 19.” | |  | |  | | - Strongly disagree | |
| 30 | |  | | “I believe vaccination of children can help control the spread of COVID-19.” | | Select one | | Please rate your level of agreement to the following statement. | | - Strongly agree - Agree - Neither agree nor disagree - Disagree - Strongly disagree | |
| 31 | |  | | “It is easy to find relevant information on COVID-19 vaccines on children.” | | Select one | | Please rate your level of agreement to the following statement. | | - Strongly agree - Agree - Neither agree nor disagree - Disagree - Strongly disagree | |
| 2 | |  | | “My child will take a COVID-19 vaccine if many others have  taken it.” | | Select one | | Please rate your level of agreement to the following statement. | | - Strongly agree - Agree - Neither agree nor disagree - Disagree - Strongly disagree | |
| 33 | |  | | “My child will take a COVID-19 vaccine if a COVID-19 vaccine certificate or passport is required for school, travel, social events or dine-in.” | | Select one | | Please rate your level of agreement to the following statement. | | - Strongly agree - Agree - Neither agree nor disagree - Disagree - Strongly disagree | |
| 34 | |  | | “COVID-19 vaccination should be compulsory for all children in Thailand.” | | Select one | | Please rate your level of agreement to the following statement. | | - Strongly agree - Agree - Neither agree nor disagree - Disagree - Strongly disagree | |
| 35 | |  | | “I think the government and health authorities are acting in the interest of public  health.” | | Select one | | Please rate your level of agreement to the following statement. | | - Strongly agree - Agree - Neither agree nor disagree - Disagree - Strongly disagree | |
| 36 | |  | | In the past month, on average, how much time per day have you | | Select one | |  | | - I’m not a social media user | |
|  | |  | | spent actively using social media? | |  | |  | | - Less than 10 minutes per day - 11–30 minutes per day - 31–60 minutes per day - 1--2 hours per day - 2–3 hours per day - More than 3 hours per day | |
| 37 | |  | | In the past month, from which of these social media platforms did you receive information regarding COVID-19 vaccines? | | Select many | | Please select all that apply. | | - Facebook - Twitter - YouTube - WhatsApp - LINE - Facebook Messenger - Instagram - TikTok - LinkedIn - Other | |
| 38 | |  | | From the following list, please select the source that you use and trust the MOST to learn about COVID-19 vaccines. | | Select one | |  | | - Local public television (e.g. NBT2HD, ThaiPBS) - Radio - International television (e.g. CNN, BBC) - Newspapers or magazines - Social media (e.g. Line, Facebook, Twitter, WhatsApp, etc.) - Local public health authorities, (e.g. the Ministry of Public Health, Department of Health Promotion) - Healthcare workers - Religious leaders - International health authorities (such as the World Health Organisation) - Community organisations - Scientists - Government websites - The internet or search engines (e.g. Google, Yahoo, etc.) - Family and friends | |

|  |  |  |  |  | - Work, school, or college/university - I don't know - Other |
| --- | --- | --- | --- | --- | --- |
| 39 |  | Have you ever seen or heard any COVID-19 associated information on social media which might have persuaded you NOT to immunise your children against COVID-19? | Select one |  | - Yes - No - I don’t know |
| 40 | If Yes in Q39 | Please describe what COVID-19 associated information on social media persuaded you NOT to immunise your children against COVID-19. | Text |  |  |
| 41 |  | Up next we will show you a set of statements and you will have to indicate if you think they are true, false or if you are unsure. | Select one |  | - I understand, continue. |
| 42 |  | “Many people have died after getting the COVID-19 vaccines” | Select one |  | - True - Unsure - False |
| 43 |  | “COVID-19 vaccination is associated with infertility and/or miscarriage” | Select one |  | - True - Unsure - False |
| 44 |  | “COVID-19 vaccines were approved without completing the normal process of the clinical trial” | Select one |  | - True - Unsure - False |
| 45 |  | Thank you for completing the first task of this study - the baseline survey. Next, please be reminded to complete the following to qualify for the post- intervention survey about chatbot user experience.  In the next week, please 1) use the |  |  | - I understand, and will start using the ‘Chat Sure' Messenger chatbot. |
|  |  | chatbot for more than ten minutes; 2) ensure that you have more than ten conversations with the chatbot.  Participants who meet the above requirements and complete the post- survey within a week will receive the rewards as a “thank you” for fulfilling the tasks. (After verification, those who were unable to complete the tasks within the designated time will not be eligible for compensation). Thank you very much for your cooperation. |  |  |  |

| Task title | Post-questionnaire – Child group (control/intervention) |
| --- | --- |
| Target locations | Thailand |
| Submission goal | 200 control / 200 intervention |

| # | Conditional logic | Question | Question type | Hint | Response options |
| --- | --- | --- | --- | --- | --- |
| 1 |  | We appreciate your participation in this conversational COVID19 chatbot evaluation. This is the third out of three tasks that you will need to complete. | Select one |  | - I understand, continue |
| 2 |  | Before we begin, could you please tell us which of the following statements best describe YOUR current COVID-19 vaccination status: | Select one |  | - 0 - I have not been vaccinated - 0 - I have not been vaccinated, but I plan to get vaccinated soon - I have received 1 dose - I have received 2 doses - I have received 3 doses |
| 3 |  | Now we would like to follow up on questions | Select one |  | - Yes |
|  |  | about your child for whom you completed the previous survey.  Has your child received at least one dose of the COVID-19 vaccine? |  |  | - No, but I've already booked a vaccination appointment - No |
| 4 | If Yes in Q3 | Please briefly tell us why you decided to get your child/children vaccinated. | Text |  |  |
| 5 | If No in Q3 | Do you want to have your child/children vaccinated against COVID-19 if it is recommended and available for them? | Select one |  | - Yes, definitely - Unsure, but leaning towards yes - Unsure, but leaning towards no - No, definitely not |
| 6 | If Yes definitely in Q5 | Please briefly tell us why you want your child/children to receive a COVID-19 vaccine. | Text |  |  |
| 7 | If any response BUT Yes definitely in Q5 | Please briefly tell us why you are hesitant or reluctant to the idea of your child/children receiving a COVID19 vaccine. | Text |  |  |
| 8 |  | Have you personally encouraged any of the following to take a COVID-19 vaccine? | Select one |  | - Spouse - Parent - Sibling - Son or daughter - Friend - Colleague - Other - I have not encouraged anyone to take a COVID-19 vaccine |
| 9 |  | Would you be willing for your child/children to receive an annual booster vaccine against COVID-19 if it is recommended and available for them? | Select one |  | - Yes, definitely - Unsure, but leaning towards yes - Unsure, but leaning towards no - No, definitely not |
| 10 |  | "Overall, I think COVID-19 vaccines are IMPORTANT for children" | Select one | Please rate your level of agreement to the following statement. | - Strongly agree - Tend to agree |
|  |  |  |  |  | - Neither agree nor disagree - Tend to disagree - Strongly disagree |
| 11 |  | "Overall, I think COVID-19 vaccines  are SAFE for children." | Select one | Please rate your level of agreement to the following statement. | - Strongly agree - Tend to agree - Neither agree nor disagree - Tend to disagree - Strongly disagree |
| 12 |  | "Overall, I think COVID-19 vaccines are EFFECTIVE for children." | Select one | Please rate your level of agreement to the following statement. | - Strongly agree - Tend to agree - Neither agree nor disagree - Tend to disagree - Strongly disagree |
| 13 |  | "Vaccination of children is compatible with my religious beliefs." | Select one | Please rate your level of agreement to the following statement. | - Strongly agree - Tend to agree - Neither agree nor disagree - Tend to disagree - Strongly disagree |
| 14 |  | “I believe COVID-19 vaccines are effective for children regardless of the manufacturer of the vaccine” | Select one | Please rate your level of agreement to the following statement. | - Strongly agree - Tend to agree - Neither agree nor disagree - Tend to disagree - Strongly disagree |
| 15 |  | “I believe COVID-19 vaccines are effective for children against all variants of COVID-19” | Select one | Please rate your level of agreement to the following statement. | - Strongly agree - Tend to agree - Neither agree nor disagree - Tend to disagree - Strongly disagree |
| 16 |  | “My child may get COVID-19 infection within the next 6 months.” | Select one | Please rate your level of agreement to the following statement. | - Strongly agree - Agree - Neither agree nor disagree - Disagree - Strongly disagree |
| 17 |  | “COVID-19 is a serious disease” | Select one | Please rate your level of agreement to the following statement. | - Strongly agree - Agree - Neither agree nor disagree - Disagree - Strongly disagree |
| 18 |  | “Overall, I think COVID-19 vaccines are effective in reducing the risk of developing severe conditions.” | Select one | Please rate your level of agreement to the following statement. | - Strongly agree - Agree - Neither agree nor disagree - Disagree - Strongly disagree |
| 19 |  | “I believe I will be less anxious about my child’s chance of contracting the COVID- 19, if he/she is vaccinated.” | Select one | Please rate your level of agreement to the following statement. | - Strongly agree - Agree - Neither agree nor disagree - Disagree - Strongly disagree |
| 20 |  | “My child does not need to take a COVID- 19 vaccine because he/she practises social distancing and washes hands frequently with soap or sanitizer, which can help prevent the spread of COVID- 19.” | Select one | Please rate your level of agreement to the following statement. | - Strongly agree - Agree - Neither agree nor disagree - Disagree - Strongly disagree |
| 21 |  | “I believe vaccination of children can help control the spread of COVID-19.” | Select one | Please rate your level of agreement to the following statement. | - Strongly agree - Agree - Neither agree nor disagree - Disagree - Strongly disagree |
| 22 |  | “It is easy to find relevant information on COVID-19 vaccines on children.” | Select one | Please rate your level of agreement to the following statement. | - Strongly agree - Agree - Neither agree nor disagree - Disagree - Strongly disagree |
| 23 |  | “My child will take a COVID-19 vaccine if many others have  taken it.” | Select one | Please rate your level of agreement to the following statement. | - Strongly agree - Agree - Neither agree nor disagree - Disagree |
|  |  |  |  |  | - Strongly disagree |
| 24 |  | “My child will take a COVID-19 vaccine if a COVID-19 vaccine certificate or passport is required for school, travel, social events or dine-in.” | Select one | Please rate your level of agreement to the following statement. | - Strongly agree - Agree - Neither agree nor disagree - Disagree - Strongly disagree |
| 25 |  | “COVID-19 vaccination should be compulsory for all children in Thailand.” | Select one | Please rate your level of agreement to the following statement. | - Strongly agree - Agree - Neither agree nor disagree - Disagree - Strongly disagree |
| 26 |  | “I think the government and health authorities are acting in the interest of public  health.” | Select one | Please rate your level of agreement to the following statement. | - Strongly agree - Agree - Neither agree nor disagree - Disagree - Strongly disagree |
| 27 |  | Have you ever seen or heard any COVID-19 associated information on social media which might have persuaded you NOT to immunise your children against COVID-19? | Select one |  | - Yes - No - I don’t know |
| 28 | If Yes in Q27 | Please describe what COVID-19 associated information on social media persuaded you NOT to immunise your children against COVID-19. | Text |  |  |
| 29 |  | Up next we will show you a set of statements and you will have to indicate if you think they are true, false or if you are unsure. | Select one |  | - I understand, continue. |
| 30 |  | “Many people have died after getting the COVID-19 vaccines” | Select one |  | - True - Unsure - False |
| 31 |  | “COVID-19 vaccination is associated with | Select one |  | - True |
|  |  | infertility and/or miscarriage” |  |  | - Unsure - False |
| 32 |  | “COVID-19 vaccines were approved without completing the normal process of the clinical trial” | Select one |  | - True - Unsure - False |
| 33 |  | Up next we will ask you to rate your level of agreement towards statements about your experience with the chatbot. | Select one |  | - I understand, continue. |
| 34 |  | In the past week, how much time in total have you spent on the chatbot? [Best estimate] | Select one |  | - I did not use the chatbot - Less than 10 minutes - 11-30 minutes - 31–60 minutes - 1-2 hours - 2-3 hours - More than 3 hours |
| 35 |  | “I found the information I was searching for” | Select one |  | - Strongly agree - Agree - Neither agree nor disagree - Disagree - Strongly disagree |
| 36 |  | What types of COVID19 related information were you seeking? | Select many | Please select all that apply. | - Vaccine safety - Vaccine effectiveness - Vaccine appointments - Vaccine centre locations - Vaccine boosters - Vaccines and pregnancy - Other |
| 37 | If Other in Q36 | What other types of information were you seeking? | Text |  |  |
| 38 |  | “The chatbot had a quick response time to my question(s)” | Select one |  | - Strongly agree - Agree - Neither agree nor disagree - Disagree |
|  |  |  |  |  | - Strongly disagree |
| 39 |  | “I intend to use the chatbot again” | Select one |  | - Strongly agree - Agree - Neither agree nor disagree - Disagree - Strongly disagree |
| 40 |  | “I like the design of the chatbot (font, colour, etc.)” | Select one |  | - Strongly agree - Agree - Neither agree nor disagree - Disagree - Strongly disagree |
| 41 |  | “I intend to recommend the chatbot to my  friends and family.” | Select one |  | - Strongly agree - Agree - Neither agree nor disagree - Disagree - Strongly disagree |
| 42 |  | Where do you think this chatbot should be offered? | Select many | Please select all that apply. | - Ministry of Public Health - Personal patient portal at Hospital Authority - Social media (e.g., Line, WhatsApp, Facebook, Twitter) - Other |
| 43 | If Other in Q42 | Where else do you think the chatbot should be offered? | Text |  |  |
| 44 |  | How could the chatbot be improved? | Select many | Please select all that apply. | - Quicker responses - More detailed information - Change in style of chatbot (e.g. font, colour, size, etc.) - ther |
| 45 | If Other in Q43 | What else could be done to improve the chatbot? | Text |  |  |

**Supplementary Method 6. English Questionnaire used in Hong Kong for both the control and intervention groups.**

| Task title | Pre-questionnaire – Senior group (control/intervention) |
| --- | --- |
| Target locations | Hong Kong |
| Submission goal | 200 control / 200 intervention |

| # | Conditional logic | | Question | | Question type | | Hint | | Response options | |
| --- | --- | --- | --- | --- | --- | --- | --- | --- | --- | --- |
| 1 |  | | We appreciate your participation in this conversational COVID19 chatbot evaluation. This is the first out of three tasks that you will need to complete. This survey will ask you some questions with regards to your elderly parents//grandparents (70 years old or above).  Before we begin the survey, please tell us about yourself: | | Select one | |  | | I understand, continue. | |
| 2 |  | | Please tell us which of the following statements best describe YOUR current COVID-19 vaccination status: | | Select one | |  | | - I have not been vaccinated, and I do not plan to get it anytime soon - I have not been vaccinated, but I plan to get vaccinated soon - I have received 1 dose - I have received 2 doses - I have received 3 doses | |
| 3 | If ‘I have not been vaccinated, and I do not plan to get it anytime soon’ OR I have not been vaccinated, but I plan to get vaccinated soon’ in Q2 | | Have you registered to get a COVID-19 vaccine? | | Select one | |  | | - Yes - No | |
| 4 |  | | Are you currently working in a healthcare setting? | | Select one | |  | | - Yes, as a medical doctor - Yes, as a health professional other than a medical doctor - No | |
| 5 |  | | What is your household monthly income bracket in HKD? | | Select one | |  | | - 0-5999 HKD - 6000-9999 HKD - 10000-14999 HKD - 15000-19999 HKD - 20000-24999 HKD - 25000-29999 HKD - 30000-39999 HKD - 40000-49999 HKD - 60000-79999 HKD - 80000-99999 HKD - ≥100,000 HKD - I don’t know - Prefer not to answer | |
| 6 |  | | Now we would like to ask some background questions about ONE elderly parent/grandparent. If both of your parents/grandparents are around and are 70 years old or above, please select the parent/grandparent who had the most recent birthday: | | Select one | |  | | - I understand, continue. | |
| 7 |  | | What is the vaccination status of your family member as of now? | | Select one | |  | | - My elderly family member is vaccinated with at least the first dose of the vaccine - My elderly family member has made an appointment but has not been vaccinated with the first dose yet - My elderly family member has not made an appointment but will get the vaccine. - My elderly family member has no plan to get vaccinated anytime soon. | |
| 8 | If you choose “My elderly family member has not made an appointment but will get the vaccine.” in Q7 | | When will your family member receive the first dose? | | Date | |  | |  | |
| 9 |  | | What is the relationship between you and the elderly parent/grandparent you are answering for in this survey? | | Select one | |  | | - Father - Mother - Grandfather - Grandmother | |
| 10 |  | | How old is your elderly family member? | | Select one | |  | | - Between 70 to 80 years old - Between 81 and 90 years old - Older than 91 years old | |
| 11 |  | | Does your elderly family member have any of the underlying medical conditions which could increase their risk of severe illness from COVID- 19? | | Select many | | Please select all that apply. | | - Chronic disease (respiratory/kidney/heart/liver) - Diabetes - Blood disease - Disease with an immune suppression | |
|  |  | |  | |  | |  | | - Receiving treatment that may suppress the immune system - Neurological or neuromuscular disease due to immune deficiency - Physical decline associated with a neurological disease or a neuromuscular disease - Chromosomal abnormality - Severe psychosomatic disorder - Depression/anxiety disorder - Sleep Apnea Syndrome - Obesity - None of the above - Other | |
| 12 |  | | Does your elderly family member normally receive the influenza vaccine? | | Select one | |  | | - Yes - No - Unsure | |
| 13 |  | | Has your elderly family member ever been diagnosed (tested positive or diagnosed by doctor) with COVID-19? | | Select one | |  | | - Yes - No - Unsure | |
| 14 |  | | Has your elderly family member ever experienced a side effect or allergy after any vaccination? | | Select one | |  | | - Yes - No - Unsure | |
| 15 |  | | "Overall, I think COVID-19 vaccines are IMPORTANT for the elderly." | | Select one | | Please rate your level of agreement to the following statement. | | - Strongly agree - Tend to agree - Neither agree nor disagree - Tend to disagree - Strongly disagree | |
| 16 |  | | "Overall, I think COVID-19 vaccines are SAFE for the elderly." | | Select one | | Please rate your level of agreement to the following statement. | | - Strongly agree - Tend to agree - Neither agree nor disagree - Tend to disagree - Strongly disagree | |
| 17 |  | | "Overall, I think COVID-19 vaccines are EFFECTIVE for the elderly." | | Select one | | Please rate your level of agreement to the following statement. | | - Strongly agree - Tend to agree - Neither agree nor disagree - Tend to disagree - Strongly disagree | |
| 18 |  | | "Vaccination of elderly people is compatible with my religious/cultural beliefs." | | Select one | | Please rate your level of agreement to the following statement. | | - Strongly agree - Tend to agree - Neither agree nor disagree - Tend to disagree - Strongly disagree | |
| 19 |  | | Do you want your elderly family member vaccinated against COVID-19 if vaccination is indicated and available for them? | | Select one | | Please rate your level of agreement to the following statement. | | - Yes, definitely - Unsure, but leaning towards yes - Unsure, but leaning towards no - No, definitely not | |
| 20 | If Unsure but leaning towards yes OR Unsure but leaning towards no OR No, definitely not in Q18 | | From the following list, please select whose recommendation might convince you to want your elderly family member vaccinated against COVID-19. | | Select many | |  | | - A friend - A parent - A teacher - A politician I like - A celebrity I like - The Government - A doctor or healthcare worker - Other - I don’t know | |
| 21 | If Unsure, but leaning towards yes OR Unsure, but leaning towards no OR No, definitely not in Q18 | | You indicated that you are either unsure or reluctant to the idea of your elder family member receiving a COVID19 vaccine. Please briefly explain why. | | Text | |  | |  | |
| 22 | If Unsure, but leaning towards yes OR Unsure, but leaning towards no OR No, definitely not in Q18 | | Please tell us briefly what could change your mind about how you feel with regards to your elder family member receiving a COVID19 vaccine in the future. | | Text | |  | |  | |
| 23 |  | | “My elderly family member may get COVID-19 infection within the next 6 month.” | | Select one | | Please rate your level of agreement to the following statement. | | - Strongly agree - Agree - Neither agree nor disagree - Disagree - Strongly disagree | |
| 24 |  | | “COVID-19 is a serious disease” | | Select one | | Please rate your level of agreement to the following statement. | | - Strongly agree - Agree - Neither agree nor disagree - Disagree - Strongly disagree | |
| 25 |  | | “Overall, I think COVID-19 vaccines are effective in reducing the risk of developing severe conditions.” | | Select one | | Please rate your level of agreement to the following statement. | | - Strongly agree - Tend to agree - Neither agree nor disagree - Tend to disagree - Strongly disagree | |
| 26 |  | | “I believe I will be less anxious about my elderly family member chance of contracting the COVID-19, if he/she is vaccinated” | | Select one | | Please rate your level of agreement to the following statement. | | - Strongly agree - Agree - Neither agree nor disagree - Disagree - Strongly disagree | |
| 27 |  | | “My elderly family member does not need to take a COVID-19 vaccine because they practise social distancing and wash hands frequently with soap or sanitizer, which can help prevent the spread of COVID-19” | | Select one | | Please rate your level of agreement to the following statement. | | - Strongly agree - Agree - Neither agree nor disagree - Disagree - Strongly disagree | |
| 28 |  | | “I believe vaccination of senior citizens can help control the spread of COVID-19” | | Select one | | Please rate your level of agreement to | | - Strongly agree - Agree - Neither agree nor disagree | |
|  | |  | |  | |  | | the following statement. | | - Disagree - Strongly disagree |
| 29 | |  | | “It is easy for my elderly family member to find relevant information on COVID-19 vaccines” | | Select one | | Please rate your level of agreement to the following statement. | | - Strongly agree - Agree - Neither agree nor disagree - Disagree - Strongly disagree |
| 30 | |  | | “I will want my elderly family member to take a COVID-19 vaccine if many others have taken it” | | Select one | | Please rate your level of agreement to the following statement. | | - Strongly agree - Agree - Neither agree nor disagree - Disagree - Strongly disagree |
| 31 | |  | | “I will want my elderly family member to take a COVID-19 vaccine if a COVID-19 vaccine certificate or passport is required for work, travel, social events or dine-in” | | Select one | | Please rate your level of agreement to the following statement. | | - Strongly agree - Agree - Neither agree nor disagree - Disagree - Strongly disagree |
| 32 | |  | | “COVID-19 vaccination should be compulsory for all senior citizens in Hong Kong” | | Select one | | Please rate your level of agreement to the following statement. | | - Strongly agree - Agree - Neither agree nor disagree - Disagree - Strongly disagree |
| 33 | |  | | In the past month, on average, how much time per day have you spent actively using social media? | | Select one | |  | | - I’m not a social media user - Less than 10 minutes per day - 11–30 minutes per day - 31–60 minutes per day - 1--2 hours per day - 2–3 hours per day - More than 3 hours per day |
| 34 | |  | | In the past month, from which of these social media platforms did you receive information regarding COVID- 19 vaccines? | | Select many | | Please select all that apply. | | - Facebook - Twitter - YouTube - WhatsApp - LINE - Facebook Messenger - Instagram - TikTok - LinkedIn - Other |
| 35 | |  | | From the following list, please select the source that you use and trust the MOST to learn about COVID-19 vaccines. | | Select one | |  | | - Local public television (e.g. TVB, ViuTV) - Radio - International television (e.g. CNN, BBC) - Newspapers or magazines - Social media (e.g. Facebook, Twitter, WhatsApp, etc.) - Local public health authorities, (e.g. Centre for Health Protection, Department of Health) - Healthcare workers - Religious leaders - International health authorities (such as the World Health Organisation) - Community organisations |
|  | |  | |  | |  | |  | | - Scientists - Government websites - The internet or search engines (e.g. Google, Yahoo, etc.) - Family and friends - Work, school, or college/university - I don’t know - Other |
| 36 | |  | | Have you ever seen or heard any COVID-19 associated information on social media which might have persuaded you NOT to immunise your elderly family member against COVID-19? | | Select one | |  | | - Yes - No - I don’t know |
| 37 | | If Yes in Q36 | | Please describe what COVID- 19 associated information on social media persuaded you NOT to immunise your elderly family member against COVID-19. | | Text | |  | |  |
| 38 | |  | | Up next we will show you a set of statements and you will have to indicate if you think they are true, false or if you are unsure. | | Select one | |  | | - I understand, continue. |
| 39 | |  | | “Genetic recombination technology is used in COVID- 19 vaccine to cause changes in genes (chromosomes)  through vaccination” | | Select one | |  | | - True - Unsure - False |
| 40 | |  | | “Many people have died after getting the COVID-19 vaccines” | | Select one | |  | | - True - Unsure - False |
| 41 | |  | | “COVID-19 vaccination is associated with infertility and/or miscarriage” | | Select one | |  | | - True - Unsure - False |
| 42 | |  | | “COVID-19 vaccination causes COVID-19 infection to those who receive the vaccines and people around them” | | Select one | |  | | - True - Unsure - False |
| 43 | |  | | “COVID-19 vaccines were approved without completing the normal process of the clinical trial” | | Select one | |  | | - True - Unsure - False |
| 44 | |  | | Thank you for completing the first task of this study - the baseline survey. Next, you will need to complete the following to qualify for the post- intervention survey about user experience. In the next week, please 1) use the chatbot for more than ten minutes; 2) ensure that you have more | | Select one | |  | | - I understand, and will start using the ‘D24H Covid Vaccine Chatbot’ |
|  | |  | | than ten conversations with the chatbot. Participants who meet the above requirements and complete the post-survey within a week will receive the rewards as a “thank you” for fulfilling the tasks. (After verification, those who were unable to complete the tasks within the designated time will not be eligible for compensation). Thank you very much for your cooperation. | |  | |  | |  |

| Task title | Post-questionnaire – Senior group (control/intervention) |
| --- | --- |
| Target locations | Hong Kong |
| Submission goal | 200 control / 200 intervention |

| # | Conditional logic | Question | Question type | Hint | Response options |
| --- | --- | --- | --- | --- | --- |
| 1 |  | We appreciate your participation in this conversational COVID19 chatbot evaluation. This is the third out of three tasks that you will need to complete. | Select one |  | - I understand, continue |
| 2 |  | Before we begin, could you please tell us which of the following statements best describe YOUR current COVID-19 vaccination status | Select one |  | - I have not been vaccinated, and I do not plan to get it anytime soon - I have not been vaccinated, but I plan to get vaccinated soon - I have received 1 dose - I have received 2 doses - I have received 3 doses |
| 3 |  | Now we would like to follow up on questions about your elderly family member for whom you completed the previous survey.  Has your elderly family member received at least one dose of the COVID-19 vaccine? | Select one |  | - Yes - No |
| 4 | If Yes in Q3 | Please briefly tell us why you decided to get your elderly family member vaccinated. | Text |  |  |
| 5 | If No in Q3 | Do you want your elderly family member to receive a COVID-19 vaccine in the future if vaccination is indicated and available for them? | Select one |  | - Yes, definitely - Unsure, but leaning towards yes - Unsure, but leaning towards no - No, definitely not |
| 6 | If Yes definitely in Q5 | Please briefly tell us why you want your elderly family member to receive a COVID-19 vaccine. | Text |  |  |
| 7 | If any response BUT Yes definitely in Q5 | Please briefly tell us why you are hesitant or reluctant to the idea of your elder family member receiving a COVID19 vaccine. | Text |  |  |
| 8 |  | Have you personally encouraged any of the following to take a COVID-19 vaccine? | Select many |  | - Spouse - Parent - Sibling - Son or daughter - Friend - Colleague - Other - I have not encouraged anyone to take a COVID-19 vaccine |
| 9 |  | Would you be willing for your elderly family member to receive an annual booster vaccine against COVID-19 if it is recommended and available for them? | Select one |  | - Yes, definitely - Unsure, but leaning towards yes - Unsure, but leaning towards no - No, definitely not |
| 10 |  | "Overall, I think COVID-19 vaccines are IMPORTANT for the elderly." | Select one | Please rate your level of agreement to the following statement. | - Strongly agree - Tend to agree - Neither agree nor disagree - Tend to disagree - Strongly disagree |
| 11 |  | "Overall, I think COVID-19 vaccines are SAFE for the elderly." | Select one | Please rate your level of agreement to the following statement. | - Strongly agree - Tend to agree - Neither agree nor disagree - Tend to disagree - Strongly disagree |
| 12 |  | "Overall, I think COVID-19 vaccines  are EFFECTIVE for the elderly." | Select one | Please rate your level of agreement to the following statement. | - Strongly agree - Tend to agree - Neither agree nor disagree - Tend to disagree - Strongly disagree |
| 13 |  | "Vaccination of elderly people is compatible with my religious/cultural beliefs." | Select one | Please rate your level of agreement to the following statement. | - Strongly agree - Tend to agree - Neither agree nor disagree - Tend to disagree - Strongly disagree |
| 14 |  | “My elderly family member may get COVID-19 infection within the next 6 month.” | Select one | Please rate your level of agreement to the following statement. | - Strongly agree - Agree - Neither agree nor disagree - Disagree - Strongly disagree |
| 15 |  | “COVID-19 is a serious disease” | Select one | Please rate your level of agreement to the following statement. | - Strongly agree - Agree - Neither agree nor disagree - Disagree - Strongly disagree |
| 16 |  | “Overall, I think COVID-19 vaccines are effective in reducing the risk of developing severe conditions.” | Select one | Please rate your level of agreement to the following statement. | - Strongly agree - Agree - Neither agree nor disagree - Disagree - Strongly disagree |
| 17 |  | “I believe I will be less anxious about my elderly family member chance of contracting the COVID-19, if  he/she is vaccinated” | Select one | Please rate your level of agreement to the following statement. | - Strongly agree - Agree - Neither agree nor disagree - Disagree - Strongly disagree |
| 18 |  | “My elderly family member does not need to take a COVID-19 vaccine because they practise social distancing and wash hands frequently with soap or sanitizer, which can help prevent the spread of COVID-19” | Select one | Please rate your level of agreement to the following statement. | - Strongly agree - Agree - Neither agree nor disagree - Disagree - Strongly disagree |
| 19 |  | “I believe vaccination of senior citizens can help control the spread of COVID-19” | Select one | Please rate your level of agreement to the following statement. | - Strongly agree - Agree - Neither agree nor disagree - Disagree - Strongly disagree |
| 20 |  | “It is easy for my elderly family member to find relevant information on COVID- 19 vaccines” | Select one | Please rate your level of agreement to the following statement. | - Strongly agree - Agree - Neither agree nor disagree - Disagree - Strongly disagree |
| 21 |  | “I will want my elderly family member to take a COVID-19 vaccine if many others have  taken it” | Select one | Please rate your level of agreement to the following statement. | - Strongly agree - Agree - Neither agree nor disagree - Disagree - Strongly disagree |
| 22 |  | “I will want my elderly family member to take a COVID-19 vaccine if a COVID-19 vaccine certificate or passport is required for work, travel, social events or dine-in” | Select one | Please rate your level of agreement to the following statement. | - Strongly agree - Agree - Neither agree nor disagree - Disagree - Strongly disagree |
| 23 |  | “COVID-19 vaccination should be compulsory for all senior citizens in Hong Kong” | Select one | Please rate your level of agreement to the following statement. | - Strongly agree - Agree - Neither agree nor disagree - Disagree - Strongly disagree |
| 24 |  | Have you ever seen or heard any COVID-19 associated information on social media which might have persuaded you NOT to immunise your elderly family member against COVID-19? | Select one |  | - Yes - No - I don’t know |
| 25 | If Yes in Q24 | Please describe what COVID-19 associated information on social media persuaded you NOT to immunise your elderly family member against COVID-19. | Text |  |  |
| 26 |  | Up next we will show you a set of statements and you will have to indicate if you think they are true, false or if you are unsure. | Select one |  | - I understand, continue. |
| 27 |  | “Genetic recombination technology is used in COVID-19 vaccine to cause changes in genes (chromosomes) through vaccination” | Select one |  | - True - Unsure - False |
| 28 |  | “Many people have died after getting the COVID-19 vaccines” | Select one |  | - True - Unsure - False |
| 29 |  | “COVID-19 vaccination is associated with infertility and/or miscarriage” | Select one |  | - True - Unsure - False |
| 30 |  | “COVID-19 vaccination causes COVID-19 infection to those who receive the vaccines and people around them” | Select one |  | - True - Unsure - False |
| 31 |  | “COVID-19 vaccines were approved without completing the normal process of the clinical trial” | Select one |  | - True - Unsure - False |
| 32 |  | Up next we will ask you to rate your level of agreement towards statements about your | Select one |  | - I understand, continue. |
|  |  | experience with the chatbot. |  |  |  |
| 33 |  | In the past week, how much time in total have you spent on the chatbot? [Best estimate] | Select one |  | - I did not use the chatbot - Less than 10 minutes - 11-30 minutes - 31–60 minutes - 1-2 hours - 2-3 hours - More than 3 hours |
| 34 |  | “I found the information I was searching for” | Select one |  | - Strongly agree - Agree - Neither agree nor disagree - Disagree - Strongly disagree |
| 35 |  | What types of COVID19 related information were you seeking? | Select many | Please select all that apply. | - Vaccine safety - Vaccine effectiveness - Vaccine appointments - Vaccine centre locations - Vaccine boosters - Vaccines and pregnancy - Other |
| 36 | If Other in Q35 | What other types of information were you seeking? | Text |  |  |
| 37 |  | “The chatbot had a quick response time to my question(s)” | Select one |  | - Strongly agree - Agree - Neither agree nor disagree - Disagree - Strongly disagree |
| 38 |  | “I intend to use the chatbot again” | Select one |  | - Strongly agree - Agree - Neither agree nor disagree - Disagree - Strongly disagree |
| 39 |  | “I like the design of the chatbot (font, colour, etc.)” | Select one |  | - Strongly agree - Agree - Neither agree nor disagree - Disagree - Strongly disagree |
| 40 |  | “I intend to recommend the chatbot to my  friends and family.” | Select one |  | - Strongly agree - Agree - Neither agree nor disagree - Disagree - Strongly disagree |
| 41 |  | On which platform would this chatbot be most helpful? | Select many | Please select all that apply. | - Department of Health website - Personal patient portal at Hospital Authority - Social media (e.g., WhatsApp, Facebook, Twitter) - Other |
| 42 | If Other in Q41 | On which platform would this chatbot be most helpful? | Text |  |  |
| 43 |  | How could the chatbot be improved? | Select many | Please select all that apply. | - Quicker responses - More detailed information - Change in style of chatbot (e.g. font, colour, size, etc.) - Other |
| 44 | If Other in Q43 | What else could be done to improve the chatbot? | Text |  |  |

| Task title | Pre-questionnaire – Child group (control/intervention) |
| --- | --- |
| Target locations | Hong Kong |
| Submission goal | 200 control / 200 intervention |

| # | | Conditional logic | | Question | | | Question type | | | Hint | | | Response options | | |  |
| --- | --- | --- | --- | --- | --- | --- | --- | --- | --- | --- | --- | --- | --- | --- | --- | --- |
| 1 | |  | | We appreciate your participation in this conversational COVID19 chatbot evaluation. This is the first out of three tasks that you will need to complete. This survey will ask you some questions with regards to the younger members of your family.  Before we begin the survey, please tell us about yourself: | | | Select one | | |  | | | - I understand, continue. | | |  |
| 2 | |  | | Are you currently working in a healthcare setting? | | | Select one | | |  | | | - Yes, as a medical doctor - Yes, as a health professional other than a medical doctor - No | | |  |
| 3 | |  | | What is your household monthly income bracket? | | | Select one | | |  | | | - 0-5999 HKD - 6000-9999 HKD - 10000-14999 HKD - 15000-19999 HKD - 20000-24999 HKD - 25000-29999 HKD - 30000-39999 HKD - 40000-49999 HKD - 60000-79999 HKD - 80000-99999 HKD - ≥100,000 HKD - I don’t know - Prefer not to answer   ● | | |  |
| 4 | |  | | Please tell us which of the following statements best | | | Select one | | |  | | | - I have not been vaccinated, and I do not plan to get it anytime soon | | |  |
|  |  | | | | describe YOUR current COVID-19 vaccination status | | |  | | |  | | | - I have not been vaccinated, but I plan to get vaccinated soon - I have received 1 dose - I have received 2 doses - I have received 3 doses | |  |
| 5 | If ‘I have not been vaccinated, and I do not plan to get it anytime soon’ OR I have not been vaccinated, but I plan to get vaccinated soon’ in Q4 | | | | Have you registered to get a COVID-19 vaccine? | | | Select one | | |  | | | - Yes - No | |  |
| 6 |  | | | | For the next following questions, we’d like to ask you about your child aged under 18. If you have more than one child in this age group, please select the child who had the most recent birthday. If they are twins (or triplets or other multiple births!) please select the oldest twin. | | | Select one | | |  | | | - I understand, continue. | |  |
| 7 |  | | | | Have your child/children aged 5 to 11 received at least one dose of the COVID-19 vaccine? | | | Select one | | |  | | | - Yes - No, but I've already booked a vaccination appointment - No | |  |
| 8 | If ‘No, but I've already booked a vaccination appointment’ OR ‘No’ in Q7 | | | | Do you want to have your child/children vaccinated against COVID-19 if  vaccination is recommended and available for them? | | | Select one | | |  | | | - Yes, definitely - Unsure, but leaning towards yes - Unsure, but leaning towards no - No, definitely not | |  |
| 9 |  | | | | What is your child’s gender? | | | Select one | | |  | | | - Male - Female - Non-binary - Prefer not to say | |  |
| 10 |  | | | | How old is your child? | | | Select one | | |  | | | - 1 - 2 - 3 - 4 - 5 - 6 - 7 - 8 - 9 | |  |
|  | |  | |  | | |  | | |  | | | - 10 - 11 - 12 - 13 - 14 - 15 - 16 - 17 | | |  |
| 11 | |  | | What is your child’s current education level? | | | Select one | | |  | | | - Kindergarten - Elementary school - Secondary school - High school | | |  |
| 12 | |  | | Does your child have any of the underlying medical conditions which could increase their risk of severe illness from COVID- 19? | | | Select many | | | Please select all that apply. | | | - Chronic disease (respiratory/kidney/heart/liver) - Diabetes - Blood disease - Disease with an immune suppression - Receiving treatment that may suppress the immune system - Neurological or neuromuscular disease due to immune deficiency - Physical decline associated with a neurological disease or a neuromuscular disease - Chromosomal abnormality - Severe psychosomatic disorder - Depression/anxiety disorder - Sleep Apnea Syndrome - Obesity - None of the above - Other | | |  |
| 13 | |  | | Does your child normally receive the influenza vaccine? | | | Select one | | |  | | | - Yes - No - Unsure | | |  |
| 14 | |  | | Has your child ever been diagnosed (tested positive or diagnosed by doctor) with COVID- 19? | | | Select one | | |  | | | - Yes - No - Unsure | | |  |
| 15 | |  | | Has your child ever experienced a side effect or allergy after any vaccination? | | | Select one | | |  | | | - Yes - No - Unsure | | |  |
| 16 | |  | | "Overall, I think COVID-19 vaccines are IMPORTANT for children" | | | Select one | | | Please rate your level of agreement to the following statement. | | | - Strongly agree - Tend to agree - Neither agree nor disagree - Tend to disagree - Strongly disagree | | |  |
| 17 | |  | | "Overall, I think COVID-19 vaccines  are SAFE for children." | | | Select one | | | Please rate your level of agreement to the following statement. | | | - Strongly agree - Tend to agree - Neither agree nor disagree - Tend to disagree - Strongly disagree | | |  |
| 18 | |  | | "Overall, I think COVID-19 vaccines are EFFECTIVE for children." | | | Select one | | | Please rate your level of agreement to the following statement. | | | - Strongly agree - Tend to agree - Neither agree nor disagree - Tend to disagree - Strongly disagree | | |  |
| 19 | |  | | "Vaccination of children is compatible with my religious/cultural beliefs." | | | Select one | | | Please rate your level of agreement to the following statement. | | | - Strongly agree - Tend to agree - Neither agree nor disagree - Tend to disagree - Strongly disagree | | |  |
| 20 | | If Unsure but leaning towards yes OR Unsure but leaning towards no OR No,  definitely not in Q8 | | From the following list, please select who’s recommendation might convince you to let your child receive a COVID-19 vaccine. | | | Select one | | |  | | | - A friend - A parent - A teacher - A politician I like - A celebrity I like - The Government - A doctor or healthcare worker - Other - I don’t know | | |  |
| 21 | | If Unsure, but leaning towards yes OR Unsure, but leaning towards no OR No,  definitely not in Q8 | | You indicated that you are either unsure or reluctant to the idea of your children receiving a COVID19 vaccine. Please briefly explain why. | | | Text | | |  | | |  | | |  |
| 22 | | If Unsure, but leaning towards yes OR Unsure, but leaning towards no OR No,  definitely not in Q8 | | Please tell us briefly what would change your mind about a COVID-19 vaccine and let your child be vaccinated? | | | Text | | |  | | |  | | |  |
| 23 | |  | | “My child may get COVID-19 infection within the next 6 months.” | | | Select one | | | Please rate your level of agreement to the following statement. | | | - Strongly agree - Agree - Neither agree nor disagree - Disagree - Strongly disagree | | |  |
| 24 | |  | | “COVID-19 is a serious disease” | | | Select one | | | Please rate your level of agreement to the following statement. | | | - Strongly agree - Agree - Neither agree nor disagree - Disagree - Strongly disagree | | |  |
| 25 | |  | | “Overall, I think COVID-19 vaccines are effective in reducing the risk of developing severe conditions.” | | | Select one | | | Please rate your level of agreement to the following statement. | | | - Strongly agree - Agree - Neither agree nor disagree - Disagree - Strongly disagree | | |  |
| 26 | |  | | “I believe I will be less anxious about my child’s chance of contracting the COVID- 19, if he/she is vaccinated.” | | | Select one | | | Please rate your level of agreement to the following statement. | | | - Strongly agree - Agree - Neither agree nor disagree - Disagree - Strongly disagree | | |  |
| 27 | |  | | “My child does not need to take a COVID- 19 vaccine because he/she practises social distancing and washes hands frequently with soap or sanitizer, which can help prevent the spread of COVID-19.” | | | Select one | | | Please rate your level of agreement to the following statement. | | | - Strongly agree - Agree - Neither agree nor disagree - Disagree - Strongly disagree | | |  |
| 28 | |  | | “I believe vaccination of children can help control the spread of COVID-19.” | | | Select one | | | Please rate your level of agreement to the following statement. | | | - Strongly agree - Agree - Neither agree nor disagree - Disagree - Strongly disagree | | |  |
| 29 | |  | | “It is easy to find relevant information on COVID-19 vaccines on children.” | | | Select one | | | Please rate your level of agreement to the following statement. | | | - Strongly agree - Agree - Neither agree nor disagree - Disagree - Strongly disagree | | |  |
| 30 | |  | | “My child will take a COVID-19 vaccine if many others have  taken it.” | | | Select one | | | Please rate your level of agreement to the following statement. | | | - Strongly agree - Agree - Neither agree nor disagree - Disagree - Strongly disagree | | |  |
| 31 | |  | | “My child will take a COVID-19 vaccine if a COVID-19 vaccine certificate or passport is required for school, travel, social events or dine-in..” | | | Select one | | | Please rate your level of agreement to the following statement. | | | - Strongly agree - Agree - Neither agree nor disagree - Disagree - Strongly disagree | | |  |
| 32 | |  | | “COVID-19 vaccination should be compulsory for all children in Hong Kong.” | | | Select one | | | Please rate your level of agreement to the following statement. | | | - Strongly agree - Agree - Neither agree nor disagree - Disagree - Strongly disagree | | |  |
| 33 | |  | | In the past month, on average, how much time per day have you spent actively using social media? | | | Select one | | |  | | | - I’m not a social media user - Less than 10 minutes per day - 11–30 minutes per day - 31–60 minutes per day - 1--2 hours per day - 2–3 hours per day - More than 3 hours per day | | |  |
| 34 | |  | | In the past month, from which of these social media platforms did you receive information regarding COVID-19 vaccines? | | | Select many | | | Please select all that apply. | | | - Facebook - Twitter - YouTube - WhatsApp - LINE - Facebook Messenger - Instagram - TikTok - LinkedIn - Other | | |  |
| 35 | |  | | From the following list, please select the source that you use and trust the MOST to learn about COVID-19 vaccines. | | | Select one | | |  | | | - Local public television (e.g. TVB, ViuTV) - Radio - International television (e.g. CNN, BBC) - Newspapers or magazines - Social media (e.g. Facebook, Twitter, WhatsApp, etc.) - Local public health authorities, (e.g. Centre for Health Protection, Department of Health) - Healthcare workers - Religious leaders - International health authorities (such as the World Health Organisation) - Community organisations - Scientists - Government websites - The internet or search engines (e.g. Google, Yahoo, etc.) - Family and friends - Work, school, or college/university - I don’t know - Other | | |  |
| 36 | |  | | Have you ever seen or heard any COVID-19 associated information on social media which might have persuaded you NOT to immunise your children against COVID-19? | | | Select one | | |  | | | - Yes - No - I don’t know | | |  |
| 37 | | If Yes in Q36 | | Please describe what COVID-19 associated information on social media persuaded you NOT to immunise your children against COVID-19. | | | Text | | |  | | |  | | |  |
| 38 | |  | | Up next we will show you a set of statements and you will have to indicate if you think they are true, false or if you are unsure. | | | Select one | | |  | | | - I understand, continue. | | |  |
| 39 | |  | | “Genetic recombination technology is used in COVID-19 vaccine to | | | Select one | | |  | | | - True - Unsure - False | | |  |
|  | | |  | | | cause changes in genes (chromosomes) through vaccination” | | |  | | |  | | |  | |
| 40 | | |  | | | “Many people have died after getting the COVID-19 vaccines” | | | Select one | | |  | | | - True - Unsure - False | |
| 41 | | |  | | | “COVID-19 vaccination is associated with infertility and/or miscarriage” | | | Select one | | |  | | | - True - Unsure - False | |
| 42 | | |  | | | “COVID-19 vaccination causes COVID-19 infection to those who receive the vaccines and people around them” | | | Select one | | |  | | | - True - Unsure - False | |
| 43 | | |  | | | “COVID-19 vaccines were approved without completing the normal process of the clinical trial” | | | Select one | | |  | | | - True - Unsure - False | |
| 44 | | |  | | | Thank you for completing the first task of this project. The upcoming task ‘COVID19  Perception 2’ will be available in the next couple of days. If you complete all tasks, you can earn up to XXX!  Remember to check your Premise Marketplace often! | | |  | | |  | | | - I understand, continue. | |
|  |  |  |  |  |  |  | | |  |  |  |  |  |  |  |  |

| Task title | Post-questionnaire – Child group (control/intervention) |
| --- | --- |
| Target locations | Hong Kong |
| Submission goal | 200 control / 200 intervention |
| Recurrence period | None |

| # | Conditional logic | | Question | | Question type | Hint | | Response options | | |
| --- | --- | --- | --- | --- | --- | --- | --- | --- | --- | --- |
| 1 |  | | We appreciate your participation in this conversational COVID19 chatbot evaluation. This is the | | Select one |  | | - I understand, continue | | |
|  | |  | | third out of three tasks that you will need to complete. | | |  | |  |  |
| 2 | |  | | Before we begin, could you please tell us which of the following statements best describe YOUR current COVID-19 vaccination status: | | | Select one | |  | - 0 - I have not been vaccinated - 0 - I have not been vaccinated, but I plan to get vaccinated soon - I have received 1 dose - I have received 2 doses - I have received 3 doses |
| 3 | |  | | Now we would like to follow up on questions about your child for whom you completed the previous survey.  Has your child received at least one dose of the COVID-19 vaccine? | | | Select one | |  | - Yes - No, but I've already booked a vaccination appointment - No |
| 4 | | If Yes in Q3 | | Please briefly tell us why you decided to get your child/children vaccinated. | | | Text | |  |  |
| 5 | | If No in Q3 | | Do you want to have your child/children vaccinated against COVID-19 if it is recommended and available for them? | | | Select one | |  | - Yes, definitely - Unsure, but leaning towards yes - Unsure, but leaning towards no - No, definitely not |
| 6 | | If Yes definitely in Q5 | | Please briefly tell us why you want your child/children to receive a COVID-19 vaccine. | | | Text | |  |  |
| 7 | | If any response BUT Yes definitely in Q5 | | Please briefly tell us why you are hesitant or reluctant to the idea of your child/children receiving a COVID19 vaccine. | | | Text | |  |  |
| 8 | |  | | Have you personally encouraged any of the following to take a COVID-19 vaccine? | | | Select one | |  | - Spouse - Parent - Sibling - Son or daughter - Friend - Colleague - Other - I have not encouraged anyone to take a COVID-19 vaccine |
| 9 | |  | | Would you be willing for your child/children to receive an annual booster vaccine against COVID-19 if it | | | Select one | |  | - Yes, definitely - Unsure, but leaning towards yes - Unsure, but leaning towards no - No, definitely not |
|  | |  | | is recommended and available for them? | | |  | |  |  |
| 10 | |  | | "Overall, I think COVID-19 vaccines are IMPORTANT for children" | | | Select one | | Please rate your level of agreement to the following statement. | - Strongly agree - Tend to agree - Neither agree nor disagree - Tend to disagree - Strongly disagree |
| 11 | |  | | "Overall, I think COVID-19 vaccines  are SAFE for children." | | | Select one | | Please rate your level of agreement to the following statement. | - Strongly agree - Tend to agree - Neither agree nor disagree - Tend to disagree - Strongly disagree |
| 12 | |  | | "Overall, I think COVID-19 vaccines are EFFECTIVE for children." | | | Select one | | Please rate your level of agreement to the following statement. | - Strongly agree - Tend to agree - Neither agree nor disagree - Tend to disagree - Strongly disagree |
| 13 | |  | | "Vaccination of children is compatible with my religious beliefs." | | | Select one | | Please rate your level of agreement to the following statement. | - Strongly agree - Tend to agree - Neither agree nor disagree - Tend to disagree - Strongly disagree |
| 14 | |  | | “My child may get COVID-19 infection within the next 6 months.” | | | Select one | | Please rate your level of agreement to the following statement. | - Strongly agree - Agree - Neither agree nor disagree - Disagree - Strongly disagree |
| 15 | |  | | “COVID-19 is a serious disease” | | | Select one | | Please rate your level of agreement to the following statement. | - Strongly agree - Agree - Neither agree nor disagree - Disagree - Strongly disagree |
| 16 | |  | | “Overall, I think COVID-19 vaccines are effective in reducing the risk of developing severe conditions.” | | | Select one | | Please rate your level of agreement to the following statement. | - Strongly agree - Agree - Neither agree nor disagree - Disagree - Strongly disagree |
| 17 | |  | | “I believe I will be less anxious about my child’s chance of contracting the COVID- 19, if he/she is vaccinated.” | | | Select one | | Please rate your level of agreement to the following statement. | - Strongly agree - Agree - Neither agree nor disagree - Disagree - Strongly disagree |
| 18 | |  | | “My child does not need to take a COVID- 19 vaccine because he/she practises social distancing and washes hands frequently with soap or sanitizer, which can help prevent the spread of COVID-19.” | | | Select one | | Please rate your level of agreement to the following statement. | - Strongly agree - Agree - Neither agree nor disagree - Disagree - Strongly disagree |
| 19 | |  | | “I believe vaccination of children can help control the spread of COVID-19.” | | | Select one | | Please rate your level of agreement to the following statement. | - Strongly agree - Agree - Neither agree nor disagree - Disagree - Strongly disagree |
| 20 | |  | | “It is easy to find relevant information on COVID-19 vaccines on children.” | | | Select one | | Please rate your level of agreement to the following statement. | - Strongly agree - Agree - Neither agree nor disagree - Disagree - Strongly disagree |
| 21 | |  | | “My child will take a COVID-19 vaccine if many others have  taken it.” | | | Select one | | Please rate your level of agreement to the following statement. | - Strongly agree - Agree - Neither agree nor disagree - Disagree - Strongly disagree |
| 22 | |  | | “My child will take a COVID-19 vaccine if a COVID-19 vaccine certificate or passport is required for school, travel, social events or dine-in.” | | | Select one | | Please rate your level of agreement to the following statement. | - Strongly agree - Agree - Neither agree nor disagree - Disagree - Strongly disagree |
| 23 | |  | | “COVID-19 vaccination should be compulsory for all children in Hong Kong.” | | | Select one | | Please rate your level of agreement to the following statement. | - Strongly agree - Agree - Neither agree nor disagree - Disagree - Strongly disagree |
| 24 | |  | | Have you ever seen or heard any COVID-19 associated information on social media which might have persuaded you NOT to immunise your children against COVID-19? | | | Select one | |  | - Yes - No - I don’t know |
| 25 | | If Yes in Q24 | | Please describe what COVID-19 associated information on social media persuaded you NOT to immunise your children against COVID-19. | | | Text | |  |  |
| 26 | |  | | Up next we will show you a set of statements and you will have to indicate if you think they are true, false or if you are unsure. | | | Select one | |  | - I understand, continue. |
| 27 | |  | | “Genetic recombination technology is used in COVID-19 vaccine to cause changes in genes (chromosomes) through vaccination” | | | Select one | |  | - True - Unsure - False |
| 28 | |  | | “Many people have died after getting the COVID-19 vaccines” | | | Select one | |  | - True - Unsure - False |
| 29 | |  | | “COVID-19 vaccination is associated with infertility and/or miscarriage” | | | Select one | |  | - True - Unsure - False |
| 30 | |  | | “COVID-19 vaccination causes COVID-19 infection to those who receive the vaccines and people around them” | | | Select one | |  | - True - Unsure - False |
| 31 | |  | | “COVID-19 vaccines were approved without completing the normal process of the clinical trial” | | | Select one | |  | - True - Unsure - False |
| 32 | |  | | Up next we will ask you to rate your level of agreement towards statements about your experience with the chatbot. | | | Select one | |  | - I understand, continue. |
| 33 | |  | | In the past week, how much time in total have you spent on the chatbot? [Best estimate] | | | Select one | |  | - I did not use the chatbot - Less than 10 minutes - 11-30 minutes - 31–60 minutes - 1-2 hours - 2-3 hours - More than 3 hours |
| 34 | |  | | “I found the information I was searching for” | | | Select one | |  | - Strongly agree - Agree - Neither agree nor disagree - Disagree - Strongly disagree |
| 35 | |  | | What types of COVID19 related information were you seeking? | | | Select many | | Please select all that apply. | - Vaccine safety - Vaccine effectiveness - Vaccine appointments - Vaccine centre locations - Vaccine boosters - Vaccines and pregnancy - Other |
| 36 | | If Other in Q35 | | What other types of information were you seeking? | | | Text | |  |  |
| 37 | |  | | “The chatbot had a quick response time to my question(s)” | | | Select one | |  | - Strongly agree - Agree - Neither agree nor disagree - Disagree - Strongly disagree |
| 38 | |  | | “I intend to use the chatbot again” | | | Select one | |  | - Strongly agree - Agree - Neither agree nor disagree |
|  | |  | |  | | |  | |  | - Disagree - Strongly disagree |
| 39 | |  | | “I like the design of the chatbot (font, colour, etc.)” | | | Select one | |  | - Strongly agree - Agree - Neither agree nor disagree - Disagree - Strongly disagree |
| 40 | |  | | “I intend to recommend the chatbot to my  friends and family.” | | | Select one | |  | - Strongly agree - Agree - Neither agree nor disagree - Disagree - Strongly disagree |
| 41 | |  | | On which platform would this chatbot be most helpful? | | | Select many | | Please select all that apply. | - Department of Health website - Personal patient portal at Hospital Authority - Social media (e.g., WhatsApp, Facebook, Twitter) - Other |
| 42 | | If Other in Q41 | | On which platform would this chatbot be most helpful? | | | Text | |  |  |
| 43 | |  | | How could the chatbot be improved? | | | Select many | | Please select all that apply. | - Quicker responses - More detailed information - Change in style of chatbot (e.g. font, colour, size, etc.) - Other |
| 44 | | If Other in Q43 | | What else could be done to improve the chatbot? | | | Text | |  |  |

**Supplementary Method 7. English Questionnaire used in Singapore for both the control and intervention groups.**

| Task title | Pre-questionnaire – Child group (control/intervention) |
| --- | --- |
| Target locations | Singapore |
| Submission goal | 200 control / 200 intervention |

| # | Conditional logic | | Question | | | Question type | | | | Hint | | | Response options | |
| --- | --- | --- | --- | --- | --- | --- | --- | --- | --- | --- | --- | --- | --- | --- |
| 1 |  | | We appreciate your participation in this conversational COVID19 Chatbot evaluation. This is the first out of three tasks that you will need to complete. This survey will ask you some questions with regards to the younger members of your family.  Before we begin the survey, please tell us about yourself: | | | Select one | | | |  | | | I understand, continue. | |
| 2 |  | | Are you currently working in healthcare settings? | | | Select one | | | |  | | | - Yes, I am a physician - Yes, but I am not a physician - No | |
| 3 |  | | From the following list, please select the type of housing that you live in. | | | Select one | | | |  | | | - 1-room HDB - 2-room HDB - 3-room HDB - 4-room HDB - 5-room HDB - Executive Condo - Condominium - Shophouse - Landed - Prefer not to answer | |
| 4 |  | | Please tell us which of the following statements best describe YOUR current COVID-19 vaccination status | | | Select one | | | |  | | | - 0 - I have not been vaccinated and I do not plan to get it anytime soon - 0 - I have not been vaccinated, but I plan to get vaccinated soon - 1 dose - 2 doses - 3 doses | |
| 5 | If ‘I have not been vaccinated, and I do not plan to get it anytime soon’ OR I have not been vaccinated, but I plan to get vaccinated soon’ in Q5 | | Have you registered to get a COVID-19 vaccine? | | | Select one | | | |  | | | - Yes - No | |
| 6 |  | | Do you normally receive the influenza vaccine? | | | Select one | | | |  | | | - Yes - No - Unsure | |
| 7 |  | | Have you ever experienced side effects or allergic reactions (e.g. anaphylaxis) after vaccination? | | | Select one | | | |  | | | - Yes, but minor symptoms (e.g. muscle aches, headache, fever) - Yes, serious adverse effects leading to ER visits or hospitalizations - No - I don’t know | |
| 8 | |  | | | Have you ever tested positive for COVID- 19? | | | Select one | | |  | | | - Yes - No - Unsure |
| 9 | |  | | | “Overall, I think COVID-19 vaccines are IMPORTANT FOR YOUNG CHILDREN.” | | | Select one | | | Please rate your level of agreement to the following statement. | | | - Strongly agree - Tend to agree - Neither agree nor disagree - Tend to disagree - Strongly disagree |
| 10 | |  | | | "Overall, I think COVID-19 vaccines are SAFE FOR YOUNG CHILDREN." | | | Select one | | | Please rate your level of agreement to the following statement. | | | - Strongly agree - Tend to agree - Neither agree nor disagree - Tend to disagree - Strongly disagree |
| 11 | |  | | | “Overall, I think COVID-19 vaccines are effective at preventing infection.” | | | Select one | | | Please rate your level of agreement to the following statement. | | | - Strongly agree - Tend to agree - Neither agree nor disagree - Tend to disagree - Strongly disagree |
| 12 | |  | | | “Overall, I think COVID-19 vaccines are effective in reducing the risk of developing severe conditions.” | | | Select one | | | Please rate your level of agreement to the following statement. | | | - Strongly agree - Tend to agree - Neither agree nor disagree - Tend to disagree - Strongly disagree |
| 13 | |  | | | For the next following questions, we’d like to ask you about your child aged between 5 and 11. If you have more than one child in this age group, please select the child who had the most recent birthday. If they are twins (or triplets or other multiple births!) please select the oldest twin. | | | Select one | | |  | | | - I understand, continue. |
| 14 | |  | | | What is your child’s gender? | | | Select one | | |  | | | - Male - Female - Non-binary - Prefer not to say |
| 15 |  | | | How old is your child? | | | Select one | | |  | | | - 5 - 6 - 7 - 8 - 9 - 10 - 11 | |
| 16 |  | | | Does your child have any of the following conditions? | | | Select many | | | Please select all that apply. | | | - Chronic respiratory disease - Chronic heart disease (including hypertension) - Chronic kidney disease - Chronic liver disease - Diabetes (Type 1, 2, or gestational) - Blood disease - Weakened immune system - Neurological disease - Mental health conditions - Obesity - Other - None of the above | |
| 17 |  | | | Does your child normally receive influenza vaccines? | | | Select one | | |  | | | - Yes - No - Unsure | |
| 18 |  | | | Has your child ever experienced side effects or allergic reactions (e.g. anaphylaxis) after vaccination? | | | Select one | | |  | | | - Yes, but minor symptoms (e.g. muscle aches, headache, fever) - Yes, serious adverse effects leading to ER visits or hospitalizations - No - I don’t know | |
| 19 |  | | | Has your child ever tested positive for COVID-19? | | | Select one | | |  | | | - Yes - No - Unsure | |
| 20 |  | | | Have your child/children aged 5 to 11 received at least one dose of the COVID-19 vaccine? | | | Select one | | |  | | | - Yes - No, but I've already booked a vaccination appointment - No | |
| 21 | If ‘No, but I've already booked a vaccination appointment’ OR ‘No’ in Q20 | | | Do you want to have your child/children vaccinated against COVID-19 if  vaccination is recommended and available for them? | | | Select one | | |  | | | - Yes, definitely - Unsure, but leaning towards yes - Unsure, but leaning towards no - No, definitely not | |
| 22 | If Unsure, but leaning towards yes OR Unsure, but leaning towards no OR No,  definitely not in Q21 | | | You indicated that you are either unsure or reluctant to the idea of your children receiving a COVID19 vaccine. Please briefly explain why. | | | Text | | |  | | |  | |
| 23 | If Unsure, but leaning towards yes OR Unsure, but leaning towards no OR No,  definitely not in Q21 | | Please tell us briefly what would change your mind about a COVID-19 vaccine and let your child be vaccinated? | | |  | | |  | | | |  | |
| 24 |  | | From the following list, please select who’s recommendation might convince you to let your children get vaccinated against COVID-19. | | | Select many | | |  | | | | - A friend - A family member - A politician I like - A celebrity I like - The Government - A doctor or healthcare worker - Other - I don’t know | |
| 25 |  | | “My child may get COVID-19 infection within the next 6 months.” | | | Select one | | | Please rate your level of agreement to the following statement. | | | | - Strongly agree - Agree - Neither agree nor disagree - Disagree - Strongly disagree | |
| 26 |  | | “COVID-19 is a  serious disease” | | | Select one | | | Please rate your level of agreement to the following statement. | | | | - Strongly agree - Agree - Neither agree nor disagree - Disagree - Strongly disagree | |
| 27 |  | | “I believe that my child getting a COVID-19 vaccine will ease my anxiety.” | | | Select one | | | Please rate your level of agreement to the following statement. | | | | - Strongly agree - Agree - Neither agree nor disagree - Disagree - Strongly disagree | |
| 28 |  | | “My child does not need to take a COVID-19 vaccine because he/she practises social distancing and washes hands frequently with soap or sanitizer, which can help prevent the spread of COVID- 19.” | | | Select one | | | Please rate your level of agreement to the following statement. | | | | - Strongly agree - Agree - Neither agree nor disagree - Disagree - Strongly disagree | |
| 29 |  | | “I believe vaccination of children can help control the spread of COVID-19.” | | | Select one | | | Please rate your level of agreement to the following statement. | | | | - Strongly agree - Agree - Neither agree nor disagree - Disagree - Strongly disagree | |
| 30 |  | | “My child will take a COVID-19 vaccine if many other children in Singapore are also vaccinated.” | | | Select one | | | Please rate your level of agreement to the following statement. | | | | - Strongly agree - Agree - Neither agree nor disagree - Disagree - Strongly disagree | |
| 31 |  | | “My child will take a COVID-19 vaccine if a COVID-19 vaccine certificate is required by law for school, travel, social events or dine-in.” | | | Select one | | | Please rate your level of agreement to the following statement. | | | - Strongly agree - Agree - Neither agree nor disagree - Disagree - Strongly disagree | | |
| 32 |  | | “COVID-19  vaccination should be compulsory for all children in Singapore.” | | | Select one | | | Please rate your level of agreement to the following statement. | | | - Strongly agree - Agree - Neither agree nor disagree - Disagree - Strongly disagree | | |
| 33 |  | | In the past month, on average, how much time per day have you spent actively using social media? | | | Select one | | |  | | | - I’m not a social media user - Less than 10 minutes per day - 11–30 minutes per day - 31–60 minutes per day - 1--2 hours per day - 2–3 hours per day - More than 3 hours per day | | |
| 34 |  | | In the past month, from which of these social media platforms did you receive information regarding COVID-19 vaccines? | | | Select many | | | Please select all that apply. | | | - Facebook - Twitter - YouTube - WhatsApp - LINE - Facebook Messenger - Instagram - TikTok - LinkedIn - Other | | |
| 35 |  | | From the following list, please select the source that you use and trust the MOST to learn about COVID-19 vaccines. | | | Select one | | |  | | | - Local public television (e.g. MediaCorp TV) - Radio - International television (e.g. CNN, BBC) - Newspapers or magazines - Social media (e.g. Facebook, Twitter, WhatsApp, etc.) - Local public health authorities, (e.g. Ministry of Health, Ministry of Education) - Healthcare workers - Religious leaders - International health authorities (such as the World Health Organisation) - Community organisations - Scientists - Government websites - The internet or search engines (e.g. Google, Yahoo, etc.) - Family and friends - Work, school, or college/university - I don’t know - Other | | |
| 36 |  | | Have you ever seen or heard any COVID- 19 associated information on social media which might have persuaded you NOT to vaccinate your children against COVID-19? | | | Select one | | |  | | | - Yes - No - I don’t know | | |
| 37 | If Yes in Q36 | | Please describe what COVID-19  associated information on social media persuaded you NOT to vaccinate your children against COVID-19. | | | Text | | |  | | |  | | |
| 38 |  | | “Genetic technology is used in COVID-19 vaccine to cause changes in genes  through vaccination” | | | Select one | | |  | | | - True - Unsure - False | | |
| 39 |  | | “Many people have died after they were vaccinated with a COVID-19 vaccine” | | | Select one | | |  | | | - True - Unsure - False | | |
| 40 |  | | “COVID-19  vaccination is associated with infertility and/or miscarriage” | | | Select one | | |  | | | - True - Unsure - False | | |
| 41 |  | | “COVID-19  vaccination causes COVID-19 infection to those who receive the vaccines and people around them” | | | Select one | | |  | | | - True - Unsure - False | | |
| 42 |  | | “COVID-19 vaccines were approved without completing the standard clinical trial checks due to  the pandemic” | | | Select one | | |  | | | - True - Unsure - False | | |
| 43 |  | | “The safety of COVID-19 vaccines has not been confirmed yet because clinical trials have not been completed” | | | Select one | | |  | | | - True - Unsure - False | | |
| 44 |  | | Thank you for completing the first task of this study - the baseline survey. Next, please be reminded to complete the following to qualify for the post- intervention survey | | | Select one | | |  | | | - I understand, and will start using the ‘D24H Covid Vaccine Chatbot’. | | |
|  |  | | about chatbot user experience.  In the next week, please 1) use the chatbot for more than ten minutes; 2) ensure that you have more than ten conversations with the chatbot.  Participants who meet the above requirements and complete the post- survey within a week will receive the  rewards as a “thank you” for fulfilling the tasks. (After verification, those who were unable to complete the tasks within the designated time will not be eligible for compensation). Thank you very much for your cooperation. | | |  | | |  | | |  | | |

| Task title | Post-questionnaire – Child group (control/intervention) |
| --- | --- |
| Target locations | Singapore |
| Submission goal | 200 control / 200 intervention |

| # | Conditional logic | Question | | Question type | | Hint | | Response options | | |
| --- | --- | --- | --- | --- | --- | --- | --- | --- | --- | --- |
| 1 |  | We appreciate your participation in this conversational COVID19 Chatbot evaluation. This is the third out of three tasks that you will need to complete. | | Select one | |  | | - I understand, continue | | |
| 2 |  | Have your child/children aged 5 to 11 received at least one dose of the COVID-19 vaccine? | | Select one | |  | | - Yes - No, but I've already booked a vaccination appointment - No | | |
| 3 | If Yes in Q2 | Please briefly tell us why you decided to get | | Text | |  | |  | | |
|  |  | your child/children vaccinated. | |  | |  | |  | | |
| 4 | If No in Q2 | Do you want your child/children to receive a COVID-19 vaccine in the future if vaccination is recommended and available for them? | | Select one | |  | | - Yes, definitely - Unsure, but leaning towards yes - Unsure, but leaning towards no - No, definitely not | | |
| 5 | If Yes definitely in Q4 | Please briefly tell us why you want your child/children to receive a COVID-19 vaccine. | | Text | |  | |  | | |
| 6 | If any response BUT Yes definitely in Q4 | Please briefly tell us why you are hesitant or reluctant to the idea of your child/children receiving a COVID19 vaccine. | | Text | |  | |  | | |
| 7 | If any response BUT Yes definitely in Q4 | From the following list, please select who’s recommendation might convince you to let your children get vaccinated against COVID-19. | | Select many | |  | | - A friend - A family member - A politician I like - A celebrity I like - The Government - A doctor or healthcare worker - Other - I don’t know | | |
| 8 | If Other in Q7 | Who else might convince you to let your child take a COVID-19 vaccine? | | Text | |  | |  | | |
| 9 |  | Would you be willing to have your child receive an annual booster against COVID-19 if it is recommended and available for them? | | Select one | |  | | - Yes, definitely. - Unsure, but leaning towards yes. - Unsure, but leaning towards no. - No, definitely not. | | |
| 10 |  | "Overall, I think COVID-19 vaccines are IMPORTANT for young children" | | Select one | | Please rate your level of agreement to the following statement. | | - Strongly agree - Tend to agree - Neither agree nor disagree - Tend to disagree - Strongly disagree | | |
| 11 |  | "Overall, I think COVID-19 vaccines are SAFE for young children." | | Select one | | Please rate your level of agreement to the following statement. | | - Strongly agree - Tend to agree - Neither agree nor disagree - Tend to disagree - Strongly disagree | | |
| 12 |  | "Overall, I think COVID-19 vaccines are EFFECTIVE at preventing infection." | | Select one | | Please rate your level of agreement to the following statement. | | - Strongly agree - Tend to agree - Neither agree nor disagree - Tend to disagree - Strongly disagree | | |
| 13 |  | “Overall, I think COVID-19 vaccines are effective in | | Select one | | Please rate your level of agreement | | - Strongly agree - Tend to agree - Neither agree nor disagree | | |
|  |  | reducing the risk of developing severe conditions.” | |  | | to the following statement. | | - Tend to disagree - Strongly disagree | | |
| 14 |  | “My child may get COVID-19 infection within the next 6 months.” | | Select one | | Please rate your level of agreement to the following statement. | | - Strongly agree - Agree - Neither agree nor disagree - Disagree - Strongly disagree | | |
| 15 |  | “COVID-19 is a serious disease” | | Select one | | Please rate your level of agreement to the following statement. | | - Strongly agree - Agree - Neither agree nor disagree - Disagree - Strongly disagree | | |
| 16 |  | “I believe that my child getting a COVID-19 vaccine will ease my anxiety.” | | Select one | | Please rate your level of agreement to the following statement. | | - Strongly agree - Agree - Neither agree nor disagree - Disagree - Strongly disagree | | |
| 17 |  | “My child does not need to take a COVID- 19 vaccine because he/she practises social distancing and washes hands frequently with soap or sanitizer, which can help prevent the spread of COVID-19.” | | Select one | | Please rate your level of agreement to the following statement. | | - Strongly agree - Agree - Neither agree nor disagree - Disagree - Strongly disagree | | |
| 18 |  | “I believe vaccination of children can help control the spread of COVID-19.” | | Select one | | Please rate your level of agreement to the following statement. | | - Strongly agree - Agree - Neither agree nor disagree - Disagree - Strongly disagree | | |
| 19 |  | “It is easy to find relevant information on COVID-19 vaccines.” | | Select one | | Please rate your level of agreement to the following statement. | | - Strongly agree - Agree - Neither agree nor disagree - Disagree - Strongly disagree | | |
| 20 |  | “My child will take a COVID-19 vaccine if many other children in Singapore are also vaccinated.” | | Select one | | Please rate your level of agreement to the following statement. | | - Strongly agree - Agree - Neither agree nor disagree - Disagree - Strongly disagree | | |
| 21 |  | “My child will take a COVID-19 vaccine if a COVID-19 vaccine certificate is required by law for school, travel, social events or dine-in.” | | Select one | | Please rate your level of agreement to the following statement. | | - Strongly agree - Agree - Neither agree nor disagree - Disagree - Strongly disagree | | |
| 22 |  | “COVID-19 vaccination should be compulsory for all citizens in Singapore.” | | Select one | | Please rate your level of agreement to the following statement. | | - Strongly agree - Agree - Neither agree nor disagree - Disagree - Strongly disagree | | |
| 23 |  | Have you ever seen or heard any COVID-19 associated information on social media which might have persuaded you NOT to immunise your children against COVID-19? | | Select one | |  | | - Yes - No - I don’t know | | |
| 24 | If Yes in Q23 | Please describe what COVID-19 associated information on social media persuaded you NOT to immunise your elderly family member against COVID-19. | | Text | |  | |  | | |
| 25 |  | Up next we will show you a set of statements and you will have to indicate if you think they are true, false or if you are unsure. | | Select one | |  | | - I understand, continue. | | |
| 26 |  | “Genetic technology is used in COVID-19 vaccine to cause changes in genes  through vaccination” | | Select one | |  | | - True - Unsure - False | | |
| 27 |  | “Many people have died after getting the COVID-19 vaccines” | | Select one | |  | | - True - Unsure - False | | |
| 28 |  | “COVID-19 vaccination is associated with infertility and/or miscarriage” | | Select one | |  | | - True - Unsure - False | | |
| 29 |  | “COVID-19 vaccination causes COVID-19 infection to those who receive the vaccines and people around them” | | Select one | |  | | - True - Unsure - False | | |
| 30 |  | “COVID-19 vaccines were approved without completing the normal process of the clinical trial” | | Select one | |  | | - True - Unsure - False | | |
| 31 |  | “The safety of COVID- 19 vaccines has not been confirmed yet because the clinical trial is not completed yet” | | Select one | |  | | - True - Unsure - False | | |
| 32 |  | Up next we will ask you to rate your level of agreement towards statements about your experience with the | | Select one | |  | | - I understand, continue. | | |
|  |  | D24H Covid vaccine chatbot. | |  | |  | |  | | |
| 33 |  | In the past week, how much time in total have you spent on the chatbot? [Best estimate] | | Select one | |  | | - I did not use the chatbot - Less than 10 minutes - 11-30 minutes - 31–60 minutes - 1-2 hours - 2-3 hours - More than 3 hours | | |
| 34 |  | What types of COVID19 related information were you seeking? | | Select many | | Please select all that apply. | | - Vaccine safety - Vaccine effectiveness - Vaccine appointments - Vaccine centre locations - Vaccine boosters - Vaccines and pregnancy - Other | | |
| 35 | If Other in Q34 | What other types of information were you seeking? | | Text | |  | |  | | |
| 36 |  | “I found the information I was searching for” | | Select one | |  | | - Strongly agree - Agree - Neither agree nor disagree - Disagree - Strongly disagree | | |
| 37 |  | “The chatbot had a quick response time to my question(s)” | | Select one | |  | | - Strongly agree - Agree - Neither agree nor disagree - Disagree - Strongly disagree | | |
| 38 |  | “I intend to use the chatbot again” | | Select one | |  | | - Strongly agree - Agree - Neither agree nor disagree - Disagree - Strongly disagree | | |
| 39 |  | “I like the design of the chatbot (font, colour, etc.)” | | Select one | |  | | - Strongly agree - Agree - Neither agree nor disagree - Disagree - Strongly disagree | | |
| 40 |  | “I intend to recommend the chatbot to my  friends and family.” | | Select one | |  | | - Strongly agree - Agree - Neither agree nor disagree - Disagree - Strongly disagree | | |
| 41 |  | On which platform would this chatbot be most helpful? | | Select one | |  | | - Ministry of Health website - Patient portal - Social media (e.g. WhatsApp, Telegram, etc.) - Other | | |
| 42 | If Other in Q41 | Where else do you think the chatbot would be most useful? | | Text | |  | |  | | |
| 43 |  | How could the chatbot be improved? | | Select many | | Please select all that apply. | | - Quicker responses - More detailed information - Change in style of chatbot (e.g. font, colour, size, etc.) - Other | | |
| 44 | If Other in Q43 | | What else could be done to improve the chatbot? | |  | |  | |  |  |

**Supplementary References**

1. Kwan BM, McGinnes HL, Ory MG, et al. RE-AIM in the Real World: Use of the RE-AIM Framework for Program Planning and Evaluation in Clinical and Community Settings. *Frontiers in Public Health* 2019;7 doi: 10.3389/fpubh.2019.00345

2. McDonald, R. P. & Ho, M. H. Principles and practice in reporting structural equation analyses. Psychol Methods 7, 64-82 (2002). <https://doi.org:10.1037/1082-989x.7.1.64>

3. Sun, J. Assessing Goodness of Fit in Confirmatory Factor Analysis. Measurement and Evaluation in Counseling and Development 37, 240-256 (2005). <https://doi.org:10.1080/07481756.2005.11909764>

4. Rosseel, Y. lavaan: An R Package for Structural Equation Modeling. Journal of Statistical Software 48, 1 - 36 (2012). <https://doi.org:10.18637/jss.v048.i02>
