# Supplementary material for: Effectiveness of chatbots on COVID vaccine confidence and acceptance in Thailand, Hong Kong, and Singapore
Source: NPJ Digit Med. 2023 May 25;6:96. doi: 10.1038/s41746-023-00843-6 (PMC10208906; doi:10.1038/s41746-023-00843-6)
Supplement: Supplementary file 2 — Reporting Summary [file 41746_2023_843_MOESM2_ESM.pdf]

nature portfolio

Corresponding author(s): Leesa Lin; NPJDIGITALMED-05053R

Last updated by author(s): Jan 30, 2023

Reporting Summary

Nature Portfolio wishes to improve the reproducibility of the work that we publish. This form provides structure for consistency and transparency in reporting. For further information on Nature Portfolio policies, see our Editorial Policies and the Editorial Policy Checklist.

Statistics

For all statistical analyses, confirm that the following items are present in the figure legend, table legend, main text, or Methods section.

n/a	Confirmed	
☐	☒	The exact sample size (*n*) for each experimental group/condition, given as a discrete number and unit of measurement
☐	☒	A statement on whether measurements were taken from distinct samples or whether the same sample was measured repeatedly
☐	☒	The statistical test(s) used AND whether they are one- or two-sided<br>*Only common tests should be described solely by name; describe more complex techniques in the Methods section.*
☐	☒	A description of all covariates tested
☐	☒	A description of any assumptions or corrections, such as tests of normality and adjustment for multiple comparisons
☐	☒	A full description of the statistical parameters including central tendency (e.g. means) or other basic estimates (e.g. regression coefficient) AND variation (e.g. standard deviation) or associated estimates of uncertainty (e.g. confidence intervals)
☒	☐	For null hypothesis testing, the test statistic (e.g. *F*, *t*, *r*) with confidence intervals, effect sizes, degrees of freedom and *P* value noted<br>*Give P values as exact values whenever suitable.*
☒	☐	For Bayesian analysis, information on the choice of priors and Markov chain Monte Carlo settings
☒	☐	For hierarchical and complex designs, identification of the appropriate level for tests and full reporting of outcomes
☒	☐	Estimates of effect sizes (e.g. Cohen's *d*, Pearson's *r*), indicating how they were calculated

Our web collection on statistics for biologists contains articles on many of the points above.

Software and code

Policy information about availability of computer code

Data collection	No software used
Data analysis	R version 4.2.2 and Diagrams.net 20.8.3

For manuscripts utilizing custom algorithms or software that are central to the research but not yet described in published literature, software must be made available to editors and reviewers. We strongly encourage code deposition in a community repository (e.g. GitHub). See the Nature Portfolio guidelines for submitting code & software for further information.

Data

Policy information about availability of data

All manuscripts must include a data availability statement. This statement should provide the following information, where applicable:

- Accession codes, unique identifiers, or web links for publicly available datasets
- A description of any restrictions on data availability
- For clinical datasets or third party data, please ensure that the statement adheres to our policy

Anonymized data and code used can be found at: https://github.com/lkwok/VCF_chatbot

Human research participants

Policy information about studies involving human research participants and Sex and Gender in Research.

Reporting on sex and gender	In this study, we asked for respondents' and their children or senior family members' sex (biological attribute) via online questionnaires with options of "Female", "Male", and "Other." We reported as respondents indicated in the questionnaires.
Population characteristics	The study participants included guardians of children and seniors from Thailand, Hong Kong and Singapore, who were delaying or rejecting their COVID-19 vaccinations. Sociodemographic factors, misinformation, risk perception were collected from guardians of unvaccinated children (134 Thai parents, 199 Hong Kong parents, and 95 Singapore parents) and seniors (guardians of 132 Thai seniors and 188 Hong Kong seniors).
Recruitment	Recruitment of participants in all three locations (Thailand, Hong Kong, and Singapore) were recruited by Premise, a participant recruitment and market research company. Since participants recruited by Premise were more familiar with surveys related to market research rather than vaccines, their indifference to domains of chatbot content might have led to user dissatisfaction and raised the drop-out rate.
Ethics oversight	This study was approved by the Institutional Review Board of The University of Hong Kong/ Hospital Authority Hong Kong West Cluster (UW 21-659), National University of Singapore, Saw Swee Hock School of Public Health Departmental Ethics Review Committee (SSHSPH-158), and the Ethics Committee of the Institute for the Development of Human Research Protections of the Ministry of Public Health, Thailand (IHRP 1122-2654). The study protocol was registered at ClinicalTrials.gov (ID: NCT05424952) and made publicly available on July 22, 2022. All study participants electronically signed the consent form.

Note that full information on the approval of the study protocol must also be provided in the manuscript.

Field-specific reporting

Please select the one below that is the best fit for your research. If you are not sure, read the appropriate sections before making your selection.

☐ Life sciences ☒ Behavioural & social sciences ☐ Ecological, evolutionary & environmental sciences

For a reference copy of the document with all sections, see nature.com/documents/nr-reporting-summary-flat.pdf

Behavioural & social sciences study design

All studies must disclose on these points even when the disclosure is negative.

Study description	This study is a multisite randomised controlled trial (RCT) designed to evaluate the effectiveness of COVID-19 chatbots for improving COVID-19 vaccine confidence and acceptance in Thailand, Hong Kong, and Singapore
Research sample	Thailand adult residents (Thai users) with (a) unvaccinated senior parents/grandparents aged 60 or above or (b) unvaccinated children aged below 18; Hong Kong adult residents (English or Chinese users) with (a) unvaccinated senior parents/grandparents aged 60 (including those who received first dose after the announcement of the COVID passport on 4th of January) or (b) unvaccinated children aged below 18; and Singapore adult residents (English or Chinese users) with unvaccinated children aged between 5 to 11 years old.
Sampling strategy	In all three locations, participants were recruited by Premise, a participant recruitment and market research company, via random sampling using existing online panels of participants.
Data collection	Eligible users in the online panel within a mobile application were randomly invited to join the study. After signing the consent form, participants were asked to complete the pre- and post-intervention questionnaires and to use the chatbot if assigned to the intervention group.
Timing	February 11th to June 30th, 2022
Data exclusions	We excluded respondents who are younger than 18 years, and those whose children or senior family members are already vaccinated at the time of recruitment.
Non-participation	948 participants did not return the consent forms and 332 participants dropped out and did not complete the study.
Randomization	Randomisation and group allocation were performed by a participant recruitment and market research company, Premise. The control or intervention group were allocated with a ratio of 1:1.

Reporting for specific materials, systems and methods

We require information from authors about some types of materials, experimental systems and methods used in many studies. Here, indicate whether each material, system or method listed is relevant to your study. If you are not sure if a list item applies to your research, read the appropriate section before selecting a response.

Materials & experimental systems

n/a	Involved in the study
☒	☐ Antibodies
☒	☐ Eukaryotic cell lines
☒	☐ Palaeontology and archaeology
☒	☐ Animals and other organisms
☒	☐ Clinical data
☒	☐ Dual use research of concern

Methods

n/a	Involved in the study
☒	☐ ChIP-seq
☒	☐ Flow cytometry
☒	☐ MRI-based neuroimaging
